# Supplementary material for: Deep Learning for RNA Secondary Structure Determination: Gauging Generalizability and Broadening the Scope of Traditional Methods
Source: bioRxiv. 2025 Nov 7:2025.11.04.686644. Preprint. [Version 1] doi: 10.1101/2025.11.04.686644 (PMC12637522; doi:10.1101/2025.11.04.686644)
Supplement: Supplement 1 [file media-1.pdf]

Supplemental Tables: Deep Learning for RNA Secondary Structure Determination: Gauging Generalizability and Broadening the Scope of Traditional Methods

Marcell Szikszai[1, 2], Ting-Yuan Wang[3], Ryan Krueger[4], David H. Mathews[5, 6], Max Ward[1], and Sharon Aviran[3, 7]

[1]Department of Computer Science and Software Engineering, The University of Western Australia, Crawley, WA 6009, Australia
[2]Department of Molecular and Cellular Biology, Harvard University, Cambridge, MA 02138, USA
[3]Department of Biomedical Engineering, University of California Davis, Davis, CA 95616, USA
[4]School of Engineering and Applied Sciences, Harvard University, Cambridge, MA 02138, USA
[5]Department of Biochemistry and Biophysics, University of Rochester Medical Center, Rochester, NY 14642, USA
[6]Center for RNA Biology, University of Rochester Medical Center, Rochester, NY 14642, USA
[7]Genome Center, University of California Davis, Davis, CA 95616, USA

Contents

1 Single-sequence benchmarking results **2**
- 1.1 Positive Predictive Value . . . 2
- 1.2 Sensitivity . . . 4
- 1.3 F-score . . . 6
- 1.4 Homology filtered results . . . 8
- 1.5 Statistical testing . . . 9

2 bpRNA-1m PDB set homology **10**

3 SPOT-RNA transfer learning PDB set homology **12**

4 SPOT-RNA & SPOT-RNA 2 results **14**
- 4.1 Positive Predictive Value . . . 14
- 4.2 Sensitivity . . . 16
- 4.3 F-score . . . 18
- 4.4 Homology filtered results . . . 20
- 4.5 Statistical testing . . . 21

5 Models for probing data prediction **22**

6 Method identification **23**
- 6.1 Semantic Scholar . . . 23
- 6.2 Google Scholar . . . 29

1 Single-sequence benchmarking results

1.1 Positive Predictive Value

	RNAstructure	ViennaRNA	EternaFold	MXfold2	SPOT-RNA	RNA-FM	RiNALMo
**Aptamers**							
Theophyline aptamer*	0.80	0.80	0.76	0.80	0.64	0.80	**0.88**
minE/minF aptamer	0.75	0.75	**0.82**	0.77	0.68‡	0.74	0.72
RhoBAST aptamer	**1.00**	**1.00**	0.94	**1.00**	0.81	0.97	0.88
Spinach aptamer	0.61	0.59	0.58	0.37	**0.75**†	0.59	0.53
Mango aptamer	0.73	0.73	**0.92**	0.91	0.73‡	0.87	0.89
Vitamin B12 aptamer	0.14	0.14	0.60	0.75	0.64†	0.14	**1.00**
Squash aptamer	0.84	**0.91**	0.87	**0.91**	0.89	0.70	0.81
Pepper aptamer	**1.00**	**1.00**	0.00	**1.00**	0.83	**1.00**	0.00
Chili aptamer	0.53	0.53	0.65	0.50	**0.80**	**0.80**	0.67
Corn aptamer	0.45	0.45	0.42	0.50	**0.64**‡	0.45	0.50
DIR2s aptamer	0.88	**1.00**	0.75	0.78	0.94‡	**1.00**	0.93
Clivia aptamer	0.55	0.57	0.71	**1.00**	0.71	0.80	0.67
A9g aptamer	**0.82**	0.45	0.50	0.45	0.42	0.55	0.67
Beetroot aptamer	**0.92**	**0.92**	**0.92**	**0.92**	0.61	0.83	0.59
Malachite green aptamer**	**1.00**	**1.00**	**1.00**	**1.00**	0.92‡	**1.00**	**1.00**
11F7t aptamer	0.90	0.90	**1.00**	0.90	0.85‡	**1.00**	0.62
K1 aptamer	**1.00**	**1.00**	**1.00**	**1.00**	0.70‡	**1.00**	0.75
Tetracycline aptamer	**1.00**	0.94	0.94	**1.00**	**1.00**‡	**1.00**	**1.00**
**CRISPR RNA guides**							
Cas9 guide	0.87	0.87	0.92	**0.93**	0.78	0.92	0.65
Cas12 guide	0.56	0.58	0.56	0.60	0.61	**0.74**	0.58
Cas13 guide	0.48	0.49	0.48	**0.51**	0.39‡	0.50	0.25
**IRES**							
IAPV IRES*	0.46	0.45	0.43	0.48	**0.52**†	0.51	0.35
CrPV 5'UTR IRES	0.22	0.21	0.28	0.22	0.22†	**0.34**	0.15
TSV IRES	0.29	0.29	0.48	0.51	0.56	0.51	**0.58**
PSIV IGR IRES	0.64	0.64	0.82	0.82	0.82‡	0.82	**1.00**
**Ribozymes**							
Synthetic ligase ribozyme	0.84	0.85	0.85	0.85	0.76‡	**0.88**	0.75
VS ribozyme**	0.62	0.90	0.83	0.92	**0.93**†	0.63	0.42
Self-alkylating ribozyme	**1.00**	**1.00**	0.83	**1.00**	0.80	0.86	0.18
Diels-Alder ribozyme	**1.00**	0.89	0.89	**1.00**	0.73	0.89	0.88
Methyltransferase ribozyme	**0.97**	0.87	0.95	**0.97**	0.93	0.90	0.88

Continued on next page

	**RNAstructure**	**ViennaRNA**	**EternaFold**	**MXfold2**	**SPOT-RNA**	**RNA-FM**	**RiNALMo**
**Disease-associated nucleotide repeats**							
r(CUG)**	**1.00**	**1.00**	**1.00**	**1.00**	0.75	**1.00**	**1.00**
r(CCUG)**	**1.00**	**1.00**	**1.00**	**1.00**	0.80‡	**1.00**	0.85
r(AUUCU)	**1.00**	**1.00**	**1.00**	**1.00**	0.77	**1.00**	**1.00**
**Miscellaneous synthetics**							
Nanoarchitecture 1	0.88	0.88	**0.93**	0.33	0.20	0.00	0.00
Nanoarchitecture 2	**0.95**	**0.95**	**0.95**	**0.95**	0.74	0.67	0.45
Synthetic hairpin	**1.00**	**1.00**	**1.00**	**1.00**	0.75	**1.00**	**1.00**
Synthetic tetraloop-tetraloop receptor	0.86	0.86	0.91	**0.94**	0.88‡	0.94	0.93
Synthetic G-quadruplex	0.62	0.62	0.62	**0.83**	0.64†	0.62	0.67
**Miscellaneous**							
ToxI	0.27	0.27	0.53	0.00	**0.58**‡	0.42	0.26
Structured part of acrIF8-aca2 5' UTR	0.92	0.92	0.92	0.75	**1.00**	**1.00**	**1.00**
Saguaro cactus viral mRNA structure	0.83	0.84	**0.97**	0.90	0.86	0.77	0.82
ITS-2	0.56	0.58	0.56	0.65	0.46	0.62	**0.82**
ENE	0.95	0.95	0.91	0.98	0.90†	0.98	**0.99**
ASH1 mRNA E3-localization element**	**0.80**	**0.80**	**0.80**	**0.80**	0.62‡	**0.80**	0.69
SCNMV xrRNA	**1.00**	**1.00**	0.73	0.78	0.75‡	0.90	0.83
Influenza B vRNA promoter	**0.36**	**0.36**	0.29	**0.36**	0.31	0.31	0.33
NAD-II riboswitch	0.44	0.44	0.68	0.50	**0.87**	0.25	0.39
**Descriptive statistics**							
Mean	0.75	0.75	0.76	**0.77**	0.71	0.74	0.68
Median	0.84	**0.86**	0.83	0.85	0.75	0.80	0.72
Min	0.14	0.14	0.00	0.00	**0.20**	0.00	0.00
Max	**1.00**	**1.00**	**1.00**	**1.00**	**1.00**	**1.00**	**1.00**
Std. Dev.	0.25	0.25	0.23	0.25	0.19	0.26	**0.28**

Supplemental Table S1. **Positive Predictive Value (PPV) on the proposed benchmarking dataset.** Values with † indicate homology to SPOT-RNA's PDB transfer learning dataset, while values with ‡ indicate exact match between PDB structures.

1.2 Sensitivity

	RNAstructure	ViennaRNA	EternaFold	MXfold2	SPOT-RNA	RNA-FM	RiNALMo
**Aptamers**							
Theophyline aptamer*	0.97	0.97	**1.00**	0.70	**1.00**	0.97	0.60
minE/minF aptamer	0.65	0.65	0.84	0.73	**0.92**‡	0.73	0.63
RhoBAST aptamer	0.78	**0.83**	**0.83**	0.81	0.72	0.74	0.62
Spinach aptamer	0.62	0.64	0.62	0.36	**0.86**†	0.44	0.36
Mango aptamer	0.83	0.83	**0.97**	**0.97**	**0.97**‡	**0.97**	0.66
Vitamin B12 aptamer	0.12	0.12	0.38	0.38	**0.88**†	0.12	0.12
Squash aptamer	**0.88**	**0.88**	0.83	**0.88**	0.67	0.67	0.54
Pepper aptamer	**0.94**	**0.94**	0.00	**0.94**	0.62	**0.94**	0.00
Chili aptamer	0.60	0.60	0.73	0.60	**0.80**	**0.80**	0.40
Corn aptamer	0.71	0.71	0.71	0.71	**1.00**‡	0.71	0.57
DIR2s aptamer	0.79	**0.89**	0.79	0.74	0.84‡	0.68	0.68
Clivia aptamer	0.74	0.82	**1.00**	**1.00**	0.82	**1.00**	0.65
A9g aptamer	**1.00**	0.56	0.67	0.56	0.56	0.67	0.67
Beetroot aptamer	**0.85**	**0.85**	**0.85**	**0.85**	0.46	0.62	0.38
Malachite green aptamer**	**1.00**	**1.00**	**1.00**	**1.00**	0.85‡	0.77	0.77
11F7t aptamer	0.82	0.82	**1.00**	0.82	**1.00**‡	0.91	0.45
K1 aptamer	**1.00**	**1.00**	**1.00**	**1.00**	**1.00**‡	**1.00**	0.86
Tetracycline aptamer	0.89	0.89	**0.94**	**0.94**	0.89‡	0.72	0.61
**CRISPR RNA guides**							
Cas9 guide	0.90	0.91	**0.96**	**0.96**	0.88	0.87	0.34
Cas12 guide	0.58	0.60	**0.62**	0.60	0.57	0.60	0.41
Cas13 guide	**0.60**	**0.60**	**0.60**	**0.60**	0.54‡	0.52	0.25
**IRES**							
IAPV IRES*	0.74	0.74	0.74	**0.75**	0.68†	0.64	0.30
CrPV 5'UTR IRES	0.52	0.52	**0.61**	0.52	0.25†	0.50	0.20
TSV IRES	0.44	0.44	**0.69**	0.64	0.64	0.62	0.36
PSIV IGR IRES	0.50	0.50	0.64	0.64	**1.00**‡	0.64	0.50
**Ribozymes**							
Synthetic ligase ribozyme	0.71	0.75	**0.77**	0.75	0.69‡	0.76	0.45
VS ribozyme**	0.59	0.93	0.89	**0.95**	0.82†	0.43	0.21
Self-alkylating ribozyme	**0.94**	**0.94**	0.83	**0.94**	0.67	0.67	0.11
Diels-Alder ribozyme	**1.00**	**1.00**	**1.00**	**1.00**	**1.00**	**1.00**	0.88
Methyltransferase ribozyme	0.88	0.82	**0.97**	0.85	0.88	0.82	0.49
**Disease-associated nucleotide repeats**							
r(CUG)**	0.83	0.83	0.92	0.92	**1.00**	0.92	0.67

Continued on next page

	RNAstructure	ViennaRNA	EternaFold	MXfold2	SPOT-RNA	RNA-FM	RiNALMo
r(CCUG)**	**1.00**	**1.00**	**1.00**	0.88	0.94‡	**1.00**	0.65
r(AUUCU)	**0.83**	**0.83**	**0.83**	0.75	**0.83**	**0.83**	0.75
**Miscellaneous synthetics**							
Nanoarchitecture 1	**1.00**	**1.00**	**1.00**	0.29	0.21	0.00	0.00
Nanoarchitecture 2	**1.00**	**1.00**	**1.00**	**1.00**	0.85	0.60	0.23
Synthetic hairpin	0.67	0.67	**1.00**	**1.00**	**1.00**	**1.00**	0.50
Synthetic tetraloop-tetraloop receptor	**1.00**	**1.00**	**1.00**	**1.00**	**1.00**‡	0.97	0.90
Synthetic G-quadruplex	0.71	0.71	0.71	0.71	**1.00**†	0.71	0.57
**Miscellaneous**							
ToxI	0.19	0.19	0.38	0.00	**0.71**‡	0.40	0.23
Structured part of acrIF8-aca2 5' UTR	**1.00**	**1.00**	**1.00**	0.75	0.75	0.75	0.58
Saguaro cactus viral mRNA structure	0.76	0.79	**0.91**	0.82	0.87	0.65	0.48
ITS-2	**0.89**	**0.89**	0.83	**0.89**	0.68	**0.89**	0.83
ENE	**1.00**	**1.00**	**1.00**	0.99	**1.00**†	0.92	0.82
ASH1 mRNA E3-localization element**	**1.00**	**1.00**	**1.00**	**1.00**	0.83‡	**1.00**	0.75
SCNMV xrRNA	0.89	**1.00**	0.89	0.78	**1.00**‡	**1.00**	0.56
Influenza B vRNA promoter	**0.67**	**0.67**	**0.67**	**0.67**	**0.67**	**0.67**	**0.67**
NAD-II riboswitch	0.41	0.41	0.60	0.40	**0.80**	0.20	0.20
**Descriptive statistics**							
Mean	0.78	0.78	**0.81**	0.77	0.80	0.72	0.50
Median	0.83	0.83	0.84	0.81	**0.84**	0.73	0.54
Min	0.12	0.12	0.00	0.00	**0.21**	0.00	0.00
Max	**1.00**	**1.00**	**1.00**	**1.00**	**1.00**	**1.00**	0.90
Std. Dev.	0.21	0.21	0.21	0.22	0.19	**0.24**	0.23

Supplemental Table S2. **Sensitivity on the proposed benchmarking dataset.** Values with † indicate homology to SPOT-RNA's PDB transfer learning dataset, while values with ‡ indicate exact match between PDB structures.

1.3 F-score

	RNAstructure	ViennaRNA	EternaFold	MXfold2	SPOT-RNA	RNA-FM	RiNALMo
**Aptamers**							
Theophyline aptamer*	**0.87**	**0.87**	0.85	0.73	0.77	**0.87**	0.69
minE/minF aptamer	0.69	0.69	**0.82**	0.75	0.78‡	0.73	0.67
RhoBAST aptamer	0.88	**0.91**	0.88	0.90	0.76	0.84	0.73
Spinach aptamer	0.61	0.62	0.60	0.36	**0.80**†	0.50	0.43
Mango aptamer	0.78	0.78	**0.95**	0.94	0.83‡	0.92	0.74
Vitamin B12 aptamer	0.13	0.13	0.46	0.50	**0.74**†	0.13	0.22
Squash aptamer	0.86	**0.89**	0.85	**0.89**	0.76	0.68	0.65
Pepper aptamer	**0.97**	**0.97**	0.00	**0.97**	0.71	**0.97**	0.00
Chili aptamer	0.56	0.56	0.69	0.55	**0.80**	**0.80**	0.50
Corn aptamer	0.56	0.56	0.53	0.59	**0.78**‡	0.56	0.53
DIR2s aptamer	0.83	**0.94**	0.77	0.76	0.89‡	0.81	0.79
Clivia aptamer	0.63	0.68	0.83	**1.00**	0.76	0.89	0.64
A9g aptamer	**0.90**	0.50	0.57	0.50	0.48	0.60	0.67
Beetroot aptamer	**0.88**	**0.88**	**0.88**	**0.88**	0.52	0.70	0.47
Malachite green aptamer**	**1.00**	**1.00**	**1.00**	**1.00**	0.88‡	0.87	0.87
11F7t aptamer	0.86	0.86	**1.00**	0.86	0.92‡	0.95	0.53
K1 aptamer	**1.00**	**1.00**	**1.00**	**1.00**	0.82‡	**1.00**	0.80
Tetracycline aptamer	0.94	0.91	0.94	**0.97**	0.94‡	0.84	0.76
**CRISPR RNA guides**							
Cas9 guide	0.89	0.89	0.94	**0.95**	0.83	0.90	0.42
Cas12 guide	0.57	0.59	0.59	0.60	0.58	**0.64**	0.47
Cas13 guide	0.53	0.53	0.52	**0.55**	0.45‡	0.50	0.25
**IRES**							
IAPV IRES*	0.56	0.56	0.54	0.58	**0.58**†	0.57	0.32
CrPV 5'UTR IRES	0.31	0.29	0.38	0.31	0.23†	**0.41**	0.17
TSV IRES	0.35	0.35	0.57	0.57	**0.60**	0.56	0.44
PSIV IGR IRES	0.56	0.56	0.72	0.72	**0.90**‡	0.72	0.67
**Ribozymes**							
Synthetic ligase ribozyme	0.77	0.80	0.81	0.80	0.73‡	**0.81**	0.55
VS ribozyme**	0.61	0.92	0.86	**0.94**	0.87†	0.51	0.28
Self-alkylating ribozyme	**0.97**	**0.97**	0.83	**0.97**	0.73	0.75	0.14
Diels-Alder ribozyme	**1.00**	0.94	0.94	**1.00**	0.84	0.94	0.88
Methyltransferase ribozyme	0.93	0.84	**0.96**	0.91	0.90	0.85	0.63
**Disease-associated nucleotide repeats**							
r(CUG)**	0.91	0.91	**0.96**	**0.96**	0.86	**0.96**	0.80

Continued on next page

	RNAstructure	ViennaRNA	EternaFold	MXfold2	SPOT-RNA	RNA-FM	RiNALMo
r(CCUG)**	**1.00**	**1.00**	**1.00**	0.94	0.86‡	**1.00**	0.73
r(AUUCU)	**0.91**	**0.91**	**0.91**	0.86	0.80	**0.91**	0.86
**Miscellaneous synthetics**							
Nanoarchitecture 1	0.93	0.93	**0.97**	0.31	0.21	0.00	0.00
Nanoarchitecture 2	**0.98**	**0.98**	**0.98**	**0.98**	0.79	0.63	0.30
Synthetic hairpin	0.80	0.80	**1.00**	**1.00**	0.86	**1.00**	0.67
Synthetic tetraloop-tetraloop receptor	0.92	0.92	0.95	**0.97**	0.94‡	0.95	0.92
Synthetic G-quadruplex	0.67	0.67	0.67	0.77	**0.78**†	0.67	0.62
**Miscellaneous**							
ToxI	0.22	0.22	0.44	0.00	**0.64**‡	0.41	0.25
Structured part of acrIF8-aca2 5' UTR	**0.96**	**0.96**	**0.96**	0.75	0.86	0.86	0.74
Saguaro cactus viral mRNA structure	0.79	0.81	**0.93**	0.86	0.87	0.70	0.61
ITS-2	0.68	0.70	0.67	0.75	0.55	0.73	**0.82**
ENE	0.97	0.97	0.95	**0.98**	0.95†	0.95	0.89
ASH1 mRNA E3-localization element**	**0.89**	**0.89**	**0.89**	**0.89**	0.71‡	**0.89**	0.72
SCNMV xrRNA	0.94	**1.00**	0.80	0.78	0.86‡	0.95	0.67
Influenza B vRNA promoter	**0.47**	**0.47**	0.40	**0.47**	0.42	0.42	0.44
NAD-II riboswitch	0.42	0.42	0.64	0.44	**0.83**	0.22	0.26
**Descriptive statistics**							
Mean	0.75	0.76	**0.77**	0.76	0.74	0.72	0.56
Median	**0.86**	**0.86**	0.85	0.86	0.79	0.80	0.63
Min	0.13	0.13	0.00	0.00	**0.21**	0.00	0.00
Max	**1.00**	**1.00**	**1.00**	**1.00**	0.95	**1.00**	0.92
Std. Dev.	0.23	0.23	0.22	0.23	0.17	0.24	**0.24**

Supplemental Table S3. **F-score on the proposed benchmarking dataset.** Values with † indicate homology to SPOT-RNA's PDB transfer learning dataset, while values with ‡ indicate exact match between PDB structures.

1.4 Homology filtered results

	RNAstructure	ViennaRNA	EternaFold	MXfold2	SPOT-RNA	RNA-FM	RiNALMo
Mean	0.741	0.731	0.742	**0.754**	0.701	0.732	0.665
Median	0.841	**0.853**	0.826	0.841	0.760	0.809	0.691
Min	0.143	0.143	0.000	0.000	**0.200**	0.000	0.000
Max	**1.000**	**1.000**	**1.000**	**1.000**	**1.000**	**1.000**	**1.000**
Std. Dev.	0.257	0.258	0.239	0.261	0.205	0.265	**0.288**

Supplemental Table S4. Descriptive statistics of PPV on the benchmarking dataset with all homology removed. All overlaps found in Table S8 (*, **) and Table S9 (†, ‡) are excluded from this calculation.

	RNAstructure	ViennaRNA	EternaFold	MXfold2	SPOT-RNA	RNA-FM	RiNALMo
Mean	0.758	0.757	**0.792**	0.746	0.726	0.708	0.488
Median	0.804	0.821	**0.833**	0.765	0.734	0.718	0.500
Min	0.125	0.125	0.000	0.000	**0.214**	0.000	0.000
Max	**1.000**	**1.000**	**1.000**	**1.000**	**1.000**	**1.000**	0.900
Std. Dev.	0.219	0.220	0.215	0.231	0.181	**0.238**	0.236

Supplemental Table S5. Descriptive statistics of sensitivity on the benchmarking dataset with all homology removed. All overlaps found in Table S8 (*, **) and Table S9 (†, ‡) are excluded from this calculation.

	RNAstructure	ViennaRNA	EternaFold	MXfold2	SPOT-RNA	RNA-FM	RiNALMo
Mean	0.740	0.735	**0.758**	0.742	0.702	0.710	0.544
Median	0.817	0.807	**0.827**	0.787	0.761	0.741	0.612
Min	0.133	0.133	0.000	0.000	**0.207**	0.000	0.000
Max	**1.000**	**1.000**	**1.000**	**1.000**	0.903	**1.000**	0.915
Std. Dev.	0.232	0.235	0.225	0.243	0.178	0.241	**0.243**

Supplemental Table S6. Descriptive statistics of F-score on the benchmarking dataset with all homology removed. All overlaps found in Table **S8** (*, **) and Table **S9** (†, ‡) are excluded from this calculation.

1.5 Statistical testing

	ViennaRNA	EternaFold	MXfold2	SPOT-RNA	RNA-FM	RiNALMo
**RNAstructure**	6.43e-01	5.92e-01	9.59e-01	1.15e-01	3.45e-01	2.82e-05
**ViennaRNA**		4.75e-01	8.00e-01	1.80e-01	4.24e-01	5.42e-05
**EternaFold**			6.51e-01	2.08e-01	2.35e-01	4.02e-07
**MXfold2**				5.28e-02	1.94e-01	6.55e-06
**SPOT-RNA**					8.68e-01	2.19e-03
**RNA-FM**						6.83e-06

Supplemental Table S7. The p-values from paired t-tests between F-score of the different methods. All overlaps found in Table **S8** (*, **) and Table **S9** (†, ‡) are excluded from this calculation. Note that there are no corrections, so the values must be carefully interpreted to avoid the multiple testing problem. Red values are statistically significant ($p < 0.05$).

2 bpRNA-1m PDB set homology

	bpRNA-1m Entry
**Aptamers**	
Theophyline aptamer	1eht_A, 1o15_A
minE/minF aptamer	
RhoBAST aptamer	
Spinach aptamer	
Mango aptamer	
Vitamin B12 aptamer	
Squash aptamer	
Pepper aptamer	
Chili aptamer	
Corn aptamer	
DIR2s aptamer	
Clivia aptamer	
A9g aptamer	
Beetroot aptamer	
Malachite green aptamer	1f1t_A**, 1q8n_A
11F7t aptamer	
K1 aptamer	
Tetracycline aptamer	
**CRISPR RNA guides**	
Cas9 guide	
Cas12 guide	
Cas13 guide	
**IRES**	
IAPV IRES	2n8v_X
CrPV 5'UTR IRES	
TSV IRES	
PSIV IGR IRES	
**Ribozymes**	
Synthetic ligase ribozyme	
VS ribozyme	4r4v_A**, 2n3r_A, 2mtj_A, 1tbk_A, 1ow9_A, 2mtk_A, 2mis_A, 1tjz_A, 2n3q_A, 4r4p_A, 2l5z_A, 1yn1_A, 1hwq_A, 1yn2_A
Self-alkylating ribozyme	
Diels-Alder ribozyme	
Methyltransferase ribozyme	
**Disease-associated nucleotide repeats**	
r(CUG)	4pcj_A**, 4fnj_A

Continued on next page

	bpRNA-1m Entry
r(CCUG)<br>r(AUUCU)	4k27_U**
**Miscellaneous synthetics**	
Nanoarchitecture 1	
Nanoarchitecture 2	
Synthetic hairpin	
Synthetic tetraloop-tetraloop receptor	
Synthetic G-quadruplex	
**Miscellaneous**	
ToxI	
Structured part of acrIF8-aca2 5' UTR	
Saguaro cactus viral mRNA structure	
ITS-2	
ENE	
ASH1 mRNA E3-localization element	5m0h_A**
SCNMV xrRNA	
Influenza B vRNA promoter	
NAD-II riboswitch	

Supplemental Table S8. Overlapping PDB IDs between our benchmarking dataset and bpRNA-1m's PDB dataset. Entries with ** indicate an exact match (i.e. exact PDB chain are present in both datasets), while regular entries indicate related structures found during manual curation. Note that author chain IDs are added (after underscore) to the PDB ID.

3 SPOT-RNA transfer learning PDB set homology

	SPOT-RNA Entry
**Aptamers**	
Theophyline aptamer	
minE/minF aptamer	4m4o_B[‡]
RhoBAST aptamer	
Spinach aptamer	4kzd_R
Mango aptamer	6e8s_B[‡], 6c63_A
Vitamin B12 aptamer	1ddy_A
Squash aptamer	
Pepper aptamer	
Chili aptamer	
Corn aptamer	5bjo_Y[‡]
DIR2s aptamer	6db8_R[‡]
Clivia aptamer	
A9g aptamer	
Beetroot aptamer	
Malachite green aptamer	1f1t_A[‡], 6gzk_A
11F7t aptamer	5voe_A[‡]
K1 aptamer	6sy4_C[‡]
Tetracycline aptamer	3egz_B[‡]
**CRISPR RNA guides**	
Cas9 guide	
Cas12 guide	
Cas13 guide	6dtd_C[‡], 6aay_B[‡]
**IRES**	
IAPV IRES	2n8v_X
CrPV 5'UTR IRES	4v84_AV
TSV IRES	
PSIV IGR IRES	4v83_CV[‡]
**Ribozymes**	
Synthetic ligase ribozyme	3hhn_E[‡]
VS ribozyme	4r4p_A, 2mtj_A
Self-alkylating ribozyme	
Diels-Alder ribozyme	
Methyltransferase ribozyme	
**Disease-associated nucleotide repeats**	
r(CUG)	
r(CCUG)	4k27_U[‡]
r(AUUCU)	
**Miscellaneous synthetics**	
Nanoarchitecture 1	

Continued on next page

	SPOT-RNA Entry
Nanoarchitecture 2	
Synthetic hairpin	
Synthetic tetraloop-tetraloop receptor	6dvk_H‡
Synthetic G-quadruplex	5de5_A
**Miscellaneous**	
ToxI	2xd0_G, 4ato_G, 4rmo_H‡
Structured part of acrIF8-aca2 5' UTR	
Saguaro cactus viral mRNA structure	
ITS-2	
ENE	4plx_A
ASH1 mRNA E3-localization element	5m0h_A‡
SCNMV xrRNA	6d3p_A‡
Influenza B vRNA promoter	
NAD-II riboswitch	

Supplemental Table S9. **Overlapping PDB IDs between our benchmarking dataset and SPOT-RNA's transfer learning dataset.** Entries with ‡ indicate an exact match (i.e. exact PDB chain are present in both datasets), while regular entries indicate related structures found during manual curation. Note that author chain IDs are added (after underscore) to the PDB ID.

4 SPOT-RNA & SPOT-RNA 2 results

4.1 Positive Predictive Value

	RNAstructure	ViennaRNA	SPOT-RNA	SPOT-RNA 2
**Aptamers**				
Theophyline aptamer*	**0.80**	**0.80**	0.64	0.62
minE/minF aptamer	**0.75**	**0.75**	0.68‡	0.68
RhoBAST aptamer	**1.00**	**1.00**	0.81	0.81
Spinach aptamer	0.61	0.59	**0.75**†	0.65
Mango aptamer	**0.73**	**0.73**	0.73‡	0.71
Vitamin B12 aptamer	0.14	0.14	**0.64**†	**0.64**
Squash aptamer	0.84	**0.91**	0.89	0.57
Pepper aptamer	**1.00**	**1.00**	0.83	0.83
Chili aptamer	0.53	0.53	**0.80**	**0.80**
Corn aptamer	0.45	0.45	**0.64**‡	**0.64**
DIR2s aptamer	0.88	**1.00**	0.94‡	0.89
Clivia aptamer	0.55	0.57	**0.71**	0.69
A9g aptamer	**0.82**	0.45	0.42	0.42
Beetroot aptamer	**0.92**	**0.92**	0.61	0.79
Malachite green aptamer**	**1.00**	**1.00**	0.92‡	0.85
11F7t aptamer	**0.90**	**0.90**	0.85‡	0.00
K1 aptamer	**1.00**	**1.00**	0.70‡	0.70
Tetracycline aptamer	**1.00**	0.94	**1.00**‡	0.89
**CRISPR RNA guides**				
Cas9 guide	**0.87**	0.87	0.78	0.82
Cas12 guide	0.56	0.58	**0.61**	0.54
Cas13 guide	0.48	**0.49**	0.39‡	0.42
**IRES**				
IAPV IRES*	0.46	0.45	**0.52**†	nan
CrPV 5'UTR IRES	**0.22**	0.21	0.22†	nan
TSV IRES	0.29	0.29	**0.56**	nan
PSIV IGR IRES	0.64	0.64	**0.82**‡	0.81
**Ribozymes**				
Synthetic ligase ribozyme	0.84	**0.85**	0.76‡	0.73
VS ribozyme**	0.62	0.90	**0.93**†	nan
Self-alkylating ribozyme	**1.00**	**1.00**	0.80	0.80
Diels-Alder ribozyme	**1.00**	0.89	0.73	0.70
Methyltransferase ribozyme	**0.97**	0.87	0.93	0.85

Continued on next page

	RNAstructure	ViennaRNA	SPOT-RNA	SPOT-RNA 2
**Disease-associated nucleotide repeats**				
r(CUG)**	**1.00**	**1.00**	0.75	0.75
r(CCUG)**	**1.00**	**1.00**	0.80‡	0.77
r(AUUCU)	**1.00**	**1.00**	0.77	0.77
**Miscellaneous synthetics**				
Nanoarchitecture 1	**0.88**	**0.88**	0.20	0.20
Nanoarchitecture 2	**0.95**	**0.95**	0.74	0.74
Synthetic hairpin	**1.00**	**1.00**	0.75	0.86
Synthetic tetraloop-tetraloop receptor	0.86	0.86	**0.88**‡	**0.88**
Synthetic G-quadruplex	0.62	0.62	**0.64**†	0.42
**Miscellaneous**				
ToxI	0.27	0.27	**0.58**‡	0.57
Structured part of acrIF8-aca2 5' UTR	0.92	0.92	**1.00**	**1.00**
Saguaro cactus viral mRNA structure	0.83	0.84	**0.86**	**0.86**
ITS-2	0.56	**0.58**	0.46	0.46
ENE	**0.95**	**0.95**	0.90†	0.89
ASH1 mRNA E3-localization element**	**0.80**	**0.80**	0.62‡	0.00
SCNMV xrRNA	**1.00**	**1.00**	0.75‡	0.75
Influenza B vRNA promoter	**0.36**	**0.36**	0.31	0.31
NAD-II riboswitch	0.44	0.44	**0.87**	0.79
**Descriptive statistics**				
Mean	**0.75**	0.75	0.71	0.67
Median	0.84	**0.86**	0.75	0.74
Min	0.14	0.14	**0.20**	0.00
Max	**1.00**	**1.00**	**1.00**	**1.00**
Std. Dev.	0.25	**0.25**	0.19	0.23

Supplemental Table S10. **Positive Predictive Value (PPV) on the proposed benchmarking dataset for SPOT-RNA and SPOT-RNA 2.** Values with † indicate homology to SPOT-RNA's PDB transfer learning dataset, while values with ‡ indicate exact match between PDB structures. § indicates structures that we were unable to predict with SPOT-RNA 2 (see *Materials and Methods* for mode information).

4.2 Sensitivity

	RNAstructure	ViennaRNA	SPOT-RNA	SPOT-RNA 2
**Aptamers**				
Theophyline aptamer*	0.97	0.97	**1.00**	**1.00**
minE/minF aptamer	0.65	0.65	**0.92**‡	**0.92**
RhoBAST aptamer	0.78	**0.83**	0.72	0.72
Spinach aptamer	0.62	0.64	**0.86**†	0.74
Mango aptamer	0.83	0.83	**0.97**‡	**0.97**
Vitamin B12 aptamer	0.12	0.12	**0.88**†	**0.88**
Squash aptamer	**0.88**	**0.88**	0.67	0.67
Pepper aptamer	**0.94**	**0.94**	0.62	**0.94**
Chili aptamer	0.60	0.60	**0.80**	**0.80**
Corn aptamer	0.71	0.71	**1.00**‡	**1.00**
DIR2s aptamer	0.79	**0.89**	0.84‡	0.84
Clivia aptamer	0.74	0.82	0.82	**0.88**
A9g aptamer	**1.00**	0.56	0.56	0.56
Beetroot aptamer	**0.85**	**0.85**	0.46	0.65
Malachite green aptamer**	**1.00**	**1.00**	0.85‡	0.85
11F7t aptamer	0.82	0.82	**1.00**‡	0.00
K1 aptamer	**1.00**	**1.00**	**1.00**‡	**1.00**
Tetracycline aptamer	**0.89**	**0.89**	**0.89**‡	**0.89**
**CRISPR RNA guides**				
Cas9 guide	0.90	0.91	0.88	**0.96**
Cas12 guide	0.58	**0.60**	0.57	0.57
Cas13 guide	0.60	0.60	0.54‡	**0.62**
**IRES**				
IAPV IRES*	**0.74**	**0.74**	0.68†	nan
CrPV 5'UTR IRES	**0.52**	**0.52**	0.25†	nan
TSV IRES	0.44	0.44	**0.64**	nan
PSIV IGR IRES	0.50	0.50	**1.00**‡	0.93
**Ribozymes**				
Synthetic ligase ribozyme	0.71	**0.75**	0.69‡	0.74
VS ribozyme**	0.59	**0.93**	0.82†	nan
Self-alkylating ribozyme	**0.94**	**0.94**	0.67	0.67
Diels-Alder ribozyme	**1.00**	**1.00**	**1.00**	**1.00**
Methyltransferase ribozyme	**0.88**	0.82	**0.88**	0.82
**Disease-associated nucleotide repeats**				
r(CUG)**	0.83	0.83	**1.00**	**1.00**

Continued on next page

	**RNAstructure**	**ViennaRNA**	**SPOT-RNA**	**SPOT-RNA 2**
r(CCUG)**	**1.00**	**1.00**	0.94‡	**1.00**
r(AUUCU)	**0.83**	**0.83**	**0.83**	**0.83**
**Miscellaneous synthetics**				
Nanoarchitecture 1	**1.00**	**1.00**	0.21	0.21
Nanoarchitecture 2	**1.00**	**1.00**	0.85	0.85
Synthetic hairpin	0.67	0.67	**1.00**	**1.00**
Synthetic tetraloop-tetraloop receptor	**1.00**	**1.00**	**1.00**‡	**1.00**
Synthetic G-quadruplex	0.71	0.71	**1.00**†	0.71
**Miscellaneous**				
ToxI	0.19	0.19	**0.71**‡	0.63
Structured part of acrIF8-aca2 5' UTR	**1.00**	**1.00**	0.75	0.75
Saguaro cactus viral mRNA structure	0.76	0.79	**0.87**	**0.87**
ITS-2	**0.89**	**0.89**	0.68	0.68
ENE	**1.00**	**1.00**	**1.00**†	**1.00**
ASH1 mRNA E3-localization element**	**1.00**	**1.00**	0.83‡	0.00
SCNMV xrRNA	0.89	**1.00**	**1.00**‡	**1.00**
Influenza B vRNA promoter	**0.67**	**0.67**	**0.67**	**0.67**
NAD-II riboswitch	0.41	0.41	**0.80**	**0.80**
**Descriptive statistics**				
Mean	0.78	0.78	**0.80**	0.78
Median	0.83	0.83	**0.84**	**0.84**
Min	0.12	0.12	**0.21**	0.00
Max	**1.00**	**1.00**	**1.00**	**1.00**
Std. Dev.	0.21	0.21	0.19	**0.24**

Supplemental Table S11. **Sensitivity on the proposed benchmarking dataset for SPOT-RNA and SPOT-RNA 2.** Values with † indicate homology to SPOT-RNA's PDB transfer learning dataset, while values with ‡ indicate exact match between PDB structures. § indicates structures that we were unable to predict with SPOT-RNA 2 (see *Materials and Methods* for mode information).

4.3 F-score

	**RNAstructure**	**ViennaRNA**	**SPOT-RNA**	**SPOT-RNA 2**
**Aptamers**				
Theophyline aptamer[*]	**0.87**	**0.87**	0.77	0.76
minE/minF aptamer	0.69	0.69	**0.78**[‡]	**0.78**
RhoBAST aptamer	0.88	**0.91**	0.76	0.76
Spinach aptamer	0.61	0.62	**0.80**[†]	0.69
Mango aptamer	0.78	0.78	**0.83**[‡]	0.81
Vitamin B12 aptamer	0.13	0.13	**0.74**[†]	**0.74**
Squash aptamer	0.86	**0.89**	0.76	0.62
Pepper aptamer	**0.97**	**0.97**	0.71	0.88
Chili aptamer	0.56	0.56	**0.80**	**0.80**
Corn aptamer	0.56	0.56	**0.78**[‡]	**0.78**
DIR2s aptamer	0.83	**0.94**	0.89[‡]	0.86
Clivia aptamer	0.63	0.68	0.76	**0.77**
A9g aptamer	**0.90**	0.50	0.48	0.48
Beetroot aptamer	**0.88**	**0.88**	0.52	0.71
Malachite green aptamer[**]	**1.00**	**1.00**	0.88[‡]	0.85
11F7t aptamer	0.86	0.86	**0.92**[‡]	0.00
K1 aptamer	**1.00**	**1.00**	0.82[‡]	0.82
Tetracycline aptamer	**0.94**	0.91	**0.94**[‡]	0.89
**CRISPR RNA guides**				
Cas9 guide	0.89	**0.89**	0.83	0.88
Cas12 guide	0.57	**0.59**	0.58	0.54
Cas13 guide	0.53	**0.53**	0.45[‡]	0.50
**IRES**				
IAPV IRES[*]	0.56	0.56	**0.58**[†]	nan
CrPV 5'UTR IRES	**0.31**	0.29	0.23[†]	nan
TSV IRES	0.35	0.35	**0.60**	nan
PSIV IGR IRES	0.56	0.56	**0.90**[‡]	0.87
**Ribozymes**				
Synthetic ligase ribozyme	0.77	**0.80**	0.73[‡]	0.73
VS ribozyme[**]	0.61	**0.92**	0.87[†]	nan
Self-alkylating ribozyme	**0.97**	**0.97**	0.73	0.73
Diels-Alder ribozyme	**1.00**	0.94	0.84	0.82
Methyltransferase ribozyme	**0.93**	0.84	0.90	0.83
**Disease-associated nucleotide repeats**				
r(CUG)[**]	**0.91**	**0.91**	0.86	0.86

Continued on next page

	**RNAstructure**	**ViennaRNA**	**SPOT-RNA**	**SPOT-RNA 2**
r(CCUG)**	**1.00**	**1.00**	0.86‡	0.87
r(AUUCU)	**0.91**	**0.91**	0.80	0.80
**Miscellaneous synthetics**				
Nanoarchitecture 1	**0.93**	**0.93**	0.21	0.21
Nanoarchitecture 2	**0.98**	**0.98**	0.79	0.79
Synthetic hairpin	0.80	0.80	0.86	**0.92**
Synthetic tetraloop-tetraloop receptor	0.92	0.92	**0.94**‡	**0.94**
Synthetic G-quadruplex	0.67	0.67	**0.78**†	0.53
**Miscellaneous**				
ToxI	0.22	0.22	**0.64**‡	0.60
Structured part of acrIF8-aca2 5' UTR	**0.96**	**0.96**	0.86	0.86
Saguaro cactus viral mRNA structure	0.79	0.81	**0.87**	**0.87**
ITS-2	0.68	**0.70**	0.55	0.55
ENE	**0.97**	**0.97**	0.95†	0.94
ASH1 mRNA E3-localization element**	**0.89**	**0.89**	0.71‡	0.00
SCNMV xrRNA	0.94	**1.00**	0.86‡	0.86
Influenza B vRNA promoter	**0.47**	**0.47**	0.42	0.42
NAD-II riboswitch	0.42	0.42	**0.83**	0.79
**Descriptive statistics**				
Mean	0.75	**0.76**	0.74	0.71
Median	**0.86**	**0.86**	0.79	0.79
Min	0.13	0.13	**0.21**	0.00
Max	**1.00**	**1.00**	0.95	0.94
Std. Dev.	0.23	**0.23**	0.17	0.22

Supplemental Table S12. **F-score on the proposed benchmarking dataset for SPOT-RNA and SPOT-RNA 2.** Values with † indicate homology to SPOT-RNA's PDB transfer learning dataset, while values with ‡ indicate exact match between PDB structures. § indicates structures that we were unable to predict with SPOT-RNA 2 (see *Materials and Methods* for mode information).

4.4 Homology filtered results

	**RNAstructure**	**ViennaRNA**	**SPOT-RNA**	**SPOT-RNA 2**
Mean	**0.741**	0.731	0.701	0.681
Median	0.841	**0.853**	0.760	0.736
Min	0.143	0.143	**0.200**	0.000
Max	**1.000**	**1.000**	**1.000**	**1.000**
Std. Dev.	0.257	**0.258**	0.205	0.212

Supplemental Table S13. Descriptive statistics of PPV on the benchmarking dataset with all homology removed for SPOT-RNA and SPOT-RNA 2. All overlaps found in Table S8 (*, **) and Table S9 (†, ‡) are excluded from this calculation.

	**RNAstructure**	**ViennaRNA**	**SPOT-RNA**	**SPOT-RNA 2**
Mean	0.758	0.757	0.726	**0.783**
Median	0.804	0.821	0.734	**0.825**
Min	0.125	0.125	**0.214**	0.000
Max	**1.000**	**1.000**	**1.000**	**1.000**
Std. Dev.	0.219	**0.220**	0.181	0.213

Supplemental Table S14. Descriptive statistics of sensitivity on the benchmarking dataset with all homology removed for SPOT-RNA and SPOT-RNA 2. All overlaps found in Table S8 (*, **) and Table S9 (†, ‡) are excluded from this calculation.

	RNAstructure	ViennaRNA	SPOT-RNA	SPOT-RNA 2
Mean	**0.740**	0.735	0.702	0.720
Median	**0.817**	0.807	0.761	0.782
Min	0.133	0.133	**0.207**	0.000
Max	**1.000**	**1.000**	0.903	0.942
Std. Dev.	0.232	**0.235**	0.178	0.202

Supplemental Table S15. **Descriptive statistics of F-score on the benchmarking dataset with all homology removed for SPOT-RNA and SPOT-RNA 2.** All overlaps found in Table **S8** (*, **) and Table **S9** (†, ‡) are excluded from this calculation.

4.5 Statistical testing

	ViennaRNA	SPOT-RNA	SPOT-RNA 2
RNAstructure	6.43e-01	1.15e-01	3.32e-01
ViennaRNA		1.80e-01	3.78e-01
SPOT-RNA			6.19e-01

Supplemental Table S16. **The p-values from paired t-tests between F-scores of SPOT-RNA and SPOT-RNA 2.** All overlaps found in Table **S8** (*, **) and Table **S9** (†, ‡) are excluded from this calculation. Note that there are no corrections, so the values must be carefully interpreted to avoid the multiple testing problem. No values are statistically significant ($p < 0.05$).

5 Models for probing data prediction

**Dataset**	**MAE**
SARS-CoV-2	0.177
HIV-1	0.201
E. coli	0.235
Homo Sapiens	0.183

Supplemental Table S17. **Mean Absolute Error (MAE) for each dataset.** MAE values were computed across all nucleotides in a dataset. Sequences were not computationally or experimentally padded, and no flanking sequences or barcodes were included.

6 Method identification

6.1 Semantic Scholar

Title	Authors	Year	DOI	Citations	Citations/Year	Relevance	Method
RNA secondary structure prediction using deep learning with thermodynamic integration	Sato et al.	2021	10.1038/s41467-021-21194-4	290	72.50	✓	MXfold2
RiNALMo: general-purpose RNA language models can generalize well on structure prediction tasks	Penić et al.	2024	10.1038/s41467-025-60872-5	38	38.00	✓	RiNALMo
UFold: fast and accurate RNA secondary structure prediction with deep learning	Fu et al.	2020	10.1093/nar/gkab1074	169	33.80	✓	Ufold

Continued on next page

Title	Authors	Year	DOI	Citations	Citations/Year	Relevance	Method
Recent trends in RNA informatics: a review of machine learning and deep learning for RNA secondary structure prediction and RNA drug discovery	Sato et al.	2023	10.1093/bib/bbad186	48	24.00	✓	Review
RNA Secondary Structure Prediction By Learning Unrolled Algorithms	Chen et al.	2020	10.48550/arXiv.2002.05810	119	23.80	✓	E2Efold
All-atom RNA structure determination from cryo-EM maps.	Li et al.	2024	10.1038/s41587-024-02149-8	23	23.00	✗	
Informative RNA base embedding for RNA structural alignment and clustering by deep representation learning	Akiyama et al.	2022	10.1093/nargab/lqac012	68	22.67	✗	
Predicting dynamic cellular protein–RNA interactions by deep learning using in vivo RNA structures	Sun et al.	2021	10.1038/s41422-021-00476-y	83	20.75	✗	

Continued on next page

Title	Authors	Year	DOI	Citations	Citations/Year	Relevance	Method
Improved RNA secondary structure and tertiary base-pairing prediction using evolutionary profile, mutational coupling and two-dimensional transfer learning	Singh et al.	2021	10.1093/bioinformatics/btab165	81	20.25	✓	SPOT-RNA 2
Deep learning models for RNA secondary structure prediction (probably) do not generalize across families	Szikszai et al.	2022	10.1093/bioinformatics/btac415	56	18.67	✓	Review
Machine learning for RNA 2D structure prediction benchmarked on experimental data	Justyna et al.	2023	10.1093/bib/bbad153	32	16.00	✓	Review
Review of machine learning methods for RNA secondary structure prediction	Zhao et al.	2020	10.1371/journal.pcbi.1009291	65	13.00	✓	Review

Continued on next page

Title	Authors	Year	DOI	Citations	Citations/Year	Relevance	Method
REDfold: accurate RNA secondary structure prediction using residual encoder-decoder network	Chen et al.	2023	10.1186/s12859-023-05238-8	25	12.50	✓	REDfold
gRNAde: Geometric Deep Learning for 3D RNA inverse design	Joshi et al.	2023	10.1101/2024.03.31.587283	19	9.50	✗	
Detecting protein and DNA/RNA structures in cryo-EM maps of intermediate resolution using deep learning	Wang et al.	2021	10.1038/s41467-021-22577-3	32	8.00	✗	
Deep learning predicts short non-coding RNA functions from only raw sequence data	Noviello et al.	2020	10.1371/journal.pcbi.1008415	33	6.60	✗	

Continued on next page

Title	Authors	Year	DOI	Citations	Citations/Year	Relevance	Method
Predicting RNA distance-based contact maps by integrated deep learning on physics-inferred secondary structure and evolutionary-derived mutational coupling	Singh et al.	2022	10.1093/bioinformatics/btac421	18	6.00	✗	
Caveats to Deep Learning Approaches to RNA Secondary Structure Prediction	Flamm et al.	2021	10.3389/fbinf.2022.835422	22	5.50	✓	Review
Improved Predicting of The Sequence Specificities of RNA Binding Proteins by Deep Learning	Tayara et al.	2020	10.1109/tcbb.2020.2981335	22	4.40	✗	

Continued on next page

Title	Authors	Year	DOI	Citations	Citations/Year	Relevance	Method
Deep neural networks for inferring binding sites of RNA-binding proteins by using distributed representations of RNA primary sequence and secondary structure	Deng et al.	2020	10.1186/s12864-020-07239-w	21	4.20	✗	

Supplemental Table S18. **Semantic Scholar method identification.** Top 20 papers on Semantic Scholar sorted by citation count for the search term “deep learning RNA secondary structure” released after 2020 (retrieved 2025-08-27).

6.2 Google Scholar

Title	Authors	Year	DOI	Citations	Citations/Year	Relevance	Method
RNA secondary structure prediction using deep learning with thermodynamic integration	Sato et al.	2021	10.1038/s41467-021-21194-4	445	111.25	✓	MXfold2
Geometric deep learning of RNA structure	Townshend et al.	2021	10.1126/science.abe5650	415	103.75	✗	
UFold: fast and accurate RNA secondary structure prediction with deep learning	Fu et al.	2022	10.1093/nar/gkab1074	237	79.00	✓	Ufold
Advances and opportunities in RNA structure experimental determination and computational modeling	Zhang et al.	2022	10.1038/s41592-022-01623-y	141	47.00	✓	Review

Continued on next page

Title	Authors	Year	DOI	Citations	Citations/Year	Relevance	Method
Integrating end-to-end learning with deep geometrical potentials for ab initio RNA structure prediction	Li et al.	2023	10.1038/s41467-023-41303-9	83	41.50	✗	
RNA secondary structure packages evaluated and improved by high-throughput experiments	Wayment-Steele et al.	2022	10.1038/s41592-022-01605-0	117	39.00	✓	Review
Predicting dynamic cellular protein–RNA interactions by deep learning using in vivo RNA structures	Sun et al.	2021	10.1038/s41422-021-00476-y	131	32.75	✗	
Recent trends in RNA informatics: a review of machine learning and deep learning for RNA secondary structure prediction and RNA drug discovery	Sato et al.	2023	10.1093/bib/bbad186	63	31.50	✓	Review

Continued on next page

Title	Authors	Year	DOI	Citations	Citations/Year	Relevance	Method
RNA secondary structure prediction by learning unrolled algorithms	Chen et al.	2020	10.48550/arXiv.2002.05810	155	31.00	✓	E2Efold
De Novo RNA Tertiary Structure Prediction at Atomic Resolution Using Geometric Potentials from Deep Learning	Pearce et al.	2022	10.1101/2022.05.15.491755	89	29.67	✗	
A deep learning approach to programmable RNA switches	Angenent-Mari et al.	2020	10.1038/s41467-020-18677-1	148	29.60	✗	
Protein–RNA interaction prediction with deep learning: structure matters	Wei et al.	2022	10.1093/bib/bbab540	83	27.67	✗	
Review of machine learning methods for RNA secondary structure prediction	Zhao et al.	2021	10.1371/journal.pcbi.1009291	107	26.75	✓	Review

Continued on next page

Title	Authors	Year	DOI	Citations	Citations/Year	Relevance	Method
Deep learning models for RNA secondary structure prediction (probably) do not generalize across families	Szikszai et al.	2022	10.1093/bioinformatics/btac415	80	26.67	✓	Review
E2Efold-3D: end-to-end deep learning method for accurate de novo RNA 3D structure prediction	Shen et al.	2022	10.48550/arXiv.2207.01586	80	26.67	✗	
Improved RNA secondary structure and tertiary base-pairing prediction using evolutionary profile, mutational coupling and two-dimensional transfer learning	Singh et al.	2021	10.1093/bioinformatics/btab165	100	25.00	✓	SPOT-RNA 2
Caveats to deep learning approaches to RNA secondary structure prediction	Flamm et al.	2022	10.3389/fbinf.2022.835422	33	11.00	✓	Review

Continued on next page

Title	Authors	Year	DOI	Citations	Citations/Year	Relevance	Method
Deep Learning Method for RNA Secondary Structure Prediction with Pseudoknots Based on Large-Scale Data	Shen et al.	2021	10.1155/2021/6699996	8	2.00	✓	Unamed
A survey on deep learning in DNA/RNA motif mining	He et al.	2021	10.1093/bib/bbaa229	0	0.00	✗	
trRosettaRNA: automated prediction of RNA 3D structure with transformer network	Wang et al.	2023	10.1038/s41467-023-42528-4	0	0.00	✗	

Supplemental Table S19. **Google Scholar method identification.** Top 20 papers on Google Scholar sorted by relevance count for the search term "deep learning RNA secondary structure" released after 2020 (retrieved 2025-08-27).
