# Supplementary material for: Deep Learning for RNA Secondary Structure Determination: Gauging Generalizability and Broadening the Scope of Traditional Methods
Source: bioRxiv. 2025 Nov 7:2025.11.04.686644. Preprint. [Version 1] doi: 10.1101/2025.11.04.686644 (PMC12637522; doi:10.1101/2025.11.04.686644)
Supplement: Supplement 4 [file media-4.pdf]

Supplemental File 1: Deep Learning for RNA Secondary Structure Determination: Gauging Generalizability and Broadening the Scope of Traditional Methods

Marcell Szikszai[1, 2], Ting-Yuan Wang[3], Ryan Krueger[4], David H. Mathews[5, 6], Max Ward[1], and Sharon Aviran[3, 7]

[1]Department of Computer Science and Software Engineering, The University of Western Australia, Crawley, WA 6009, Australia
[2]Department of Molecular and Cellular Biology, Harvard University, Cambridge, MA 02138, USA
[3]Department of Biomedical Engineering, University of California Davis, Davis, CA 95616, USA
[4]School of Engineering and Applied Sciences, Harvard University, Cambridge, MA 02138, USA
[5]Department of Biochemistry and Biophysics, University of Rochester Medical Center, Rochester, NY 14642, USA
[6]Center for RNA Biology, University of Rochester Medical Center, Rochester, NY 14642, USA
[7]Genome Center, University of California Davis, Davis, CA 95616, USA

Contents

1 Aptamers . . . 6
1.1 Theophyline aptamer . . . 6
1.1.1 8d28_A . . . 6
1.1.2 8d29_C . . . 7
1.1.3 8dk7_C . . . 8
1.2 minE/minF aptamer . . . 9
1.2.1 4m4o_B . . . 9
1.2.2 4m6d_L . . . 10
1.3 RhoBAST aptamer . . . 11
1.3.1 9bun_A . . . 11
1.3.2 8jy0_D . . . 12
1.4 Spinach aptamer . . . 13
1.4.1 6b14_R . . . 13

1.4.2 6b3k_R . . . 14
1.5 Mango aptamer . . . 15
1.5.1 8u5t_A . . . 15
1.5.2 6e8u_B . . . 16
1.5.3 6e8t_D . . . 17
1.5.4 8u5j_A . . . 18
1.5.5 6c65_B . . . 19
1.5.6 6e8s_B . . . 20
1.6 Vitamin B12 aptamer . . . 21
1.6.1 1et4_E . . . 21
1.7 Squash aptamer . . . 22
1.7.1 7kvu_G . . . 22
1.8 Pepper aptamer . . . 23
1.8.1 7eoh_A . . . 23
1.9 Chili aptamer . . . 24
1.9.1 7oax_D . . . 24
1.10 Corn aptamer . . . 25
1.10.1 5bjo_Y . . . 25
1.11 DIR2s aptamer . . . 26
1.11.1 6db8_R . . . 26
1.12 Clivia aptamer . . . 27
1.12.1 8hze_B . . . 27
1.12.2 8hzj_A . . . 28
1.12.3 8hzl_B . . . 29
1.13 A9g aptamer . . . 30
1.13.1 6rti_X . . . 30
1.14 Beetroot aptamer . . . 31
1.14.1 8eyu_B . . . 31
1.14.2 8f0n_B . . . 32
1.15 Malachite green aptamer . . . 33
1.15.1 1f1t_A . . . 33
1.16 11F7t aptamer . . . 34
1.16.1 5voe_A . . . 34
1.17 K1 aptamer . . . 35
1.17.1 6sy4_C . . . 35
1.18 Tetracycline aptamer . . . 36
1.18.1 3egz_B . . . 36

2 CRISPR guides **37**

2.1 Cas9 guide . . . 37
2.1.1 7el1_B . . . 37
2.1.2 6wbr_B . . . 38
2.1.3 8umf_B . . . 39
2.1.4 8hud_B . . . 40
2.2 sgRNA guide . . . 41
2.2.1 8rdu_1 . . . 41
2.2.2 8x5v_B . . . 42
2.2.3 7c7l_C . . . 43
2.2.4 5wti_B . . . 44
2.3 Cas12 guide . . . 45
2.3.1 8bf8_B . . . 45
2.3.2 8j3r_C . . . 46
2.3.3 6xmf_C . . . 47
2.3.4 8dc2_B . . . 48
2.4 Cas13 guide . . . 49
2.4.1 6dtd_C . . . 49
2.4.2 8wcs_G . . . 50
2.4.3 6aay_B . . . 51
2.4.4 8ewg_B . . . 52
2.5 OMEGA effector guide . . . 53
2.5.1 8gkh_W . . . 53
2.6 Fanzor guide . . . 54
2.6.1 9cf2_W . . . 54

3 IRES **55**
3.1 IAPV IRES . . . 55
3.1.1 6p5i_1 . . . 55
3.1.2 6p5n_1 . . . 56
3.2 CrPV 5'UTR IRES . . . 57
3.2.1 6w2t_A . . . 57
3.3 TSV IRES . . . 58
3.3.1 8evp_EC . . . 58
3.4 PSIV IGR IRES . . . 59
3.4.1 4v83_CV . . . 59

4 Ribozymes **60**
4.1 Synthetic ligase ribozyme . . . 60
4.1.1 3hhn_E . . . 60

4.1.2 3ivk_C 61
4.1.3 8t2p_B 62
4.2 VS ribozyme 63
4.2.1 4r4v_A 63
4.3 Self-alkylating ribozyme 64
4.3.1 6xjq_A 64
4.4 Diels-Alder ribozyme 65
4.4.1 1ykv_D 65
4.4.2 1yls_D 66
4.5 Methyltransferase ribozyme 67
4.5.1 7dlz_Y 67
4.5.2 7v9e_A 68

5 Repeats **69**
5.1 r(CUG) 69
5.1.1 4pcj_A 69
5.2 r(CCUG) 70
5.2.1 4k27_U 70
5.3 r(AUUCU) 71
5.3.1 5btm_A 71

6 Miscellaneous (synthetic) **72**
6.1 Nanoarchitecture 1 72
6.1.1 7jrr_A 72
6.2 Nanoarchitecture 2 73
6.2.1 7jrs_A 73
6.3 Synthetic hairpin 74
6.3.1 6az4_A 74
6.4 Synthetic tetraloop-tetraloop receptor 75
6.4.1 6dvk_H 75
6.5 Synthetic G-quadruplex 76
6.5.1 5dea_C 76

7 Miscellaneous **77**
7.1 ToXI 77
7.1.1 7d8o_L 77
7.1.2 2xdb_G 78
7.1.3 4rmo_H 79
7.2 Structured part of acrIF8-aca2 5' UTR 80
7.2.1 8w35_C 80

7.3 Saguaro cactus viral mRNA structure 81
7.3.1 8t2a_R 81
7.3.2 8t29_R 82
7.3.3 8t2b_R 83
7.4 ITS-2 84
7.4.1 7r6q_6 84
7.4.2 7u0h_6 85
7.5 ENE 86
7.5.1 7ljy_B 86
7.5.2 3p22_A 87
7.5.3 7jnh_B 88
7.5.4 3p22_E 89
7.6 ASH1 mRNA E3-localization element 90
7.6.1 5m0h_A 90
7.7 SCNMV xrRNA 91
7.7.1 6d3p_A 91
7.8 Influenza B vRNA promoter 92
7.8.1 6t2c_V 92
7.9 NAD-II riboswitch 93
7.9.1 8hb8_A 93
7.9.2 8hba_A 94

1 Aptamers

1.1 Theophyline aptamer

1.1.1 8d28_A

Crystal structure of theophylline aptamer in complex with theophylline

Release date: 2022-11-30
Method: x-ray diffraction
Resolution: 1.42 Å
Chain length: 33
Extracted length: 33

Total clashes: 4
Clashes per residue: 0.121
Clashscore: 1.840

Description: RNA (33-MER)
Organism: synthetic construct

Menichelli, E., Lam, B.J., Wang, Y., Wang, V.S., Shaffer, J., Tjhung, K.F., Bursulaya, B., Nguyen, T.N., Vo, T., Alper, P.B., McAllister, C.S., Jones, D.H., Spraggon, G., Michellys, P.Y., Joslin, J., Joyce, G.F., Rogers, J. (2022) Discovery of small molecules that target a tertiary-structured RNA. Proc.Natl.Acad.Sci.USA.
DOI: 10.1073/pnas.2213117119

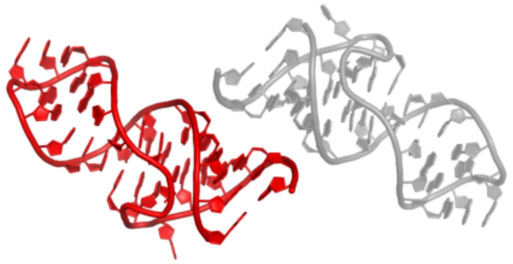

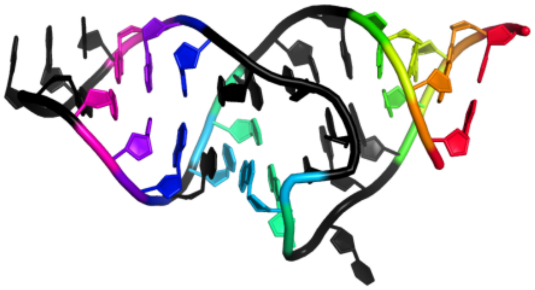

```
GGCGAUACCAGCCGAAAGGCCCUUGGCAGCGCC
((((...((.(((....)))....))...))))
```

1.1.2 8d29_C

Crystal structure of theophylline aptamer - apo form

Release date: 2022-11-30
Method: x-ray diffraction
Resolution: 1.81 Å
Chain length: 34
Extracted length: 34

Total clashes: 4
Clashes per residue: 0.118
Clashscore: 4.310

Description: RNA (34-MER)
Organism: synthetic construct

Menichelli, E., Lam, B.J., Wang, Y., Wang, V.S., Shaffer, J., Tjhung, K.F., Bursulaya, B., Nguyen, T.N., Vo, T., Alper, P.B., McAllister, C.S., Jones, D.H., Spraggon, G., Michellys, P.Y., Joslin, J., Joyce, G.F., Rogers, J. (2022) Discovery of small molecules that target a tertiary-structured RNA. Proc.Natl.Acad.Sci.USA.
DOI: 10.1073/pnas.2213117119

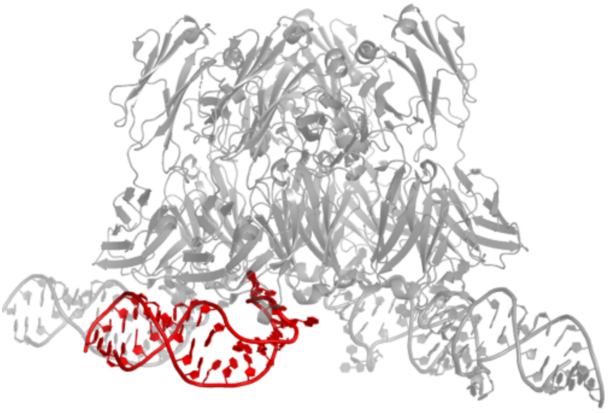

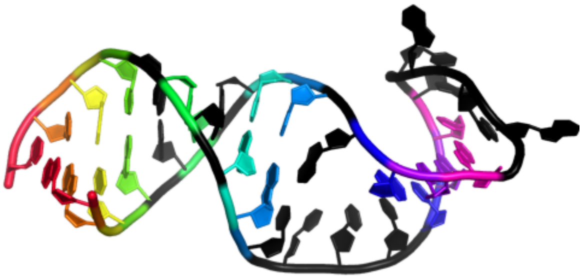

```
GGCGAUACCAGCGAAACACGCCCUUGGCAGCGUC
((((.(.((.(((.....)))....)).).))))
```

1.1.3 8dk7_C

Crystal structure of theophylline aptamer soaked with TAL2

Release date: 2022-11-30
Method: x-ray diffraction
Resolution: 2.46 Å
Chain length: 34
Extracted length: 34

Total clashes: 4
Clashes per residue: 0.118
Clashscore: 24.590

Description: RNA (34-MER)
Organism: synthetic construct

Menichelli, E., Lam, B.J., Wang, Y., Wang, V.S., Shaffer, J., Tjhung, K.F., Bursulaya, B., Nguyen, T.N., Vo, T., Alper, P.B., McAllister, C.S., Jones, D.H., Spraggon, G., Michellys, P.Y., Joslin, J., Joyce, G.F., Rogers, J. (2022) Discovery of small molecules that target a tertiary-structured RNA. Proc.Natl.Acad.Sci.USA.
DOI: 10.1073/pnas.2213117119

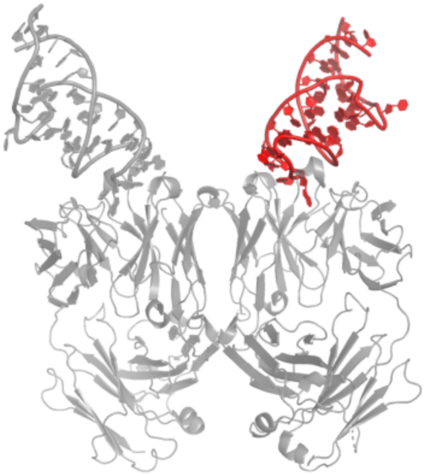

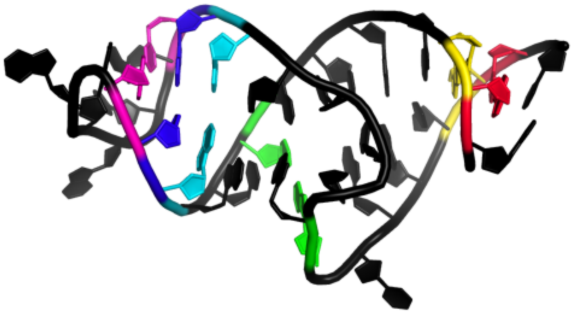

```
GGCGAUACCAGCGAAACACGCCCUUGGCAGCGUC
.((....(..(((.....))).....)....)).
```

1.2 minE/minF aptamer

1.2.1 4m4o_B

Crystal structure of the aptamer minE-lysozyme complex

Release date: 2013-12-18
Method: x-ray diffraction
Resolution: 2.00 Å
Chain length: 59
Extracted length: 59

Total clashes: 14
Clashes per residue: 0.237
Clashscore: 2.830

Description: RNA (59-MER)
Organism: nan

Malashkevich, V.N., Padlan, F.C., Toro, R., Girvin, M., Almo, S.C. (N/A) Crystal structure of the aptamer minE-lysozyme complex. to be published.
DOI: nan

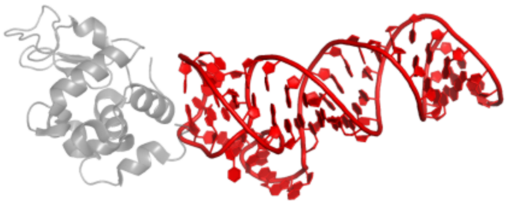

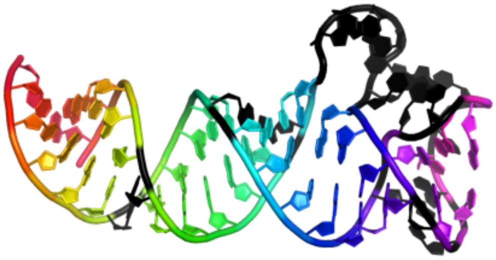

GGGUUCAUCAGGGCUAAAGAGUGCAGAGUUACUUAGUUCACUGCAGACUUGACGAACCC
((((((.(((((.((.......((((.(.(.<..)>..).)))))).))))).))))))

1.2.2 4m6d_L

Crystal structure of the aptamer minF-lysozyme complex.

Release date: 2013-12-11
Method: x-ray diffraction
Resolution: 2.68 Å
Chain length: 45
Extracted length: 43

Total clashes: 62
Clashes per residue: 1.378
Clashscore: 18.470

Description: aptamer
Organism: nan

Malashkevich, V.N., Padlan, F.C., Toro, R., Girvin, M., Almo, S.C. (N/A) Crystal structure of the aptamer minF-lysozyme complex. To be Published.
DOI: nan

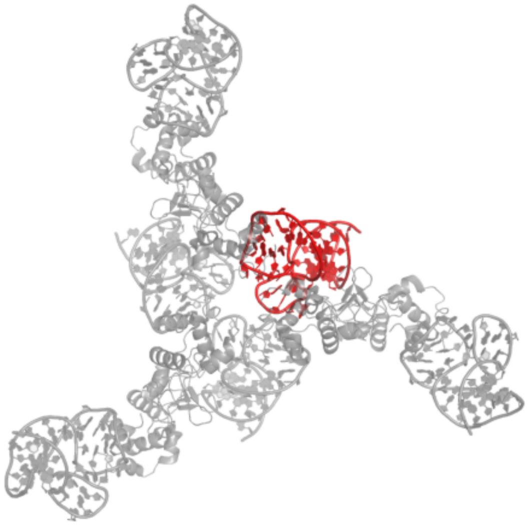

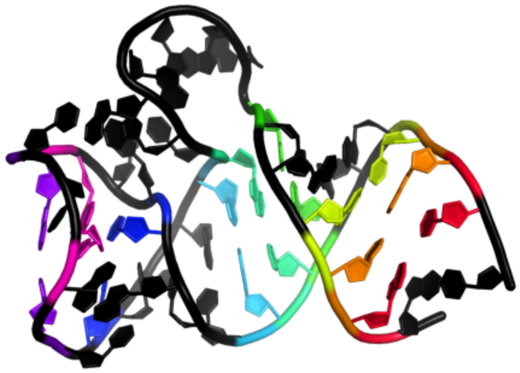

GGGCGGCUAAAGAGUGCAGAGUUACUUAGUUCACUGCAGACGCCC
..(((..(.......((...(.(.<..)>..)...)))..)))..

1.3 RhoBAST aptamer

1.3.1 9bun_A

RhoBAST aptamer RNA in complex with 5(6)-carboxytetramethylrhodamine

Release date: 2024-06-05
Method: x-ray diffraction
Resolution: 2.10 Å
Chain length: 48
Extracted length: 48

Total clashes: 9
Clashes per residue: 0.188
Clashscore: 11.130

Description: RNA (48-MER)
Organism: synthetic construct

Batey, R.T., Siwik, S.H. (N/A) Structure of RhoBAST RNA aptamer in complex with 5(6)-carboxytetramethylrhodamine (TAMRA). To Be Published.
DOI: nan

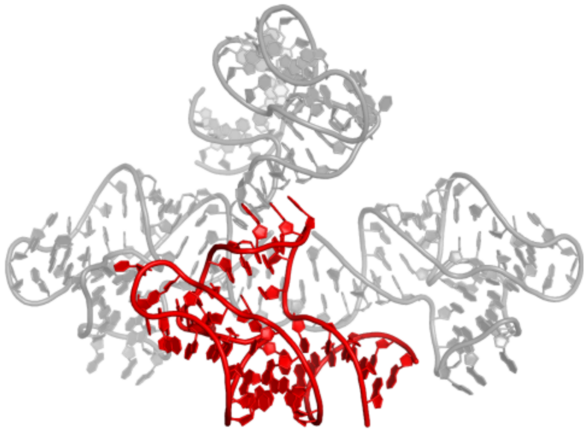

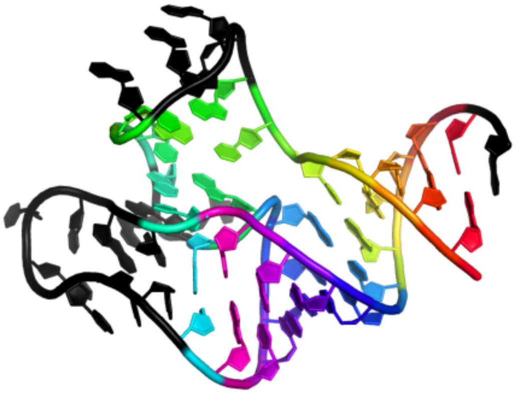

GGACUCGGAAACGUGAAGGAGAGGCGCAAGGUUAACCGCCUCAGUCCA
(((((((....))(<...<)((((((>........>))))))))))).

1.3.2 8jy0_D

Crystal structure of RhoBAST complexed with TMR-DN

Release date: 2024-05-29
Method: x-ray diffraction
Resolution: 2.75 Å
Chain length: 64
Extracted length: 64

Total clashes: 41
Clashes per residue: 0.641
Clashscore: 5.150

Description: RhoBAST
Organism: artificial sequences

Zhang, Y., Xu, Z., Xiao, Y., Jiang, H., Zuo, X., Li, X., Fang, X. (2024) Structural mechanisms for binding and activation of a contact-quenched fluorophore by RhoBAST. Nat Commun.
DOI: 10.1038/s41467-024-48478-9

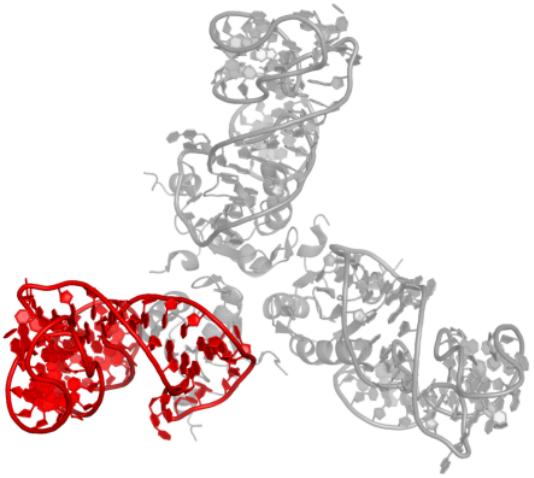

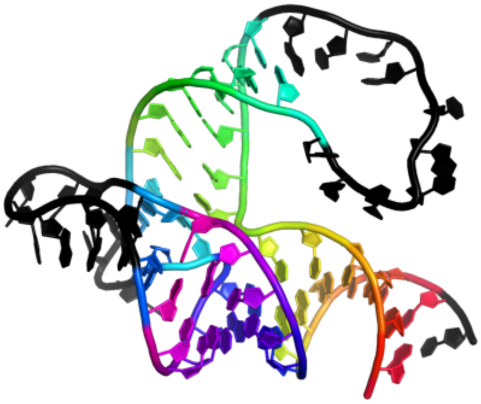

GAACCUCCGCCCAUUGCACUCCGGGCGGUGAAGGAGAGGCGCAAGGUUAACCGCCUCAGGUUCC
((((((((((((..........))))))(<...<)((((((>........>)))))))))))).

1.4 Spinach aptamer

1.4.1 6b14_R

Crystal structure of Spinach RNA aptamer in complex with Fab BL3-6S97N

Release date: 2017-12-27
Method: x-ray diffraction
Resolution: 1.64 Å
Chain length: 83
Extracted length: 83

Total clashes: 0
Clashes per residue: 0.000
Clashscore: 2.380

Description: RNA (86-MER)
Organism: synthetic construct

Koirala, D., Shelke, S.A., Dupont, M., Ruiz, S., DasGupta, S., Bailey, L.J., Benner, S.A., Piccirilli, J.A. (2018) Affinity maturation of a portable Fab-RNA module for chaperone-assisted RNA crystallography. Nucleic Acids Res.
DOI: 10.1093/nar/gkx1292

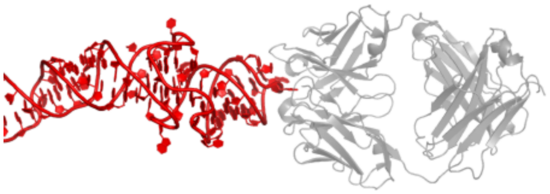

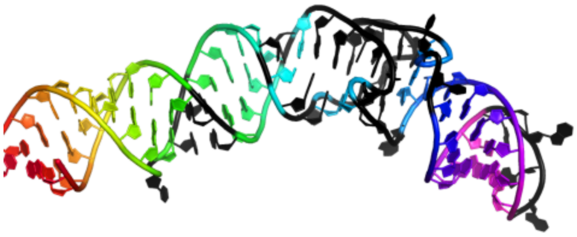

GACGCGACCGAAAUGGUGAAGGACGGGUCCAGUGCGAAACACGCACUGUUGAGUAGAGUGUGAGCUCCGUAACUGGUCGCGUC
((((((((((..((((.(.....(...(((((((((.....)))))))))..)).........)..).))))..).)))))))))

1.4.2 6b3k_R

Crystal structure of mutant Spinach RNA aptamer in complex with Fab BL3-6

Release date: 2017-12-27
Method: x-ray diffraction
Resolution: 2.09 Å
Chain length: 83
Extracted length: 83

Total clashes: 0
Clashes per residue: 0.000
Clashscore: 4.330

Description: RNA (83-MER),RNA (83-MER)
Organism: synthetic construct

Koirala, D., Shelke, S.A., Dupont, M., Ruiz, S., DasGupta, S., Bailey, L.J., Benner, S.A., Piccirilli, J.A. (2018) Affinity maturation of a portable Fab-RNA module for chaperone-assisted RNA crystallography. Nucleic Acids Res.
DOI: 10.1093/nar/gkx1292

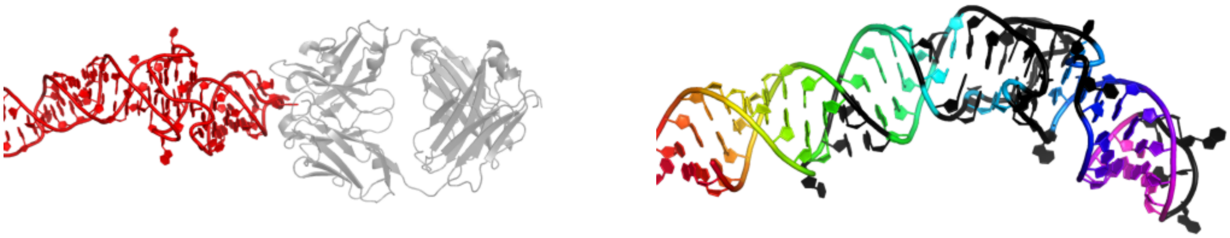

```
GACGCGACCGAAAUGGUGAAGGACGGGUCCAGUGCGAGACCCGCACUGUUGAGUAGAGUGUGAGCUCCGUAACUGGUCGCGUC
((((((((((..((((.(.....(...(((((((((.....)))))))..)).........)..).))))..).)))))))))
```

1.5 Mango aptamer

1.5.1 8u5t_A

Structure of Mango II variant aptamer bound to T01-6A-B

Release date: 2024-03-27
Method: x-ray diffraction
Resolution: 2.20 Å
Chain length: 36
Extracted length: 36

Total clashes: 0
Clashes per residue: 0.000
Clashscore: 2.450

Description: Mango II variant
Organism: synthetic construct

Passalacqua, L.F.M., Ferre-D'Amare, A.R. (N/A) Structure of Mango II variant aptamer bound to T01-6A-B. To Be Published.
DOI: nan

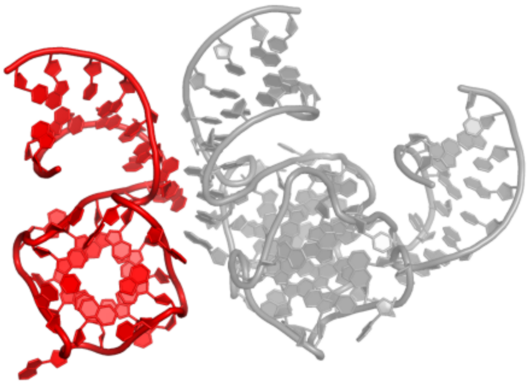

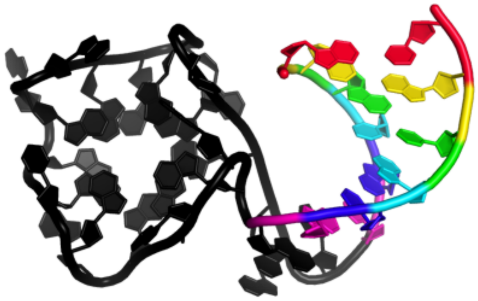

```
GCGUACGAAGGUGAGGAGAGGCGAGGAAGAGUACGC
((((((........................))))))
```

1.5.2 6e8u_B

Structure of the Mango-III (A10U) aptamer bound to TO1-Biotin

Release date: 2019-04-17
Method: x-ray diffraction
Resolution: 1.55 Å
Chain length: 37
Extracted length: 37

Total clashes: 0
Clashes per residue: 0.000
Clashscore: 4.540

Description: RNA (37-MER)
Organism: synthetic construct

Trachman 3rd., R.J., Autour, A., Jeng, S.C.Y., Abdolahzadeh, A., Andreoni, A., Cojocaru, R., Garipov, R., Dolgosheina, E.V., Knutson, J.R., Ryckelynck, M., Unrau, P.J., Ferre-D'Amare, A.R. (2019) Structure and functional reselection of the Mango-III fluorogenic RNA aptamer. Nat. Chem. Biol.
DOI: 10.1038/s41589-019-0267-9

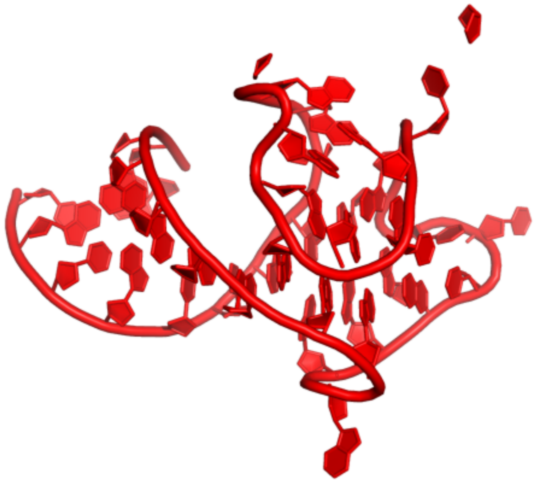
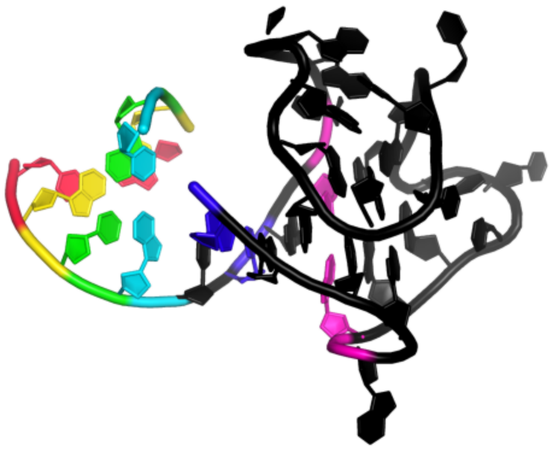

GUACGAAGGAAGGUUUGGUAUGGGGUAGUUGUCGUAC
((((.(....(..................).).))))

1.5.3 6e8t_D

Structure of the Mango-III (A10U) aptamer bound to TO1-Biotin

Release date: 2019-04-17
Method: x-ray diffraction
Resolution: 2.90 Å
Chain length: 36
Extracted length: 36

Total clashes: 31
Clashes per residue: 0.861
Clashscore: 12.160

Description: RNA (35-MER)
Organism: synthetic construct

Trachman 3rd., R.J., Autour, A., Jeng, S.C.Y., Abdolahzadeh, A., Andreoni, A., Cojocaru, R., Garipov, R., Dolgosheina, E.V., Knutson, J.R., Ryckelynck, M., Unrau, P.J., Ferre-D'Amare, A.R. (2019) Structure and functional reselection of the Mango-III fluorogenic RNA aptamer. Nat. Chem. Biol.
DOI: 10.1038/s41589-019-0267-9

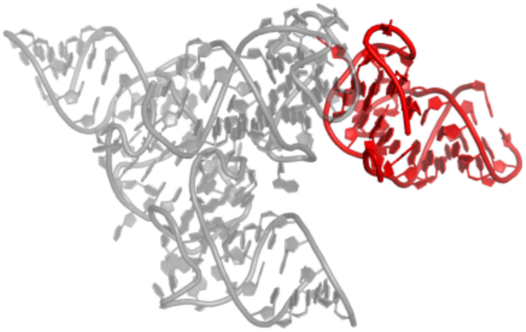

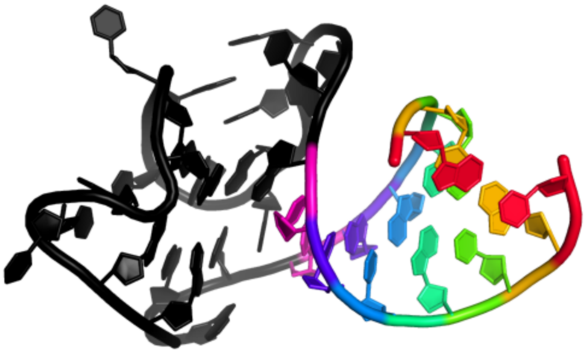

GUACGAAGGAAGGUUUGGUAUGUGGUAUAUUCGUAC
((((((.......................))))))

1.5.4 8u5j_A

Structure of Mango III variant aptamer bound to T01-07M-B

Release date: 2024-03-27
Method: x-ray diffraction
Resolution: 1.70 Å
Chain length: 36
Extracted length: 36

Total clashes: 20
Clashes per residue: 0.556
Clashscore: 7.820

Description: Mango III variant
Organism: synthetic construct

Passalacqua, L.F.M., Ferre-D'Amare, A.R. (N/A) Structure of Mango III variant aptamer bound to T01-07M-B. To Be Published.
DOI: nan

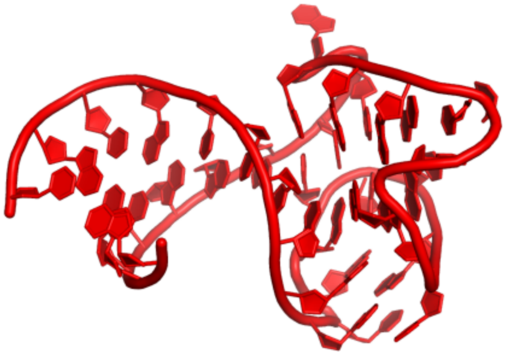

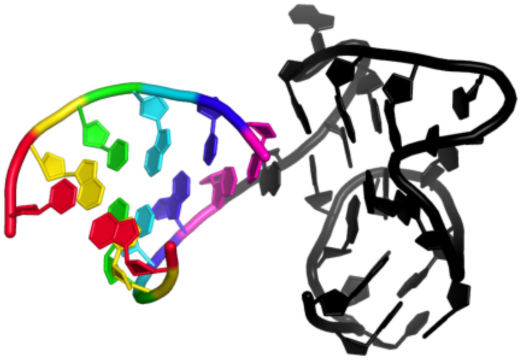

GUACGAAGGAAGGUUUGGUAUGUGGUAGAUUCGUAC
((((((........................))))))

1.5.5 6c65_B

Crystal Structure of the Mango-II-A22U Fluorescent Aptamer Bound to TO1-Biotin

Release date: 2018-08-08
Method: x-ray diffraction
Resolution: 2.80 Å
Chain length: 36
Extracted length: 36

Total clashes: 41
Clashes per residue: 1.139
Clashscore: 23.160

Description: RNA (36-MER)
Organism: synthetic construct

Trachman 3rd., R.J., Abdolahzadeh, A., Andreoni, A., Cojocaru, R., Knutson, J.R., Ryckelynck, M., Unrau, P.J., Ferre-D'Amare, A.R. (2018) Crystal Structures of the Mango-II RNA Aptamer Reveal Heterogeneous Fluorophore Binding and Guide Engineering of Variants with Improved Selectivity and Brightness. Biochemistry.
DOI: 10.1021/acs.biochem.8b00399

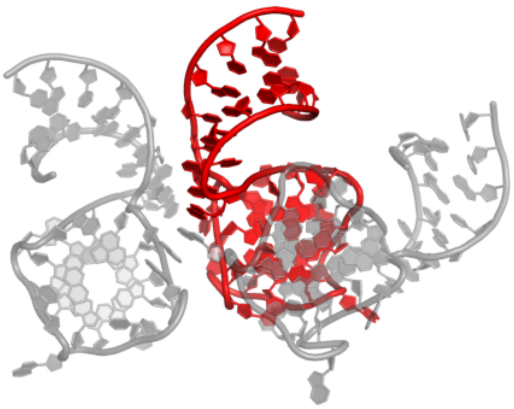

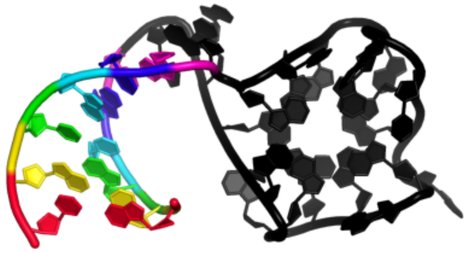

```
GCGUACGAAGGAGAGGAGAGGUAGAGGAGAGUACGC
((((((........................))))))
```

1.5.6 6e8s_B

Structure of the iMango-III aptamer bound to TO1-Biotin

Release date: 2019-04-17
Method: x-ray diffraction
Resolution: 2.35 Å
Chain length: 38
Extracted length: 38

Total clashes: 34
Clashes per residue: 0.895
Clashscore: 12.660

Description: iMango-III aptamer
Organism: synthetic construct

Trachman 3rd., R.J., Autour, A., Jeng, S.C.Y., Abdolahzadeh, A., Andreoni, A., Cojocaru, R., Garipov, R., Dolgosheina, E.V., Knutson, J.R., Ryckelynck, M., Unrau, P.J., Ferre-D'Amare, A.R. (2019) Structure and functional reselection of the Mango-III fluorogenic RNA aptamer. Nat. Chem. Biol.
DOI: 10.1038/s41589-019-0267-9

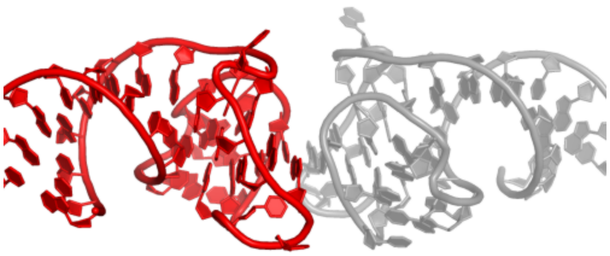

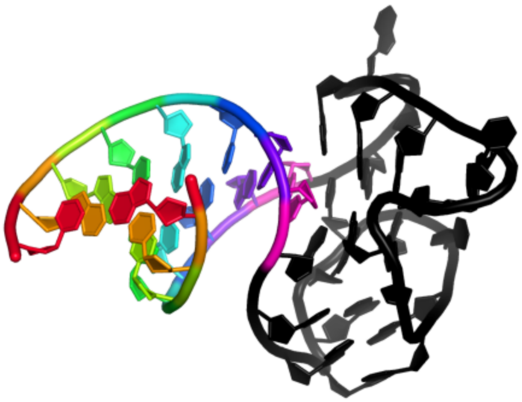

GCUACGAAGGAAGGAUUGGUAUGUGGUAUAUUCGUAGC
((((((((......................))))))))

1.6 Vitamin B12 aptamer

1.6.1 1et4_E

CRYSTAL STRUCTURE OF A VITAMIN B12 BINDING RNA APTAMER WITH LIGAND AT 2.3 A

Release date: 2000-11-13
Method: x-ray diffraction
Resolution: 2.30 Å
Chain length: 35
Extracted length: 35

Total clashes: 24
Clashes per residue: 0.686
Clashscore: 10.070

Description: RNA APTAMER, 35-MER
Organism: nan

Sussman, D., Wilson, C. (2000) A water channel in the core of the vitamin B(12) RNA aptamer. Structure Fold.Des.
DOI: 10.1016/S0969-2126(00)00159-3

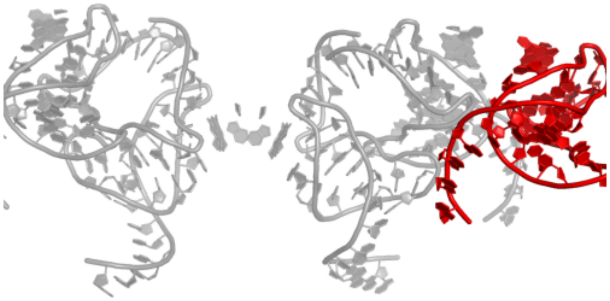

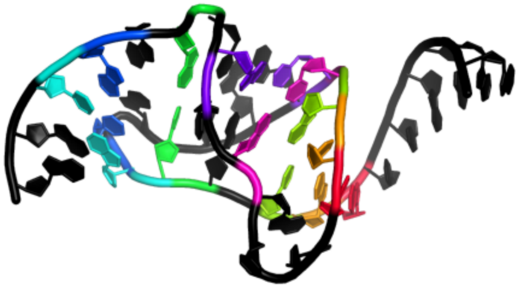

GGAACCGGUGCGCAUAACCACCUCAGUGCGAGCAA
......<<<.((....((>>>...).)..))..

1.7 Squash aptamer

1.7.1 7kvu_G

Crystal structure of Squash RNA aptamer in complex with DFHBI-1T

Release date: 2022-01-19
Method: x-ray diffraction
Resolution: 2.68 Å
Chain length: 83
Extracted length: 83

Total clashes: 0
Clashes per residue: 0.000
Clashscore: 3.680

Description: Squash RNA aptamer
Organism: synthetic construct

Truong, L., Kooshapur, H., Dey, S.K., Li, X., Tjandra, N., Jaffrey, S.R., Ferre-D'Amare, A.R. (2022) The fluorescent aptamer Squash extensively repurposes the adenine riboswitch fold. Nat.Chem.Biol.
DOI: 10.1038/s41589-021-00931-2

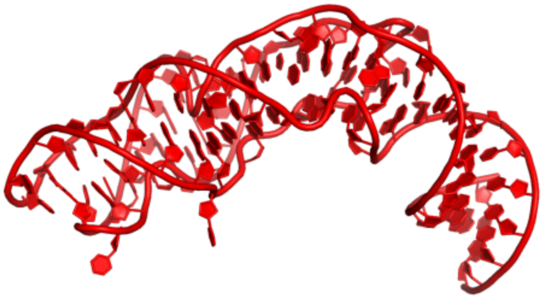

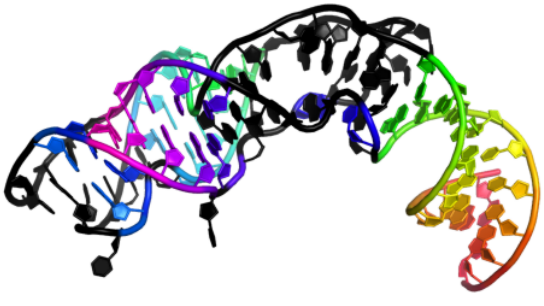

GGGAAGAUACAAGGUGAGCCCAAUAAUAUGGUUUGGGUUAGGAUAGGAAGUAGAGCCUUAAACUCUCUAAGCGGUAUCUUCCC
((((((((((.......((((((......<<.))))))........(..(.((((>>.....)))))....).))))))))))

1.8 Pepper aptamer

1.8.1 7eoh_A

Crystal structure of the Pepper aptamer in complex with HBC

Release date: 2021-11-24
Method: x-ray diffraction
Resolution: 1.64 Å
Chain length: 49
Extracted length: 49

Total clashes: 0
Clashes per residue: 0.000
Clashscore: 0.620

Description: Pepper (49-MER)
Organism: synthetic construct

Huang, K., Chen, X., Li, C., Song, Q., Li, H., Zhu, L., Yang, Y., Ren, A. (2021) Structure-based investigation of fluorogenic Pepper aptamer. Nat.Chem.Biol.
DOI: 10.1038/s41589-021-00884-6

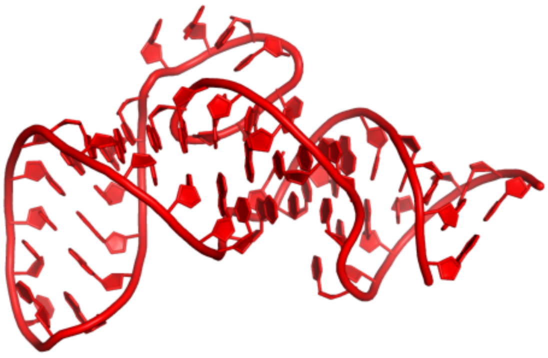

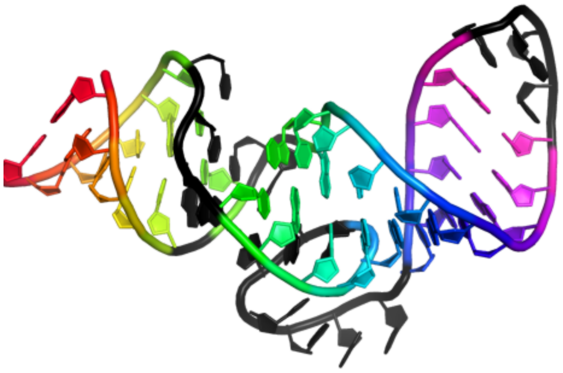

```
GGCGCACUGGCGCUGCGCCUUCGGGCGCCAAUCGUAGCGUGUCGGCGCC
(((((.(..((((((((((....))))).......)))))...))))))
```

1.9 Chili aptamer

1.9.1 7oax_D

Crystal structure of the Chili RNA aptamer in complex with DMHBO+

Release date: 2021-06-16
Method: x-ray diffraction
Resolution: 2.24 Å
Chain length: 52
Extracted length: 52

Total clashes: 4
Clashes per residue: 0.077
Clashscore: 1.550

Description: Chili RNA Aptamer
Organism: synthetic construct

Mieczkowski, M., Steinmetzger, C., Bessi, I., Lenz, A.K., Schmiedel, A., Holzapfel, M., Lambert, C., Pena, V., Hobartner, C. (2021) Large Stokes shift fluorescence activation in an RNA aptamer by intermolecular proton transfer to guanine. Nat Commun.
DOI: 10.1038/s41467-021-23932-0

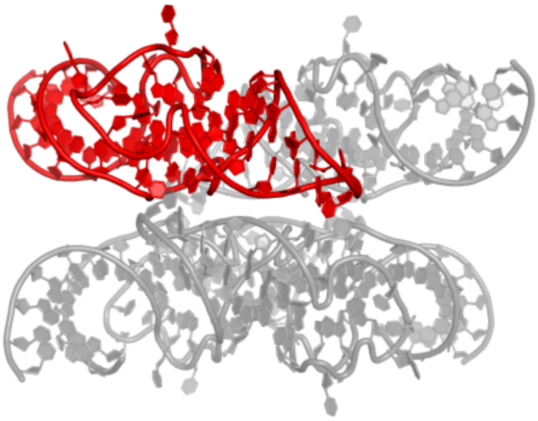

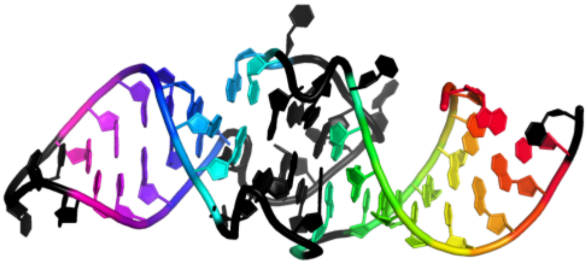

GGCUAGCUGGAGGGGCGCCAGUUCGCUGGUGGUUGGGUGCGGUCGGCUAGCC
.(((((((.(....(((((((....))))).........))...)))))))).

1.10 Corn aptamer

1.10.1 5bjo_Y

Crystal structure of the Corn RNA aptamer in complex with DFHO, site-specific 5-iodo-U

Release date: 2017-09-27
Method: x-ray diffraction
Resolution: 2.35 Å
Chain length: 36
Extracted length: 36

Total clashes: 0
Clashes per residue: 0.000
Clashscore: 4.190

Description: RNA (36-MER)
Organism: synthetic construct

Warner, K.D., Sjekloca, L., Song, W., Filonov, G.S., Jaffrey, S.R., Ferre-D'Amare, A.R. (2017) A homodimer interface without base pairs in an RNA mimic of red fluorescent protein. Nat. Chem. Biol.
DOI: 10.1038/nchembio.2475

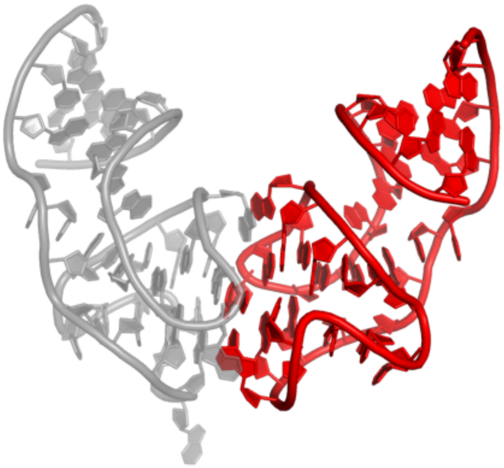

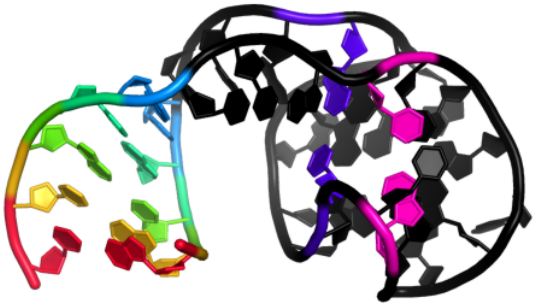

```
GGCGCGAGGAAGGAGGUCUGAGGAGGUCACUGCGCC
(((((...(........).(.......)...)))))
```

1.11 DIR2s aptamer

1.11.1 6db8_R

Structural basis for promiscuous binding and activation of fluorogenic dyes by DIR2s RNA aptamer

Release date: 2018-11-14
Method: x-ray diffraction
Resolution: 1.87 Å
Chain length: 60
Extracted length: 60

Total clashes: 0
Clashes per residue: 0.000
Clashscore: 4.480

Description: RNA (60-MER)
Organism: synthetic construct

Shelke, S.A., Shao, Y., Laski, A., Koirala, D., Weissman, B.P., Fuller, J.R., Tan, X., Constantin, T.P., Waggoner, A.S., Bruchez, M.P., Armitage, B.A., Piccirilli, J.A. (2018) Structural basis for activation of fluorogenic dyes by an RNA aptamer lacking a G-quadruplex motif. Nat Commun.
DOI: 10.1038/s41467-018-06942-3

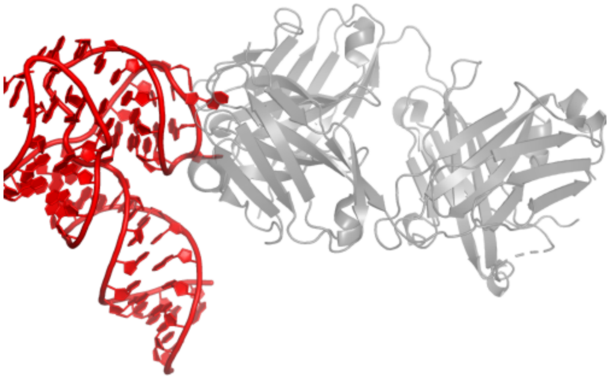

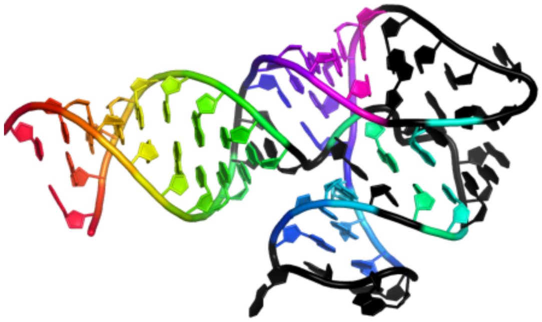

```
GGAUGCGCCUUGAAAAGCCUGCGAAACACGCAGCUGGUGAAUGACAGCUAUGGCGCAUCC
(((((((((..(.....<).(((.....)))(((((......>.)))))..)))))))))
```

1.12 Clivia aptamer

1.12.1 8hze_B

A new fluorescent RNA aptamer bound with N

Release date: 2024-06-19
Method: x-ray diffraction
Resolution: 1.59 Å
Chain length: 35
Extracted length: 35

Total clashes: 0
Clashes per residue: 0.000
Clashscore: 2.480

Description: RNA (36-MER)
Organism: synthetic construct

Huang, K., Song, Q., Fang, M., Yao, D., Shen, X., Xu, X., Chen, X., Zhu, L., Yang, Y., Ren, A. (2024) Structural basis of a small monomeric Clivia fluorogenic RNA with a large Stokes shift. Nat.Chem.Biol.
DOI: 10.1038/s41589-024-01633-1

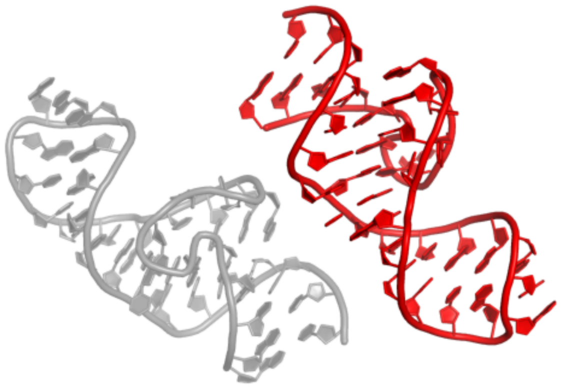

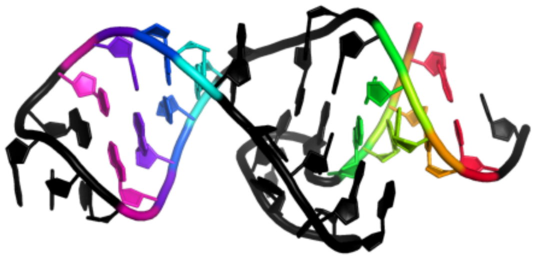

```
GAAGAUUGUAAACAUGCCGAAAGGCAGACACUUCC
((((..........((((....))))....)))).
```

1.12.2 8hzj_A

A new fluorescent RNA aptamer bound with N571

Release date: 2024-06-19
Method: x-ray diffraction
Resolution: 2.60 Å
Chain length: 35
Extracted length: 35

Total clashes: 2
Clashes per residue: 0.057
Clashscore: 2.500

Description: RNA (36-MER)
Organism: synthetic construct

Huang, K., Song, Q., Fang, M., Yao, D., Shen, X., Xu, X., Chen, X., Zhu, L., Yang, Y., Ren, A. (2024) Structural basis of a small monomeric Clivia fluorogenic RNA with a large Stokes shift. Nat.Chem.Biol.
DOI: 10.1038/s41589-024-01633-1

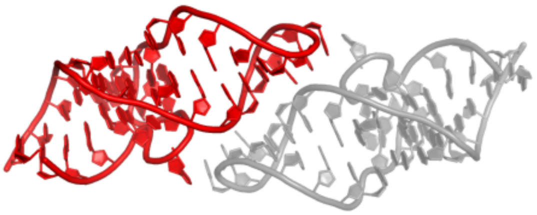

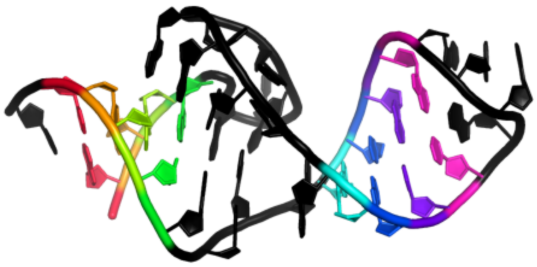

```
GAAGAUUGUAAACAUGCCGAAAGGCAGACACUUCC
((((..........((((....))))....)))).
```

1.12.3 8hzl_B

A new fluorescent RNA aptamer_III bound with N

Release date: 2024-06-19
Method: x-ray diffraction
Resolution: 2.60 Å
Chain length: 84
Extracted length: 84

Total clashes: 26
Clashes per residue: 0.310
Clashscore: 5.170

Description: RNA (84-MER)
Organism: synthetic construct

Huang, K., Song, Q., Fang, M., Yao, D., Shen, X., Xu, X., Chen, X., Zhu, L., Yang, Y., Ren, A. (2024) Structural basis of a small monomeric Clivia fluorogenic RNA with a large Stokes shift. Nat.Chem.Biol.
DOI: 10.1038/s41589-024-01633-1

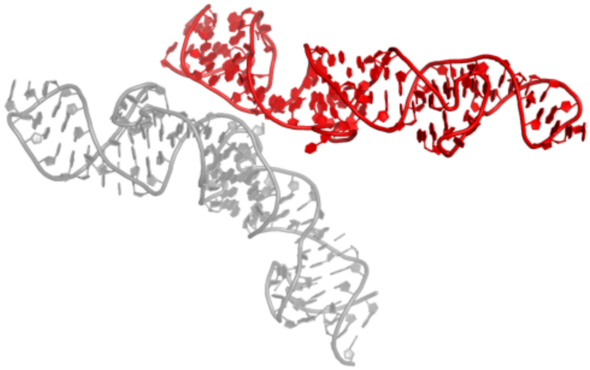

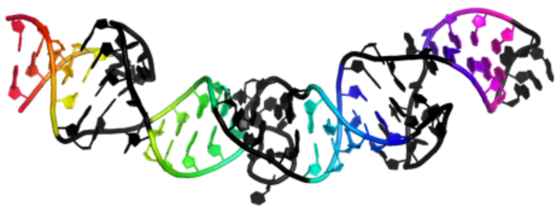

GGAAGAUUGUAAACAGCGAGAUUGUAAACAGCGAGAUUGUAAACAUGCCGAAAGGCAGACACUCGCGACACUCGCGACACUUUC
(((((..........(((((..........(((((..........((((....))))....)))))....)))))....)))))

1.13 A9g aptamer

1.13.1 6rti_X

X-ray structure of human glutamate carboxypeptidase II (GCPII) in complex with aptamer A9g

Release date: 2020-06-10
Method: x-ray diffraction
Resolution: 2.20 Å
Chain length: 43
Extracted length: 43

Total clashes: 0
Clashes per residue: 0.000
Clashscore: 3.130

Description: Aptamer A9g, RNA (43-MER)
Organism: synthetic construct

Ptacek, J., Zhang, D., Qiu, L., Kruspe, S., Motlova, L., Kolenko, P., Novakova, Z., Shubham, S., Havlinova, B., Baranova, P., Chen, S.J., Zou, X., Giangrande, P., Barinka, C. (2020) Structural basis of prostate-specific membrane antigen recognition by the A9g RNA aptamer. Nucleic Acids Res.
DOI: 10.1093/nar/gkaa494

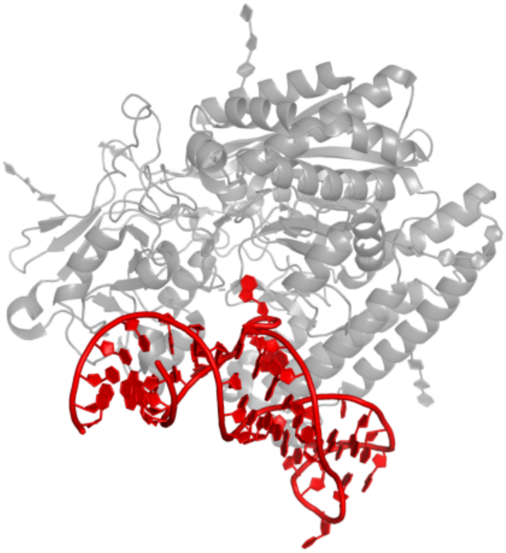

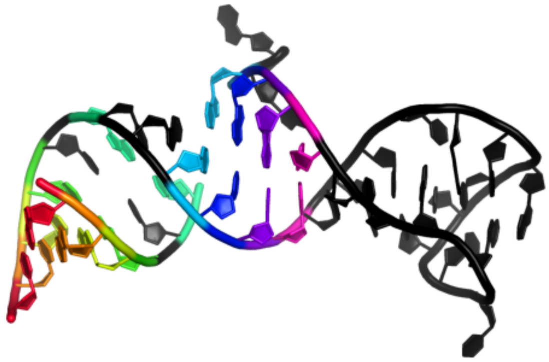

GGGACCGAAAAAGACCUGACUUCUAUACUAAGUCUACGUUCCC
((((.(.....((((...............))))..).))))

1.14 Beetroot aptamer

1.14.1 8eyu_B

Structure of Beetroot dimer bound to DFAME

Release date: 2023-05-31
Method: x-ray diffraction
Resolution: 1.95 Å
Chain length: 49
Extracted length: 49

Total clashes: 5
Clashes per residue: 0.102
Clashscore: 3.240

Description: RNA (49-MER)
Organism: synthetic construct

Passalacqua, L.F.M., Starich, M.R., Link, K.A., Wu, J., Knutson, J.R., Tjandra, N., Jaffrey, S.R., Ferre-D'Amare, A.R. (2023) Co-crystal structures of the fluorogenic aptamer Beetroot show that close homology may not predict similar RNA architecture. Nat Commun.
DOI: 10.1038/s41467-023-38683-3

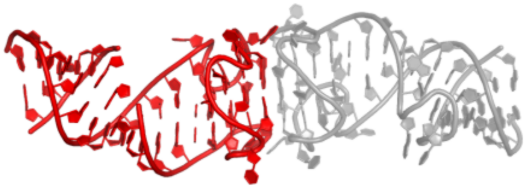

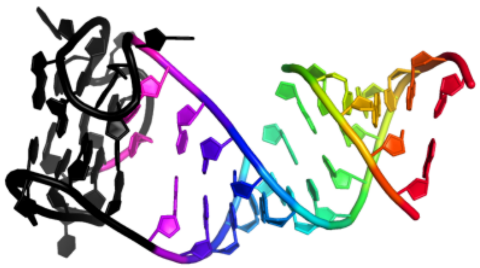

```
GCGCCGGUUAGGCAGAGGUGGGUGGUGUGGAGGAGUAUCUGUCCGGCGC
((((((....((((((.........(..........)))))))))))))
```

1.14.2 8f0n_B

Wobble Beetroot (A16U-U38G) dimer bound to DFHO

Release date: 2023-05-31
Method: x-ray diffraction
Resolution: 2.85 Å
Chain length: 49
Extracted length: 49

Total clashes: 26
Clashes per residue: 0.531
Clashscore: 10.100

Description: RNA (49-MER)
Organism: synthetic construct

Passalacqua, L.F.M., Starich, M.R., Link, K.A., Wu, J., Knutson, J.R., Tjandra, N., Jaffrey, S.R., Ferre-D'Amare, A.R. (2023) Co-crystal structures of the fluorogenic aptamer Beetroot show that close homology may not predict similar RNA architecture. Nat Commun.
DOI: 10.1038/s41467-023-38683-3

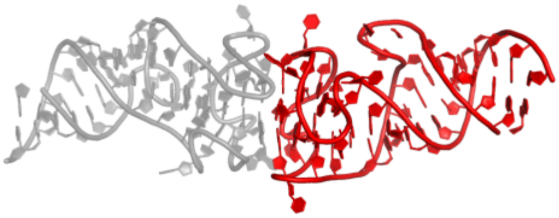

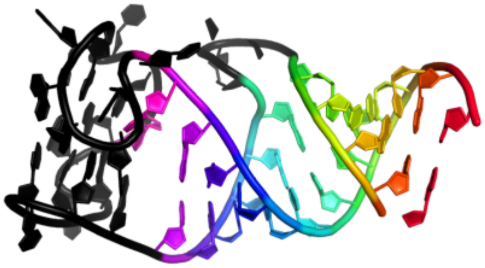

```
GCGCCGGUUAGGCAGUGGUGGGUGGUGUGGAGGAGUAGCUGUCCGGCGC
((((((....((((((.........(..........)))))))))))))
```

1.15 Malachite green aptamer

1.15.1 1f1t_A

CRYSTAL STRUCTURE OF THE MALACHITE GREEN APTAMER COMPLEXED WITH TETRAMETHYL-ROSAMINE

Release date: 2000-09-04
Method: x-ray diffraction
Resolution: 2.80 Å
Chain length: 38
Extracted length: 38

Total clashes: 92
Clashes per residue: 2.421
Clashscore: 35.830

Description: MALACHITE GREEN APTAMER RNA
Organism: nan

Baugh, C., Grate, D., Wilson, C. (2000) 2.8 A crystal structure of the malachite green aptamer. J.Mol.Biol.
DOI: 10.1006/jmbi.2000.3951

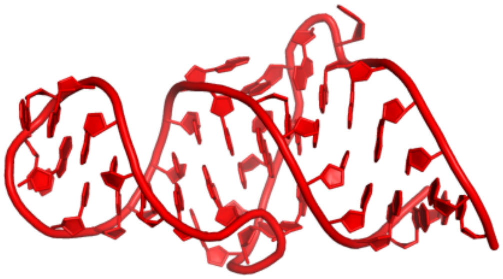

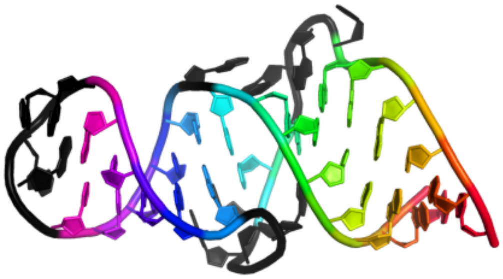

GGAUCCCGACUGGCGAGAGCCAGGUAACGAAUGGAUCC
((((((((.(((((....)))))....))...))))))

1.16 11F7t aptamer

1.16.1 5voe_A

DesGla-XaS195A Bound to Aptamer 11F7t

Release date: 2018-06-20
Method: x-ray diffraction
Resolution: 2.00 Å
Chain length: 36
Extracted length: 36

Total clashes: 0
Clashes per residue: 0.000
Clashscore: 12.260

Description: Aptamer 11F7t (36-MER)
Organism: synthetic construct

Gunaratne, R., Kumar, S., Frederiksen, J.W., Stayrook, S., Lohrmann, J.L., Perry, K., Bompiani, K.M., Chabata, C.V., Thalji, N.K., Ho, M.D., Arepally, G., Camire, R.M., Krishnaswamy, S., Sullenger, B.A. (2018) Combination of aptamer and drug for reversible anticoagulation in cardiopulmonary bypass. Nat. Biotechnol.
DOI: 10.1038/nbt.4153

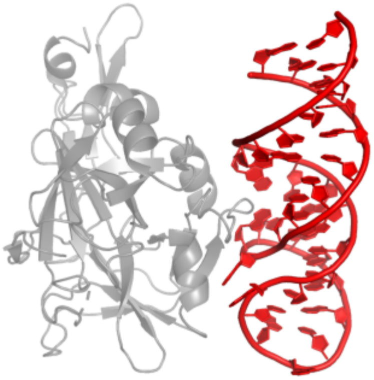

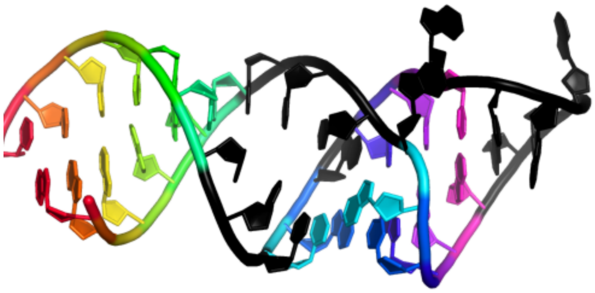

GAGAGCCCCAGCGAGAUAAUACUUGGCCCCGCUCUU
((((((....(((((......))).))...))))))

1.17 K1 aptamer

1.17.1 6sy4_C

TetR in complex with the TetR-binding RNA-aptamer K1

Release date: 2020-02-05
Method: x-ray diffraction
Resolution: 2.69 Å
Chain length: 43
Extracted length: 38

Total clashes: 4
Clashes per residue: 0.093
Clashscore: 3.720

Description: TetR-binding aptamer K1 (43-MER)
Organism: Escherichia coli

Grau, F.C., Jaeger, J., Groher, F., Suess, B., Muller, Y.A. (2020) The complex formed between a synthetic RNA aptamer and the transcription repressor TetR is a structural and functional twin of the operator DNA-TetR regulator complex. Nucleic Acids Res.
DOI: 10.1093/nar/gkaa083

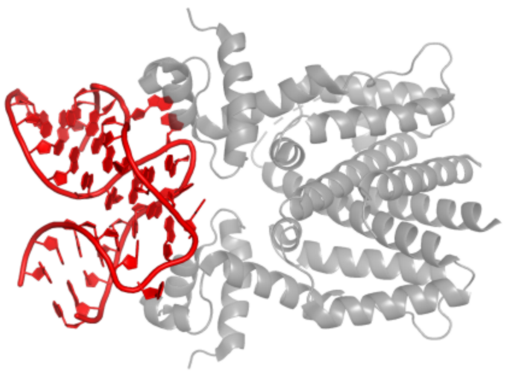

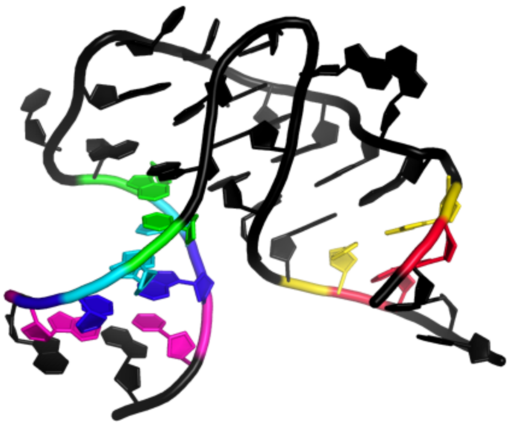

GGCCGGAGAAUGUUAUGGCGCGAAAGCGCAGAGAAAACCGGUC
....((...........((((....))))........))....

1.18 Tetracycline aptamer

1.18.1 3egz_B

Crystal structure of an in vitro evolved tetracycline aptamer and artificial riboswitch

Release date: 2008-10-28
Method: x-ray diffraction
Resolution: 2.20 Å
Chain length: 65
Extracted length: 65

Total clashes: 136
Clashes per residue: 2.092
Clashscore: 24.650

Description: Tetracycline aptamer and artificial riboswitch
Organism: nan

Xiao, H., Edwards, T.E., Ferre-D'Amare, A.R. (2008) Structural basis for specific, high-affinity tetracycline binding by an in vitro evolved aptamer and artificial riboswitch. Chem.Biol.
DOI: 10.1016/j.chembiol.2008.09.004

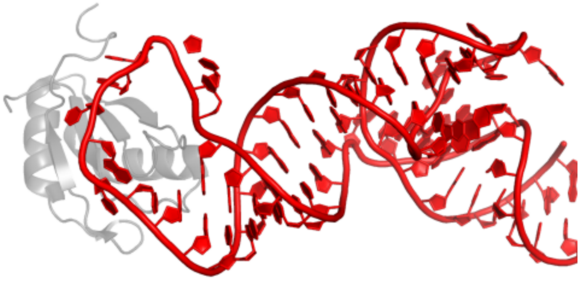

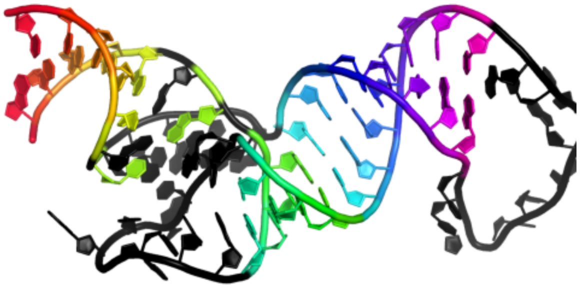

GAGGGAGAGGUGAAGAAUACGACCACCUAGGUACCAUUGCACUCCGGUACCUAAAACAUACCCUC
(((((.(.((((...........))))((((((((..........)))))))).....).)))))

2 CRISPR guides

2.1 Cas9 guide

2.1.1 7el1_B

Structure of a protein from bacteria

Release date: 2021-07-28
Method: x-ray diffraction
Resolution: 2.22 Å
Chain length: 73
Extracted length: 53

Total clashes: 9
Clashes per residue: 0.123
Clashscore: 6.160

Description: RNA (73-MER)
Organism: Staphylococcus aureus

Liu, H., Zhu, Y., Lu, Z., Huang, Z. (2021) Structural basis of Staphylococcus aureus Cas9 inhibition by AcrIIA14. Nucleic Acids Res.
DOI: 10.1093/nar/gkab487

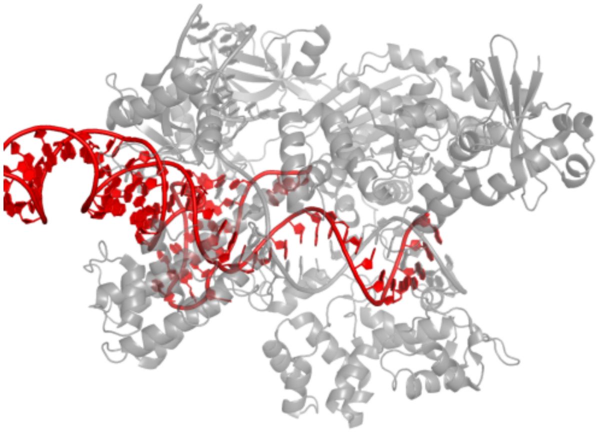

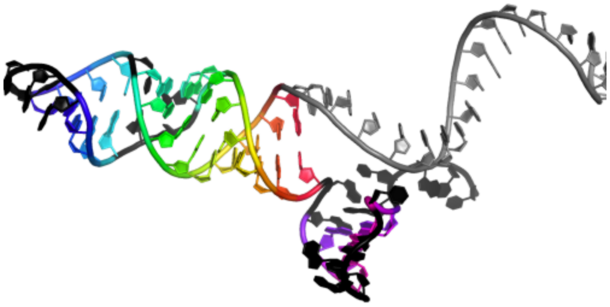

```
GGAAAUUAGGUGCGCUUGGCGUUUUAGUACUCUGGAAACAGAAUCUACUAAAACAAGGCAAAAUGCCGUGUUU
....................(((((((((.((((....))))...)))))))))..(((.....))).......
```

2.1.2 6wbr_B

Crystal structure of AceCas9 bound with guide RNA and DNA with 5'-NNNCC-3' PAM

Release date: 2020-11-18
Method: x-ray diffraction
Resolution: 2.91 Å
Chain length: 94
Extracted length: 74

Total clashes: 73
Clashes per residue: 0.777
Clashscore: 11.650

Description: RNA (94-MER)
Organism: Acidothermus cellulolyticus 11B

Das, A., Hand, T.H., Smith, C.L., Wickline, E., Zawrotny, M., Li, H. (2020) The molecular basis for recognition of 5'-NNNCC-3' PAM and its methylation state by Acidothermus cellulolyticus Cas9. Nat Commun.
DOI: 10.1038/s41467-020-20204-1

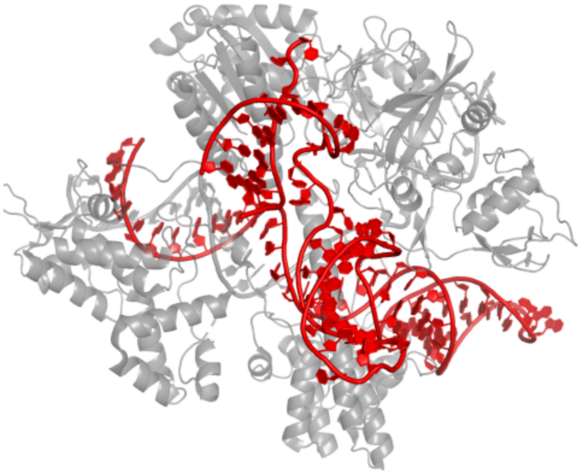

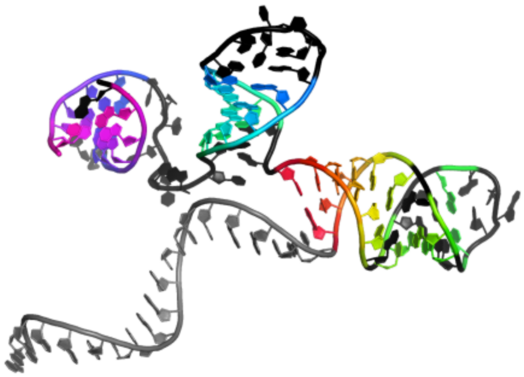

```
GGAUGGCAAGAUCCUGGUAUGCUGGGGAGCCUGAAAAGGCUACCUAGCAAGACCCCUUCGUGGGGUCGCAUUCUUCACCCCCAGCAGGGGGUUC
....................((((((.((..(....)..)).))))))..(((((.......))))).........((((((....))))))..
```

2.1.3 8umf_B

Structure of PsCas9 in complex with gRNA and DNA in product state

Release date: 2024-10-02
Method: electron microscopy
Resolution: 2.90 Å
Chain length: 131
Extracted length: 101

Total clashes: 50
Clashes per residue: 0.382
Clashscore: 4.930

Description: RNA (121-MER)
Organism: synthetic construct

Bravo, J.P.K., Taylor, D.W. (N/A) Structure-guided engineering of PsCas9 yields a high fidelity and activity enzyme for in vivo gene editing. To Be Published.
DOI: nan

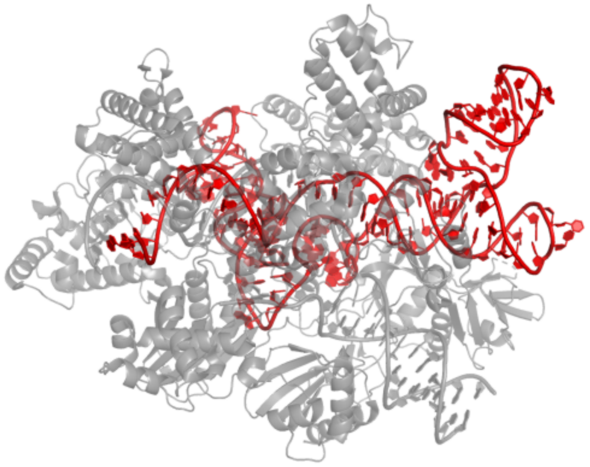

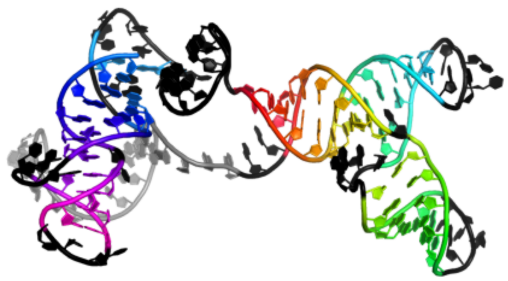

AUGUCACCUCCAAUGACUAGGGGUUUCAGUUAUUCGUGAAAACGAAUGAAGUCACUCUUAAAGUGAGCUGAAAUCACUAAAAAUUAAGAUUGAACCCGGCUACUGACUCUGUCAUCCGGGUUUACUUAUUU

......................((((((((.((((((....))))))....(((((.....))))))))))))).........!!..(....)..((((((....((((...)))).))))))..........

2.1.4 8hud_B

Cryo-EM structure of the EvCas9-sgRNA-target DNA ternary complex

Release date: 2023-12-27
Method: electron microscopy
Resolution: 3.43 Å
Chain length: 75
Extracted length: 52

Total clashes: 19
Clashes per residue: 0.253
Clashscore: 10.270

Description: sgRNA
Organism: synthetic construct

Tang, N., Wu, Z., Gao, Y., Chen, W., Wang, Z., Su, M., Ji, W., Ji, Q. (2024) Molecular Basis and Genome Editing Applications of a Compact Eubacterium ventriosum CRISPR-Cas9 System. Acs Synth Biol.
DOI: 10.1021/acssynbio.3c00501

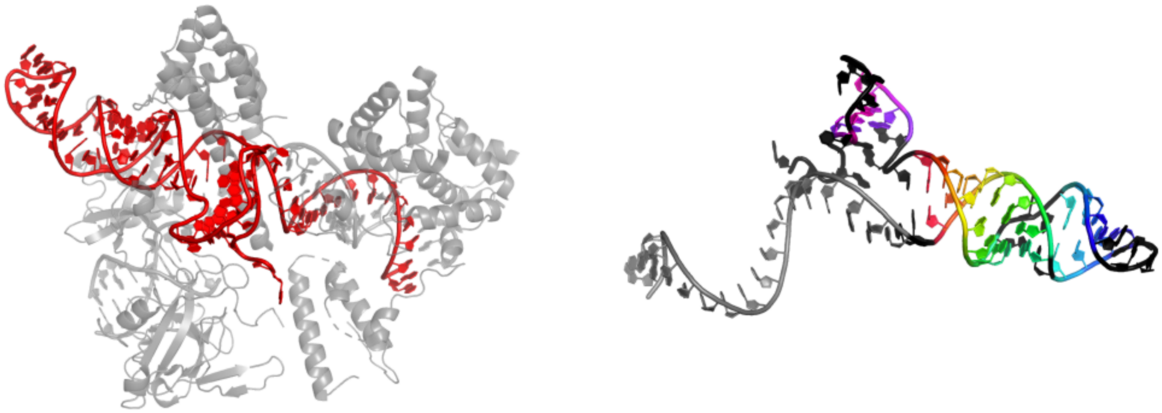

GGUAAUCGCUCUCCUCCGGC**GAUUUUAGUACCUGAGAAAUCAGAUCUACUAAAACAAGGCUUUAUGCCGAAA**UCA
......................((((((((.((((....))))...))))))))...(((.....))).......

2.2 sgRNA guide

2.2.1 8rdu_1

Conformational Landscape of the Type V-K CRISPR-associated TransposonIntegration Assembly CAST V-K composite map

Release date: 2024-06-19
Method: electron microscopy
Resolution: 2.30 Å
Chain length: 261
Extracted length: 227

Total clashes: 16
Clashes per residue: 0.061
Clashscore: 3.440

Description: sgRNA
Organism: Scytonema hofmannii

Tenjo-Castano, F., Sofos, N., Stutzke, L.S., Temperini, P., Fuglsang, A., Pape, T., Mesa, P., Montoya, G. (2024) Conformational landscape of the type V-K CRISPR-associated transposon integration assembly. Mol.Cell.
DOI: 10.1016/j.molcel.2024.05.005

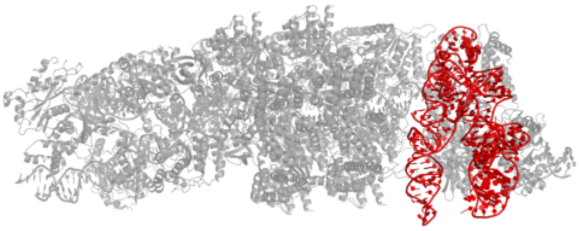

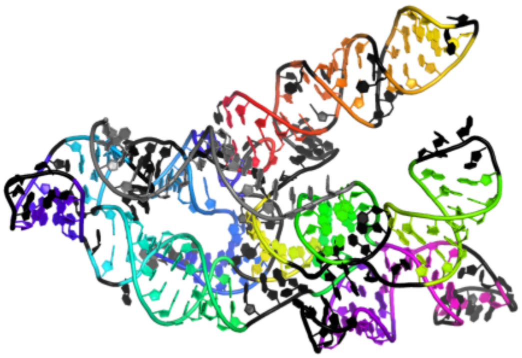

GGAUAUUAAUAGCGCCGCAAUUCAUGCUGCUUGCAGCCUCUGAAUUUUGUUAAAUGAGGGUUAGUUUGACUGUAUAAAUACAGUCUUGCUUUCUGACCCUGGUAGCUGCUCACCCUGAUGCUGCUGUCAAUAGACAGGAUAGGUGCGCUCCCAGCAAUAAGGGCGCGGAUGUACUGCUGUAGUGGCUACUGAAUCACCCCCGAUCAAGGGGGAACCCUCCAAAAGGUGGGUUGAAAGGAGAAGUCAUUUAAUAAGGCCACU

...........<<<<((.(.(((..(((((..)))))....))).).))........<<<<......(((((((....)))))))...(((((((>>>>.(((((((((((.(((....(((((((((....)))))....((.>>>>.)))))).....))).((((.....)))).).))))))))))....))(((((......)))))(((((..(....)..))))))))))........................

2.2.2 8x5v_B

BlCas9-sgRNA-target DNA complex

Release date: 2024-07-10
Method: x-ray diffraction
Resolution: 2.00 Å
Chain length: 110
Extracted length: 91

Total clashes: 2
Clashes per residue: 0.018
Clashscore: 0.940

Description: RNA (110-mer)
Organism: Brevibacillus laterosporus

Nakane, T., Nakagawa, R., Ishiguro, S., Okazaki, S., Mori, H., Shuto, Y., Yamashita, K., Yachie, N., Nishimasu, H., Nureki, O. (2024) Structure and engineering of Brevibacillus laterosporus Cas9. Commun Biol.
DOI: 10.1038/s42003-024-06422-z

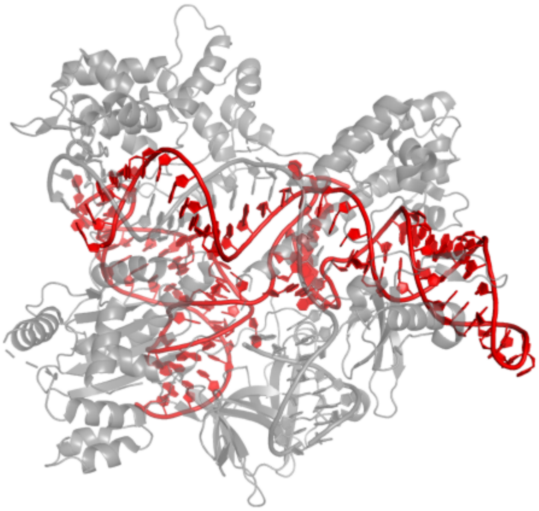

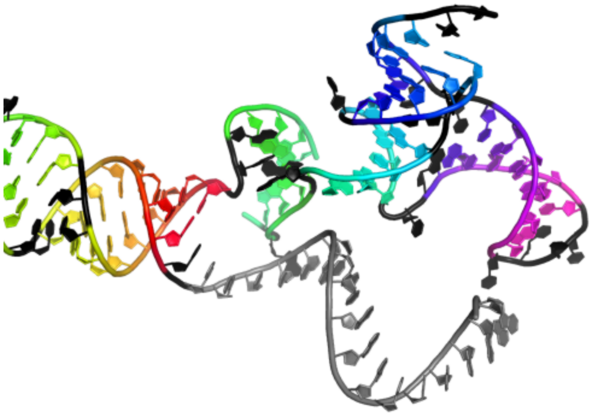

GGAAAUUAGGUGCGCUUGGCGCUAUAGUUCCUUGAAAAAGUUGCUAUAGUAAGGGCAACAGACCCGAGGCGUUGGGGAUCGCCUAGCCCGUUUUUACGGGCUCUCCCCAU
....................((((((((..(((....)))..))))))))..((((....).)))..<<<<..(((((.>>>>..((((((....))))))..)))))..

2.2.3 7c7l_C

Cryo-EM structure of the Cas12f1-sgRNA-target DNA complex

Release date: 2020-12-23
Method: electron microscopy
Resolution: 3.30 Å
Chain length: 180
Extracted length: 110

Total clashes: 29
Clashes per residue: 0.161
Clashscore: 4.790

Description: sgRNA
Organism: uncultured archaeon

Takeda, S.N., Nakagawa, R., Okazaki, S., Hirano, H., Kobayashi, K., Kusakizako, T., Nishizawa, T., Yamashita, K., Nishimasu, H., Nureki, O. (2021) Structure of the miniature type V-F CRISPR-Cas effector enzyme. Mol.Cell.
DOI: 10.1016/j.molcel.2020.11.035

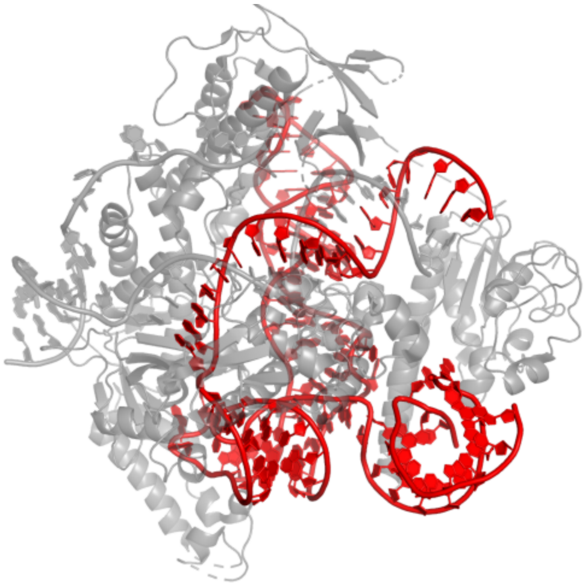 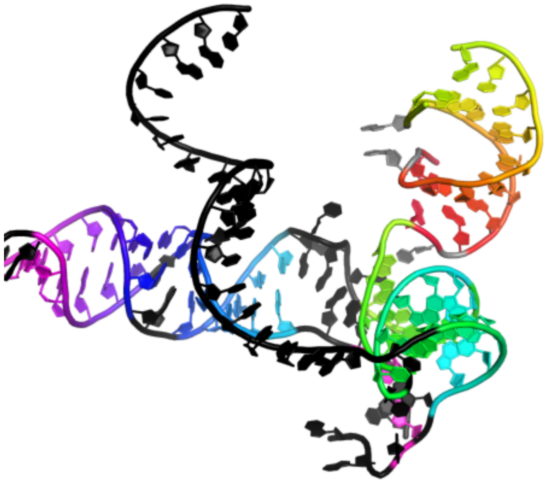

UUCACUGAUAAAGUGGAGAACCGCUUCACCAAAAGCUGUCCCUUAGGGGAUUAGAACUUGAGUGAAGGUGGGCUGCUUGCAUCAGCCUAAUGUCGAGAAGUGCUUUCUUCGGAAAGUAACCCUCGAAACAAAUUCAUUUGAAAGAAUGAAGGAAUGCAACGGAAAUUAGGUGCGCUUGGC

....................((.((((((((...........................)).))))))).)<<<<<.((((((>>>>>.....(((((..((((((((....)))))).)).)))))..(......................).))))))).....................

2.2.4 5wti_B

Crystal structure of the CRISPR-associated protein in complex with crRNA and DNA

Release date: 2017-11-01
Method: x-ray diffraction
Resolution: 2.68 Å
Chain length: 123
Extracted length: 104

Total clashes: 0
Clashes per residue: 0.000
Clashscore: 7.540

Description: RNA (123-MER)
Organism: RNA transcription vector pBRDI1

Wu, D., Guan, X., Zhu, Y., Ren, K., Huang, Z. (2017) Structural basis of stringent PAM recognition by CRISPR-C2c1 in complex with sgRNA. Cell Res.
DOI: 10.1038/cr.2017.46

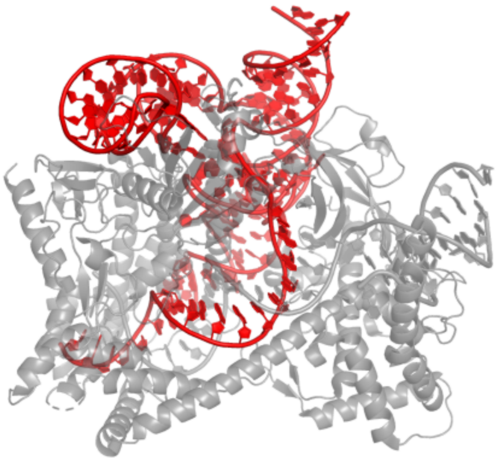

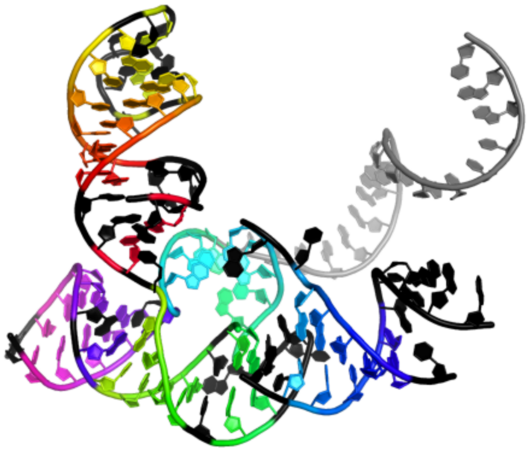

GGCGAGGUUCUGUCUUUUGGUCAGGACAACCGUCUAGCUAUAAGUGCUGCAGGGGUGUGAGAAACUCCUAUUGCUGGACGAUGUCUCUUUCGAGGCAUUAGCACCGGGGAGAAGUCAUUUAAU

.(....((((((.(.....).))))))..).((((.((.....<<<<<..(((((.........)))))...)).))))(.(((((......))))).)>>>>>...................

2.3 Cas12 guide

2.3.1 8bf8_B

ISDra2 TnpB in complex with reRNA

Release date: 2023-04-12
Method: electron microscopy
Resolution: 2.80 Å
Chain length: 150
Extracted length: 123

Total clashes: 49
Clashes per residue: 0.327
Clashscore: 8.730

Description: Deinococcus radiodurans R1 chromosome 1
Organism: Deinococcus radiodurans R1 = ATCC 13939 = DSM 20539

Sasnauskas, G., Tamulaitiene, G., Druteika, G., Carabias, A., Silanskas, A., Kazlauskas, D., Venclovas, C., Montoya, G., Karvelis, T., Siksnys, V. (2023) TnpB structure reveals minimal functional core of Cas12 nuclease family. Nature.
DOI: 10.1038/s41586-023-05826-x

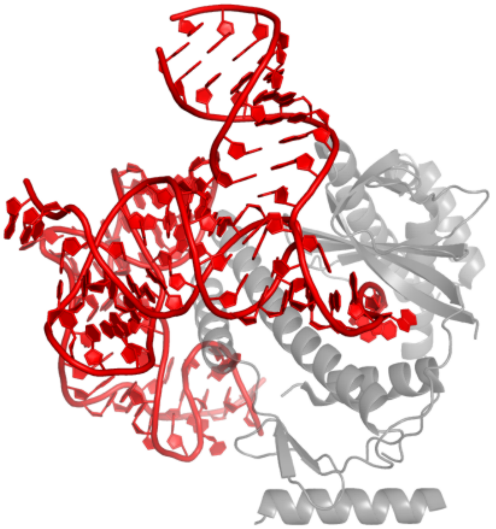

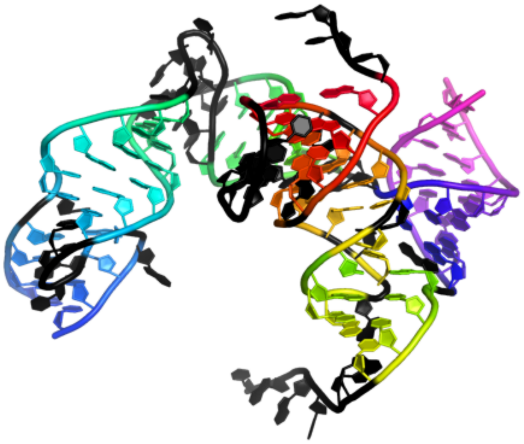

CAUUCGGCGUGAAGCGUUGGUGGCUGCGGGAAUCUCAGACACCUUAAACGCUCAUGGAGGCUAUGUCAGACCUGCUUCGGCGGGCAAUGGUCUGCGAAGUGAGAAUCACGCGACUUUAGUCGUGUGAGGUUCAAGAGUCCCUUGGCGCCC

..................((((.(((.(.<<<<).))).)))).......(((((...((((((....(.(((........)))).))))))......)))))..(((((((((....))))))))).>>>>..................

2.3.2 8j3r_C

Cryo-EM structure of the AsCas12f-HKRA-sgRNAS3-5v7-target DNA

Release date: 2023-09-27
Method: electron microscopy
Resolution: 2.95 Å
Chain length: 118
Extracted length: 109

Total clashes: 18
Clashes per residue: 0.153
Clashscore: 5.620

Description: RNA (118-MER)
Organism: Sulfoacidibacillus thermotolerans

Hino, T., Omura, S.N., Nakagawa, R., Togashi, T., Takeda, S.N., Hiramoto, T., Tasaka, S., Hirano, H., Tokuyama, T., Uosaki, H., Ishiguro, S., Kagieva, M., Yamano, H., Ozaki, Y., Motooka, D., Mori, H., Kirita, Y., Kise, Y., Itoh, Y., Matoba, S., Aburatani, H., Yachie, N., Karvelis, T., Siksnys, V., Ohmori, T., Hoshino, A., Nureki, O. (2023) An AsCas12f-based compact genome-editing tool derived by deep mutational scanning and structural analysis. Cell.
DOI: 10.1016/j.cell.2023.08.031

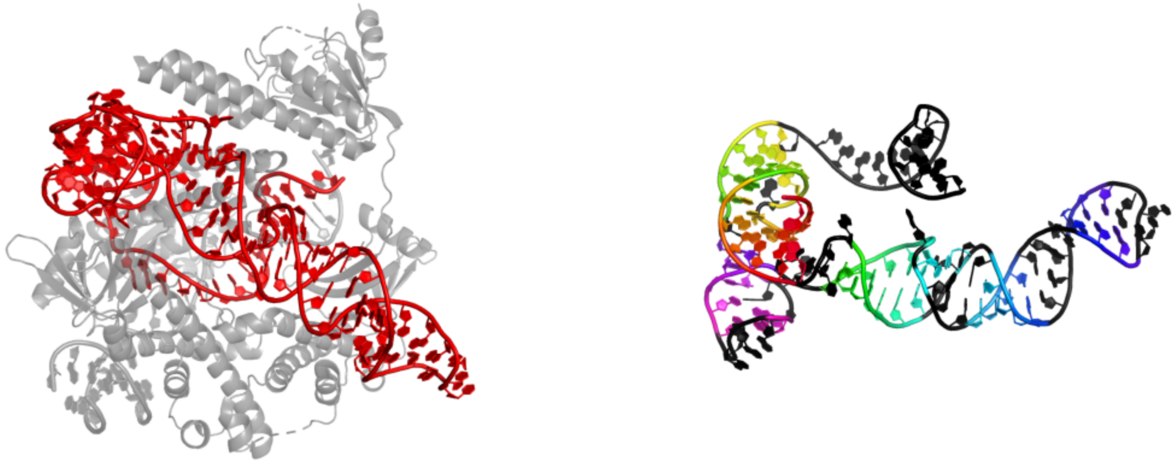

GGAUUCGUCGGUUCAGCGACGAUAAGCCGAGAAGUGCCAAUAAAACUGUUAAGUGGUUUGGUAACGCUCGGUAAGGUCCGAAAGGAGAACCACUGAACGGAAAUUAGGCGCGCUUGGC
....((((((.<<<<<))))))...((((((..(..((((....(((....)))...))))...).))))))..(((((....))...))).>>>>>.....................

2.3.3 6xmf_C

Cryo-EM structure of Cas12g binary complex

Release date: 2021-01-13
Method: electron microscopy
Resolution: 3.10 Å
Chain length: 122
Extracted length: 119

Total clashes: 0
Clashes per residue: 0.000
Clashscore: 8.600

Description: RNA (116-MER)
Organism: metagenome

Li, Z., Zhang, H., Xiao, R., Han, R., Chang, L. (2021) Cryo-EM structure of the RNA-guided ribonuclease Cas12g. Nat.Chem.Biol.
DOI: 10.1038/s41589-020-00721-2

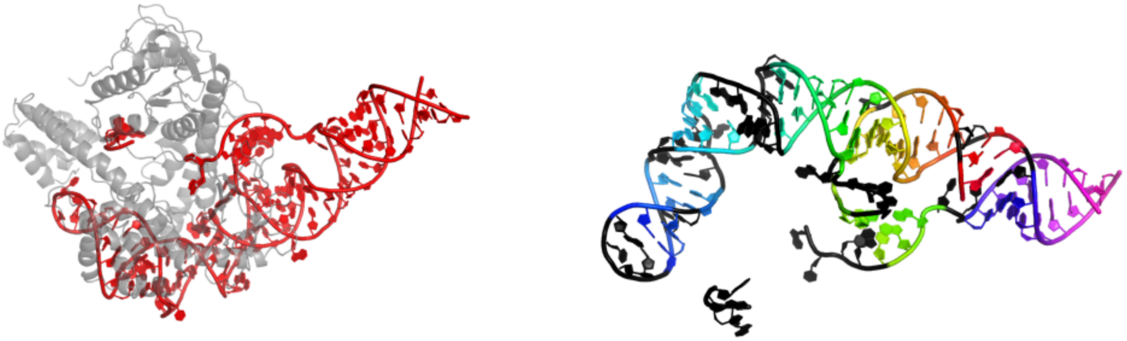

GGGAUGCUUACUUAGUCAUCUGGUUGGCAAACCUCCGCGGACCUUCGGGACCAAUGGAGAGGAACCCAGCCGAGAAGCAUCGAGCCGGUAAAUGUUUACCGGCUCUGACACCAACUGGUGAA

..((.((((.(((.<<<....((((((.....(((...((.((....)).))....)))......)))))))))))))).)).((((((((......))))))))..>>>.............

2.3.4 8dc2_B

Cryo-EM structure of CasLambda (Cas12l) bound to crRNA and DNA

Release date: 2022-12-14
Method: electron microscopy
Resolution: 2.99 Å
Chain length: 52
Extracted length: 36

Total clashes: 51
Clashes per residue: 0.981
Clashscore: 11.650

Description: RNA (51-MER)
Organism: uncultured virus

Al-Shayeb, B., Skopintsev, P., Soczek, K.M., Stahl, E.C., Li, Z., Groover, E., Smock, D., Eggers, A.R., Pausch, P., Cress, B.F., Huang, C.J., Staskawicz, B., Savage, D.F., Jacobsen, S.E., Banfield, J.F., Doudna, J.A. (2022) Diverse virus-encoded CRISPR-Cas systems include streamlined genome editors. Cell.
DOI: 10.1016/j.cell.2022.10.020

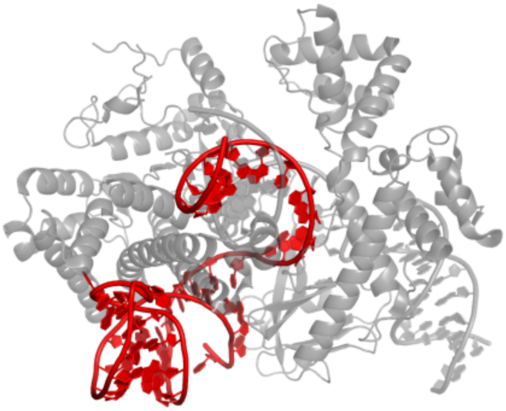

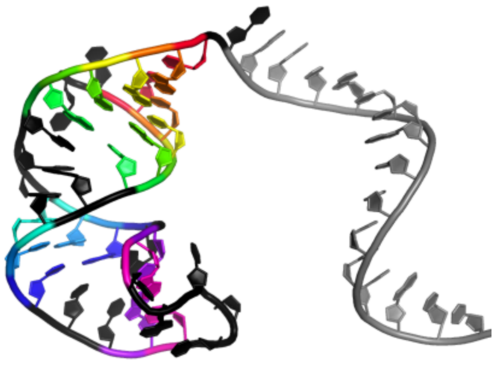

AUUGUUGUAACUCUUAUUUUGUAUGGAGUAAACAACUAGCAUCACCUUCACC
...(((((..(((..((.....)).)))...)))))................

2.4 Cas13 guide

2.4.1 6dtd_C

High-resolution crystal structure of Cas13b from Prevotella buccae

Release date: 2019-02-20
Method: x-ray diffraction
Resolution: 1.65 Å
Chain length: 37
Extracted length: 37

Total clashes: 0
Clashes per residue: 0.000
Clashscore: 4.840

Description: RNA (37-MER)
Organism: Segatella buccae

Slaymaker, I.M., Mesa, P., Kellner, M.J., Kannan, S., Brignole, E., Koob, J., Feliciano, P.R., Stella, S., Abudayyeh, O.O., Gootenberg, J.S., Strecker, J., Montoya, G., Zhang, F. (2019) High-Resolution Structure of Cas13b and Biochemical Characterization of RNA Targeting and Cleavage. Cell Rep.
DOI: 10.1016/j.celrep.2019.02.094

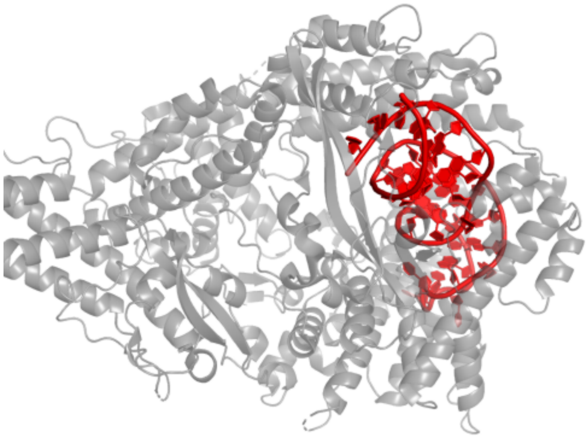

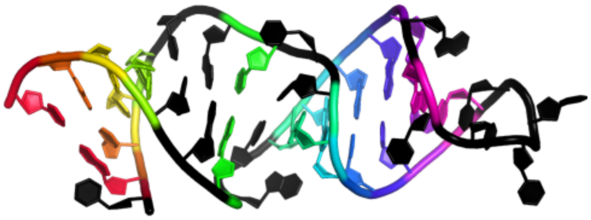

UGUUGCAUCUGCCUUCUUUUUGAAAGGUAAAAACAAC
.((((..(..((((((.....).)))))..)..))))

2.4.2 8wcs_G

Cryo-EM structure of Cas13h1-crRNA binary complex

Release date: 2024-05-22
Method: electron microscopy
Resolution: 3.10 Å
Chain length: 66
Extracted length: 66

Total clashes: 0
Clashes per residue: 0.000
Clashscore: 4.750

Description: 66-nt crRNA
Organism: synthetic construct

Chen, F., Zhang, C., Xue, J., Wang, F., Li, Z. (2024) Molecular mechanism for target RNA recognition and cleavage of Cas13h. Nucleic Acids Res.
DOI: 10.1093/nar/gkae324

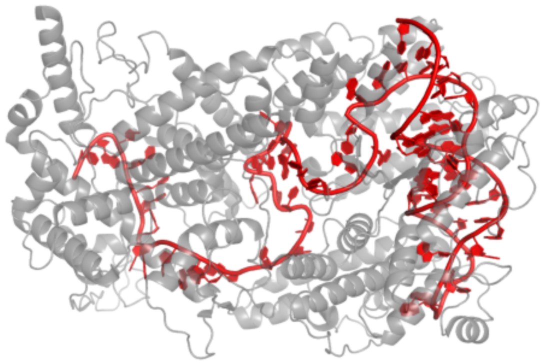

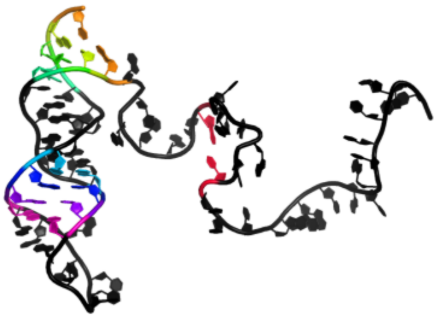

```
UGCUUCACGUAGGCCUUGGAGCCGUACAUGGUUGUAACAAGCCUAAGUUUGAAAGGUAAAAACAAC
................(......)......((((......((((.........)))).....))))
```

2.4.3 6aay_B

the Cas13b binary complex

Release date: 2019-03-13
Method: x-ray diffraction
Resolution: 2.79 Å
Chain length: 59
Extracted length: 59

Total clashes: 49
Clashes per residue: 0.831
Clashscore: 26.170

Description: RNA (52-MER)
Organism: Bergeyella zoohelcum

Zhang, B., Ye, W.W., Ye, Y.M., Zhou, H., Saeed, A.F.U.H., Chen, J., Lin, J.Y., Perculija, V., Chen, Q., Chen, C.J., Chang, M.X., Choudhary, M.I., Ouyang, S.Y. (2018) Structural insights into Cas13b-guided CRISPR RNA maturation and recognition. Cell Res.
DOI: 10.1038/s41422-018-0109-4

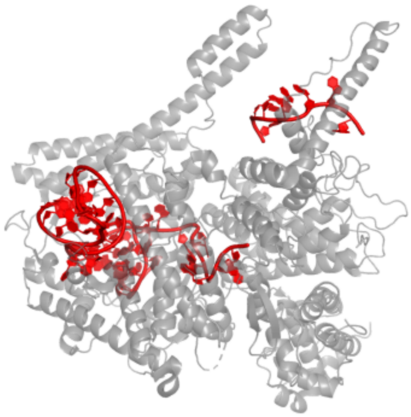

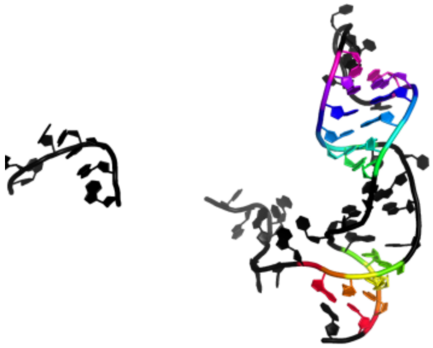

```
AAAAAGGGUUUAAAAAAUGAAAGUUGGAACUGCUCUCAUUUUGGAGGGUAAUCACAACA
......................((((.....((((((......)))))).....)))).
```

2.4.4 8ewg_B

Cryo-EM structure of a riboendonclease

Release date: 2023-08-30
Method: electron microscopy
Resolution: 2.90 Å
Chain length: 60
Extracted length: 57

Total clashes: 27
Clashes per residue: 0.450
Clashscore: 5.360

Description: RNA (56-MER)
Organism: Thermoclostridium caenicola

Wang, F., Zhang, C., Xu, H., Zeng, W., Ma, L., Li, Z. (2023) Structural Basis for the Ribonuclease Activity of a Thermostable CRISPR-Cas13a from Thermoclostridium caenicola. J.Mol.Biol.
DOI: 10.1016/j.jmb.2023.168197

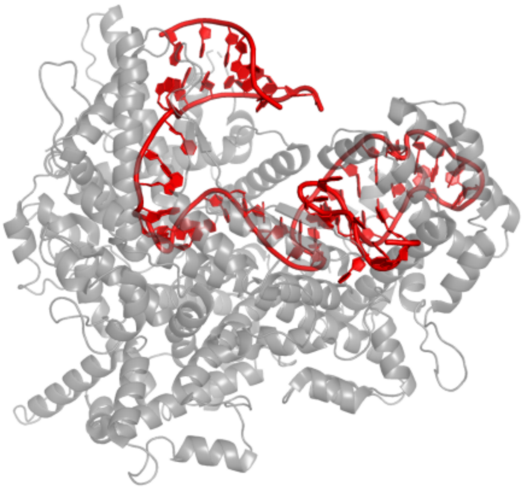

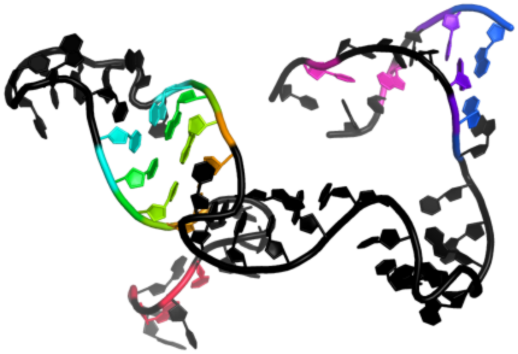

UAGGGUCACAACUCCCAUGUAGGCGGAGACUGCAACCCGAAGGUGUGACUUCCAUGCCAA
....(.)....((((.........)))).................((...(....).)).

2.5 OMEGA effector guide

2.5.1 8gkh_W

Structure of the Spizellomyces punctatus Fanzor (SpuFz) in complex with omega RNA and target DNA

Release date: 2023-07-05
Method: electron microscopy
Resolution: 2.70 Å
Chain length: 78
Extracted length: 63

Total clashes: 0
Clashes per residue: 0.000
Clashscore: 5.490

Description: RNA (78-MER)
Organism: Spizellomyces punctatus

Saito, M., Xu, P., Faure, G., Maguire, S., Kannan, S., Altae-Tran, H., Vo, S., Desimone, A., Macrae, R.K., Zhang, F. (2023) Fanzor is a eukaryotic programmable RNA-guided endonuclease. Nature.
DOI: 10.1038/s41586-023-06356-2

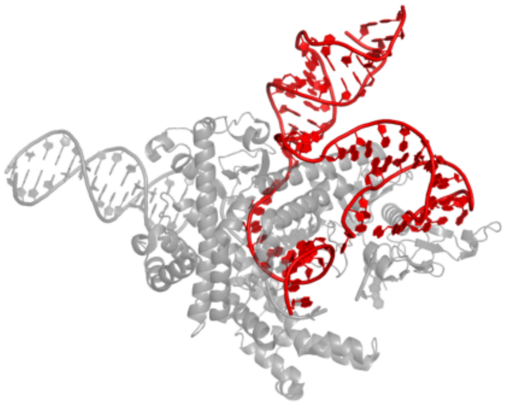

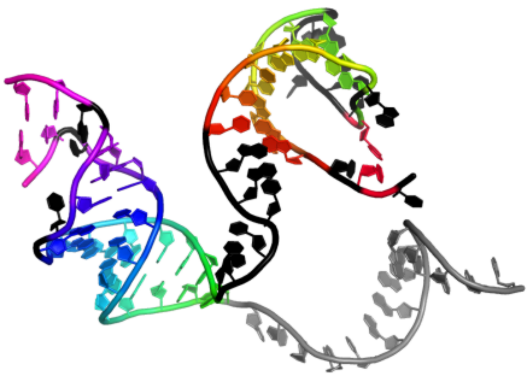

```
UCCGAGCCGGUAGUCGCGCGGUUCAAUCCCUGGUGCGGGUGCUAGUGCACCGGCUCCGCACUAUCUAUAGGUUAUGAA
.<.(((((((.....>).))))))......(((((((((.(((.((..)).))))))))))))...............
```

2.6 Fanzor guide

2.6.1 9cf2_W

Parasitella parasitica Fanzor (PpFz) State 3

Release date: 2024-09-11
Method: electron microscopy
Resolution: 3.15 Å
Chain length: 61
Extracted length: 42

Total clashes: 0
Clashes per residue: 0.000
Clashscore: 3.490

Description: Parasitella parasitica Fanzor 1 omegaRNA
Organism: Parasitella parasitica

Xu, P., Saito, M., Faure, G., Maguire, S., Chau-Duy-Tam Vo, S., Wilkinson, M.E., Kuang, H., Wang, B., Rice, W.J., Macrae, R.K., Zhang, F. (2024) Structural insights into the diversity and DNA cleavage mechanism of Fanzor. Cell.
DOI: 10.1016/j.cell.2024.07.050

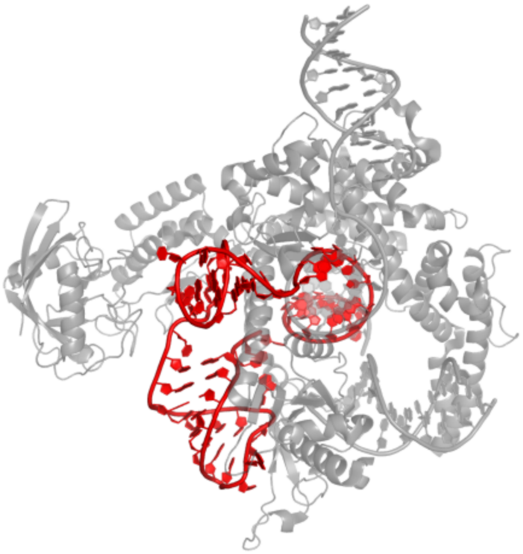

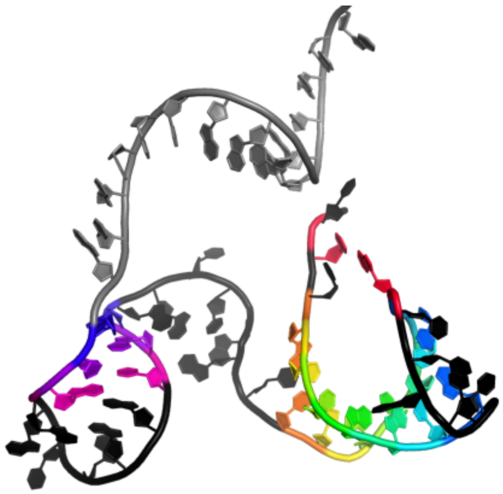

UUAUCCACCAAAGUUAUCGCUUUGGUCAAUUAAUGCAGGUAAGCAACAUCCAGCAAACAGA
....<.(((((((.....>))))))).......(((......)))................

3 IRES

3.1 IAPV IRES

3.1.1 6p5i_1

Structure of a mammalian 80S ribosome in complex with the Israeli Acute Paralysis Virus IRES (Class 1)

Release date: 2019-09-18
Method: electron microscopy
Resolution: 3.10 Å
Chain length: 253
Extracted length: 205

Total clashes: 0
Clashes per residue: 0.000
Clashscore: 2.800

Description: IAPV-IRES
Organism: Israeli acute paralysis virus

Acosta-Reyes, F., Neupane, R., Frank, J., Fernandez, I.S. (2019) The Israeli acute paralysis virus IRES captures host ribosomes by mimicking a ribosomal state with hybrid tRNAs. Embo J.
DOI: 10.15252/embj.2019102226

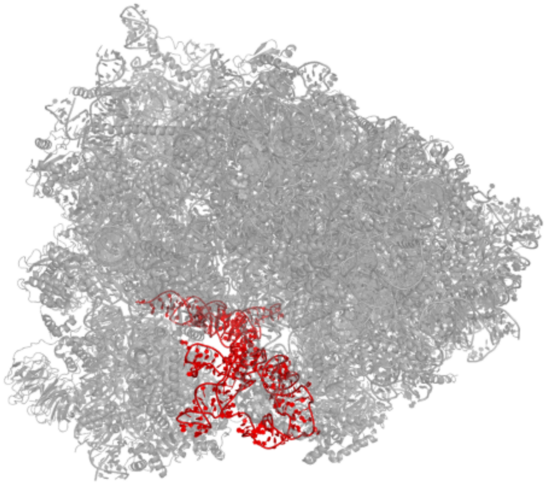

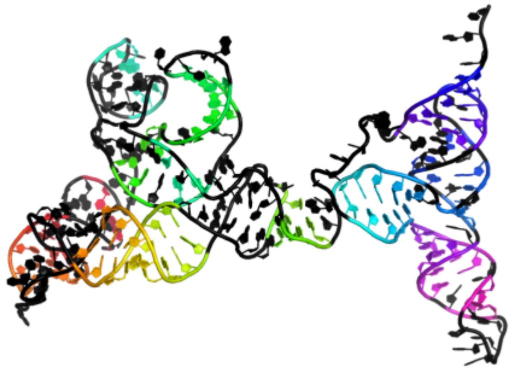

GAGCGGUUUCUGGAAUACUAUAUGUAAGUAUAGUGUUCUGGAGGCAUCAUUCUAUGGUUACCCAUCAUUAGAGGAAAUUUCCAAUAAACUCUGGUGUAAGGCUUAGAGUGAUGGUCGAGGUGCCCUAUUUAGGGUGAGGAGCCUCGGUGGCAGCCCCACCAAAUCCUCUAUUGGAUAGGAACAGCUGUACUGGGCAGUUACAGCAGUCGUAUGGUAACACAUGCGGCGUUCCGAAAUACCAUGCCUGGCGAUG

....(...(((((...........((((((((......<<<......))))))))........)))))...).......((.((((....))))...<<.))......((.....)).......>.>.....>>>..(((((.(.((.(..<<<<<..).).)).(((((.((......)).))))))))))).........>>>>>.......

3.1.2 6p5n_1

Structure of a mammalian 80S ribosome in complex with a single translocated Israeli Acute Paralysis Virus IRES and eRF1

Release date: 2019-09-25
Method: electron microscopy
Resolution: 3.20 Å
Chain length: 251
Extracted length: 207

Total clashes: 0
Clashes per residue: 0.000
Clashscore: 2.150

Description: IAPV-IRES
Organism: Israeli acute paralysis virus

Acosta-Reyes, F., Neupane, R., Frank, J., Fernandez, I.S. (2019) The Israeli acute paralysis virus IRES captures host ribosomes by mimicking a ribosomal state with hybrid tRNAs. Embo J.
DOI: 10.15252/embj.2019102226

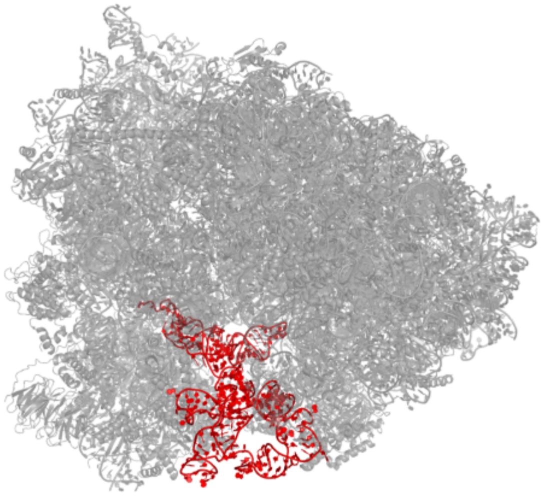

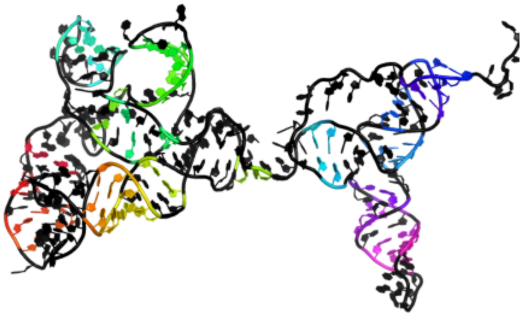

GAGCGGUUUCUGGAAUACUAUAUGUAAGUAUAGUGUUCUGGAGGCAUCAUUCUAUGGUUACCCAUCAUUAGAGGAAAUUUCCAAUAAACUCUGGUGUAAGGCUUAGAGUGAUGGUCGAGGUGCCCUAUUUAGGGUGAGGAGCCUCGGUGGCAGCCCCACCAAAUCCUCUAUUGGAUAGGAACAGCUGUACUGGGCAGUUACAGCAGUCGUAUGGUAACACAUGCGGCGUUCCGAAAUACCAUGCCUUGAUA

....(..(.((...........(((((..(........<......)..)))))........).).).)........<..((((....))))...(.(.>.....(((.....))).....)..)......>....((....((..(..<<<<..)...))..(.((.(((......))).)).)..))...........>>>>.....

3.2 CrPV 5'UTR IRES

3.2.1 6w2t_A

Structure of the Cricket Paralysis Virus 5-UTR IRES (CrPV 5-UTR-IRES) bound to the small ribosomal subunit in the closed state (Class 2)

Release date: 2020-04-22
Method: electron microscopy
Resolution: 3.36 Å
Chain length: 377
Extracted length: 339

Total clashes: 0
Clashes per residue: 0.000
Clashscore: 5.380

Description: CrPV 5'-UTR IRES
Organism: Cricket paralysis virus

Neupane, R., Pisareva, V.P., Rodriguez, C.F., Pisarev, A.V., Fernandez, I.S. (2020) A complex IRES at the 5'-UTR of a viral mRNA assembles a functional 48S complex via an uAUG intermediate. Elife.
DOI: 10.7554/eLife.54575

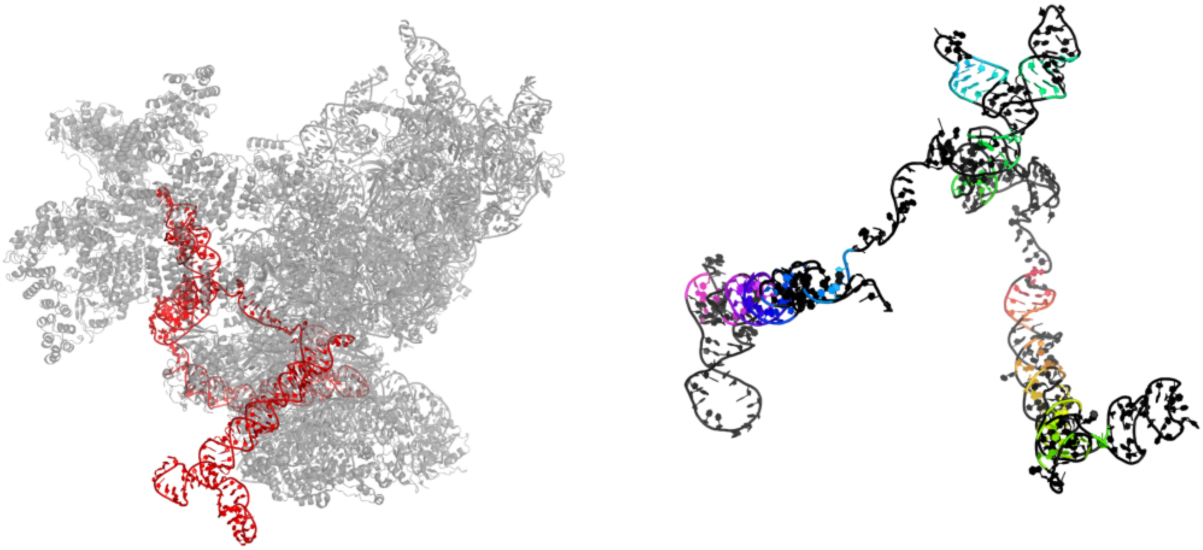

UUCGAUACCAAGAGCUGGUGUCUGAGGGAUUACGACUCGCGUGGUCGGACAGCACAUGUUAGUCAUGUAUUAUGGUGUUUCCCAACACGUAUCUUUGCUCUAGUAUUGAACUUUAGUGAUGAGUGUACUUGGCAUGUACACCUGAUUAGUUAGCCCUUGUGGAUACGCCACUGCCGCUCAGGUACAGUAGGACAUACCCCAAAUAUUGGGUUUUUUUUUUAGAGAAGAAAAUUAGUGGCGAAACGUACGGUUUAUGUCAAAGGAGAAGGUGAUUUUAAUUGCACCUGAAGCAACCCUAGGUCUUCCCUUUGAUGUGAGCUAAUAAGGAGUAACCGAUUCUUACAAUGUGAUCAUGUCUUUUCAACAAACAAAGGAUC

(.((((.......((.((...(...(((.....(..(.....).)......(.....................)..)))......)...))..).).....)))).)..............(((.........)))........................(.(......((..(........)..))......((((.....))))...).)..................((.....(....((((((((((...(.........................((........)...)...)..)))))))))).....)......))......................................

3.3 TSV IRES

3.3.1 8evp_EC

Hypopseudouridylated yeast 80S bound with Taura syndrome virus (TSV) internal ribosome entry site (IRES), Structure I

Release date: 2023-09-06
Method: electron microscopy
Resolution: 2.38 Å
Chain length: 202
Extracted length: 196

Total clashes: 0
Clashes per residue: 0.000
Clashscore: 5.910

Description: Internal ribosome entry site
Organism: Taura syndrome virus

Zhao, Y., Rai, J., Li, H. (2023) Regulation of translation by ribosomal RNA pseudouridylation. Sci Adv.
DOI: 10.1126/sciadv.adg8190

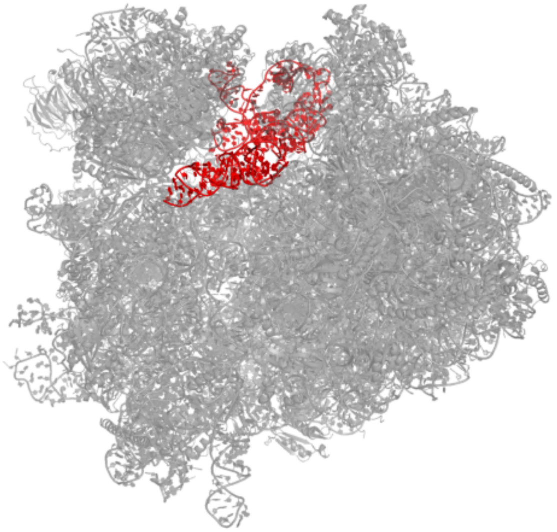

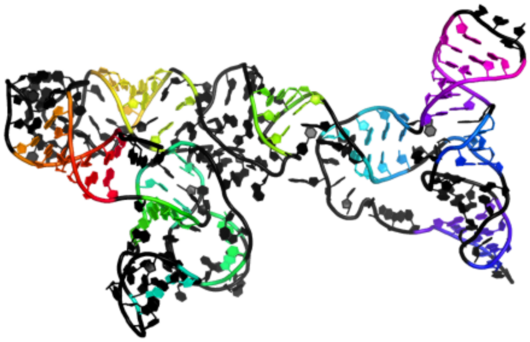

AAACUCCAUGUAUUGGUUACCCAUCUGCAUCGAAAACUCUCCGAACACUAGGUGCAGUAAGGCUUUCAUGGAGUGGUUUGCUAUUUAGCGUACGUGUACCAUAGGCAGCCCCAAAAACACGUGUGAGGAGAAAGUCCCAGUCACUUUGGGCAAAGUAGACAGCCGCGCUUGCGUGGUGGGACUUAAUUAAUGCCUGCUAACC

...((((((...............((((.(........<<<..........).))))..........)))))).(((..((......)).....<.<)))...(.......)....>.>........>>>...((((..(((.....<<<<......))).((((((....)))))).))).)........>>>>......

3.4 PSIV IGR IRES

3.4.1 4v83_CV

Crystal structure of a complex containing domain 3 from the PSIV IGR IRES RNA bound to the 70S ribosome.

Release date: 2014-07-09
Method: x-ray diffraction
Resolution: 3.50 Å
Chain length: 35
Extracted length: 35

Total clashes: 0
Clashes per residue: 0.000
Clashscore: 20.140

Description: domain 3 of PSIC IGR IRES RNA
Organism: Thermus thermophilus HB27

Zhu, J., Korostelev, A., Costantino, D.A., Donohue, J.P., Noller, H.F., Kieft, J.S. (2011) Crystal structures of complexes containing domains from two viral internal ribosome entry site (IRES) RNAs bound to the 70S ribosome. Proc.Natl.Acad.Sci.USA.
DOI: 10.1073/pnas.1018582108

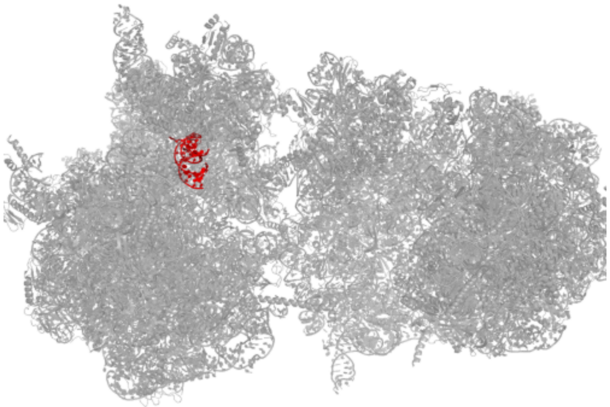

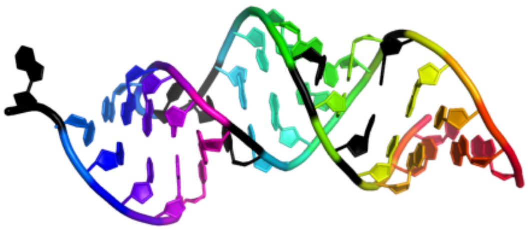

```
UCGCUCAAACAUUAAGUGGUGUUGUGCGACACUUA
((((.(.((((..<<<<<.))))).))))>>>>>.
```

4 Ribozymes

4.1 Synthetic ligase ribozyme

4.1.1 3hhn_E

Crystal structure of class I ligase ribozyme self-ligation product, in complex with U1A RBD

Release date: 2009-11-24
Method: x-ray diffraction
Resolution: 2.99 Å
Chain length: 137
Extracted length: 137

Total clashes: 58
Clashes per residue: 0.423
Clashscore: 12.640

Description: Class I ligase ribozyme, self-ligation product
Organism: nan

Shechner, D.M., Grant, R.A., Bagby, S.C., Koldobskaya, Y., Piccirilli, J.A., Bartel, D.P. (2009) Crystal structure of the catalytic core of an RNA-polymerase ribozyme. Science.
DOI: 10.1126/science.1174676

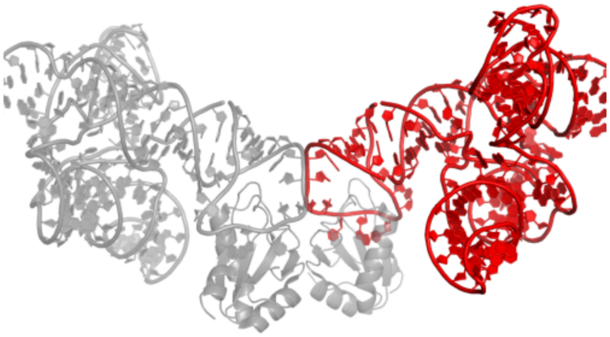

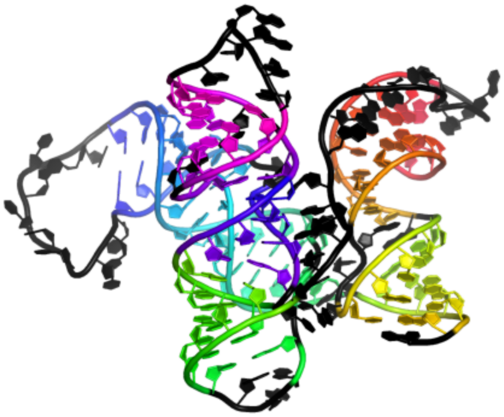

UCCAGUAGGAACACUAUACUACUGGAUAAUCAAAGACAAAUCUGCCCGAAGGGCUUGAGAACAUACCCAUUGCACUCCGGGUAUGCAGAGGUGGCAGCCUCCGGUGGGUUAAAACCCAACGUUCUCAACAAUAGUGA

((((((((...<<<<<<.))))))))...............<<<<<(...).(.((((((((((((((..........)))))))..((((.>>>>>))))((.((((......)))).)))))))))).>>>>>>.

4.1.2 3ivk_C

Crystal Structure of the Catalytic Core of an RNA Polymerase Ribozyme Complexed with an Antigen Binding Antibody Fragment

Release date: 2010-03-02
Method: x-ray diffraction
Resolution: 3.10 Å
Chain length: 128
Extracted length: 128

Total clashes: 0
Clashes per residue: 0.000
Clashscore: 31.380

Description: class I ligase product
Organism: nan

Shechner, D.M., Grant, R.A., Bagby, S.C., Koldobskaya, Y., Piccirilli, J.A., Bartel, D.P. (2009) Crystal structure of the catalytic core of an RNA-polymerase ribozyme. Science.
DOI: 10.1126/science.1174676

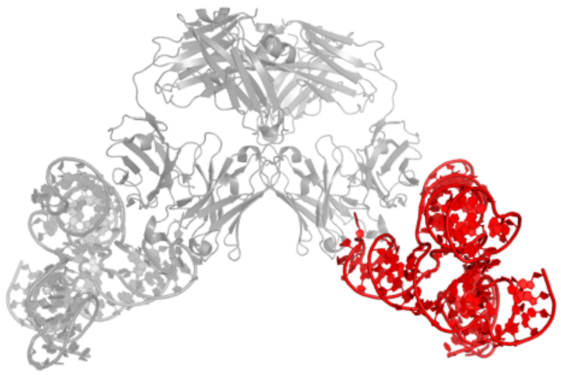

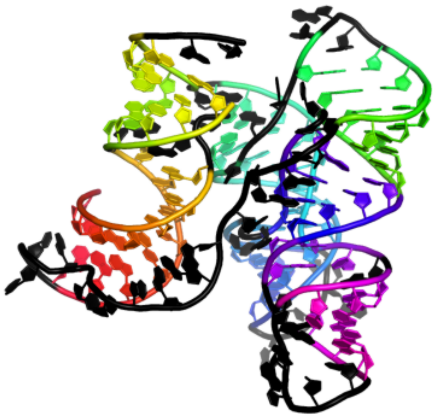

UCCAGUAGGAACACUAUACUACUGGAUAAUCAAAGACAAAUCUGCCCGAAGGGCUUGAGAACAUCGAAACACGAUGCAGAGGUGGCAGCCUCCGGUGGGUUAAAACCCAACGUUCUCAACAAUAGUGA

((((((((....<<<<<.))))))))...............<<<<<(...)...((((((((((((.....)))))..((((.>>>>>))))((.((((......)))).)))))))))..>>>>>..

4.1.3 8t2p_B

5TU-t1 - heterodimeric triplet polymerase ribozyme

Release date: 2024-01-24
Method: electron microscopy
Resolution: 5.00 Å
Chain length: 152
Extracted length: 152

Total clashes: 0
Clashes per residue: 0.000
Clashscore: 0.000

Description: RNA (152-MER)
Organism: synthetic construct

McRae, E.K.S., Wan, C.J.K., Kristoffersen, E.L., Hansen, K., Gianni, E., Gallego, I., Curran, J.F., Attwater, J., Holliger, P., Andersen, E.S. (2024) Cryo-EM structure and functional landscape of an RNA polymerase ribozyme. Proc.Natl.Acad.Sci.USA.
DOI: 10.1073/pnas.2313332121

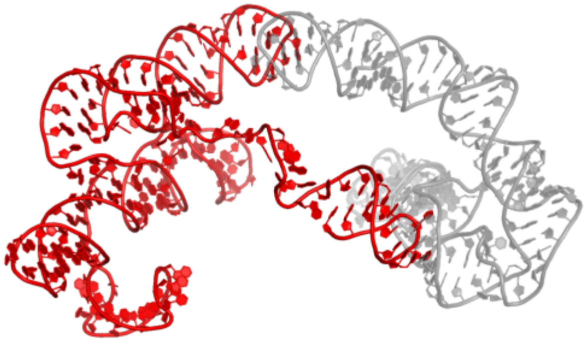

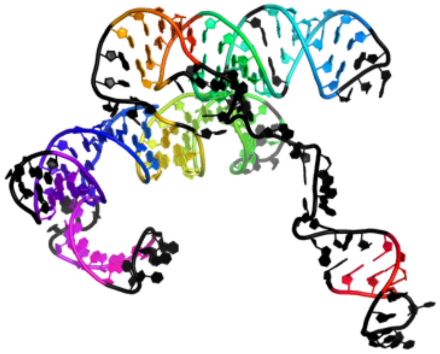

GGAUCUUCUCGAUCUAACAAAAAAGACAAAUCUGCCACAAAGCUUGAGAGCAUCUUCGGAUGCAGAGGCGGCAGCCUUCGGUGGCGCGAUAGCGCCAACGUUCUCAACUAUGACACGCAAAACGCGUGCUCCGUUGAAUGGAGUUUAUCAUG

.(((.......))).................<<<<<.....(.((((((((((.(...))))).((((.>>>>>))))((.((((((....)))))).)).)))))))((((((((((.....)))))(((((.....)))))....)))))

4.2 VS ribozyme

4.2.1 4r4v_A

Crystal structure of the VS ribozyme - G638A mutant

Release date: 2015-09-30
Method: x-ray diffraction
Resolution: 3.07 Å
Chain length: 186
Extracted length: 186

Total clashes: 38
Clashes per residue: 0.204
Clashscore: 3.260

Description: VS ribozyme RNA
Organism: Neurospora

Suslov, N.B., DasGupta, S., Huang, H., Fuller, J.R., Lilley, D.M., Rice, P.A., Piccirilli, J.A. (2015) Crystal structure of the Varkud satellite ribozyme. Nat.Chem.Biol.
DOI: 10.1038/nchembio.1929

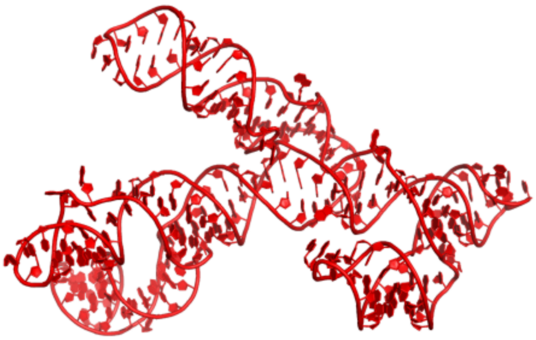

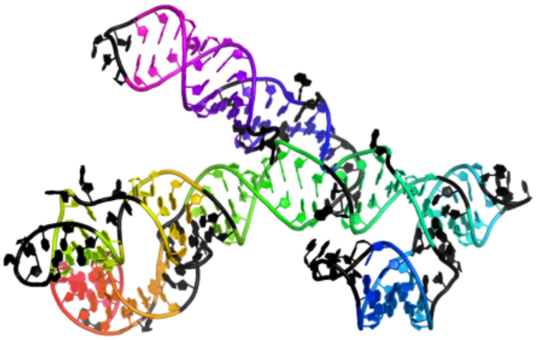

GGCGCUGUGUCGCAAUCUGCGAAGGGCGUCGUCGGCCCAAGCGGUAGUAAGCAGGGAACUCACCUCCAAUGAAACACAUUGUCGUAGCAGUUGACUACUGUUAUGUGAUUGGUAGAGGCUAAGUGACGGUAUUGGCGUAAGCCAAUACCGCAGCACAGCACAAGCCCGCUUGCGAGAUUACAGCGC

.((((((((((.....((((...((((.......))))..))))..((((((.(((..((((((..(((((.....)))))..(((((((.......)))))))......))).)))(((..(((.((((((((((....))))))))))...))))))......)))))))))..))).)))))))

4.3 Self-alkylating ribozyme

4.3.1 6xjq_A

Crystal structure of a self-alkylating ribozyme - alkylated form with biotinylated epoxide substrate

Release date: 2022-01-19
Method: x-ray diffraction
Resolution: 1.71 Å
Chain length: 58
Extracted length: 58

Total clashes: 10
Clashes per residue: 0.172
Clashscore: 2.170

Description: Self-alkylating ribozyme (58-MER)
Organism: Aeropyrum pernix

Krochmal, D., Shao, Y., Li, N.S., DasGupta, S., Shelke, S.A., Koirala, D., Piccirilli, J.A. (2022) Structural basis for substrate binding and catalysis by a self-alkylating ribozyme. Nat.Chem.Biol.
DOI: 10.1038/s41589-021-00950-z

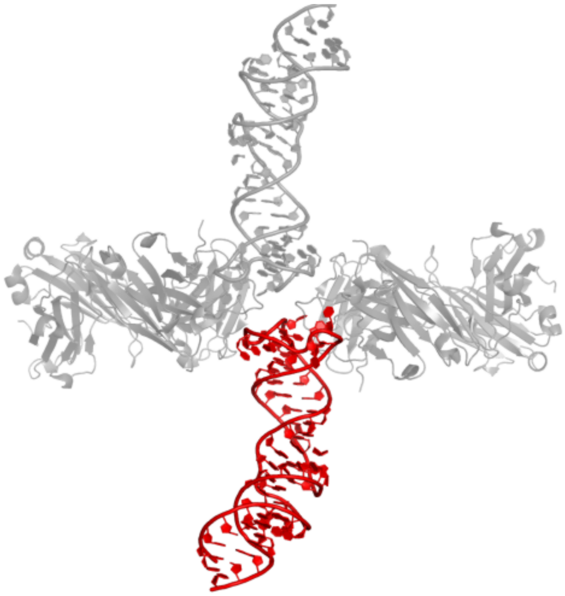

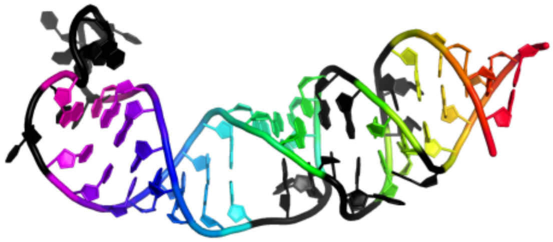

```
GGCCGCUCCAGAAGAGGGCCCCCUUGCCCGUUAUCGGGGGCUAGGCUCGAUGUCGGCC
(((((...((...((((((((((..(........))))))))...)))..)).)))))
```

4.4 Diels-Alder ribozyme

4.4.1 1ykv_D

Crystal structure of the Diels-Alder ribozyme complexed with the product of the reaction between N-pentylmaleimide and covalently attached 9-hydroxymethylanthracene

Release date: 2005-02-22
Method: x-ray diffraction
Resolution: 3.30 Å
Chain length: 38
Extracted length: 31

Total clashes: 26
Clashes per residue: 0.684
Clashscore: 12.520

Description: Diels-Alder ribozyme
Organism: nan

Serganov, A., Keiper, S., Malinina, L., Tereshko, V., Skripkin, E., Hobartner, C., Polonskaia, A., Phan, A.T., Wombacher, R., Micura, R., Dauter, Z., Jaschke, A., Patel, D.J. (2005) Structural basis for Diels-Alder ribozyme-catalyzed carbon-carbon bond formation. Nat.Struct.Mol.Biol.
DOI: 10.1038/nsmb906

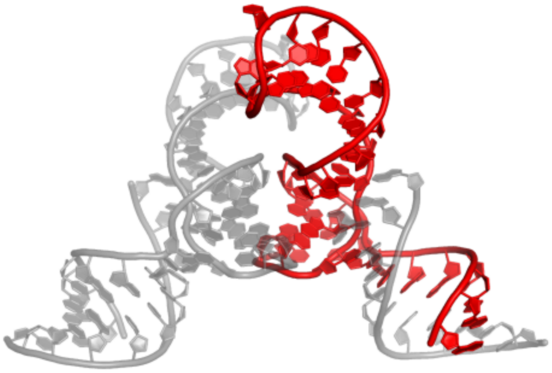

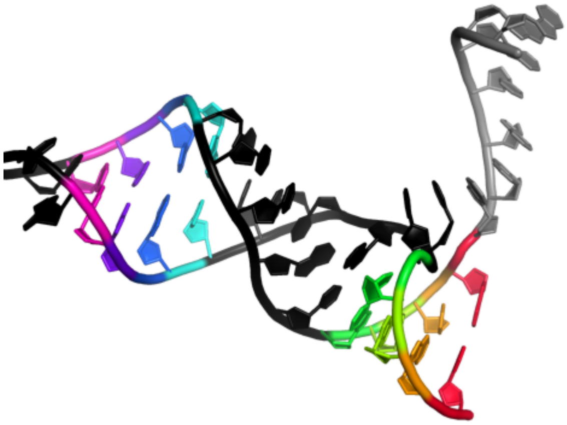

GGGCGAGGCCGUGCCAGCUCUUCGGAGCAAUACUCGGC
.......((((.....((((....))))......))))

4.4.2 1yls_D

Crystal structure of selenium-modified Diels-Alder ribozyme complexed with the product of the reaction between N-pentylmaleimide and covalently attached 9-hydroxymethylanthracene

Release date: 2005-02-22
Method: x-ray diffraction
Resolution: 3.00 Å
Chain length: 38
Extracted length: 31

Total clashes: 43
Clashes per residue: 1.132
Clashscore: 14.760

Description: RNA Diels-Alder ribozyme
Organism: nan

Serganov, A., Keiper, S., Malinina, L., Tereshko, V., Skripkin, E., Hobartner, C., Polonskaia, A., Phan, A.T., Wombacher, R., Micura, R., Dauter, Z., Jaschke, A., Patel, D.J. (2005) Structural basis for Diels-Alder ribozyme-catalyzed carbon-carbon bond formation. Nat.Struct.Mol.Biol.
DOI: 10.1038/nsmb906

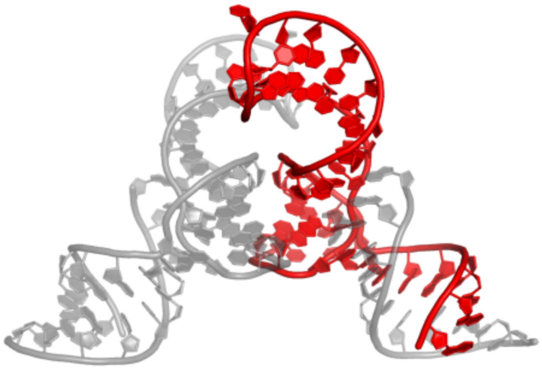

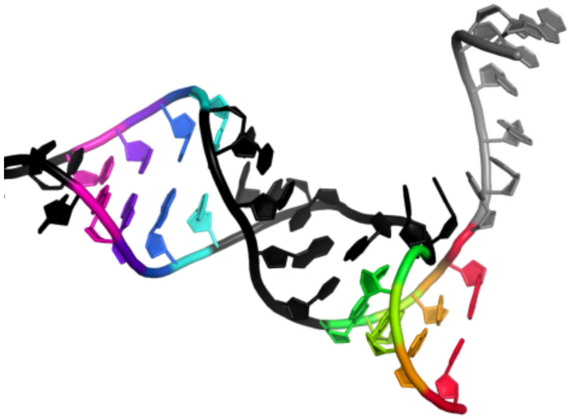

GGGCGAGGCCGUGCCGGCUCUUCGGAGCAAUACUCGGC
.......((((.....((((....))))......))))

4.5 Methyltransferase ribozyme

4.5.1 7dlz_Y

Crystal Structure of Methyltransferase Ribozyme

Release date: 2021-10-27
Method: x-ray diffraction
Resolution: 3.00 Å
Chain length: 45
Extracted length: 45

Total clashes: 0
Clashes per residue: 0.000
Clashscore: 8.240

Description: RNA (45-MER)
Organism: synthetic construct

Jiang, H.Y., Gao, Y.Q., Zhang, L., Chen, D.R., Gan, J.H., Murchie, A.I.H. (2021) The identification and characterization of a selected SAM-dependent methyltransferase ribozyme that is present in natural sequences. Nat Catal.
DOI: 10.1038/s41929-021-00685-z

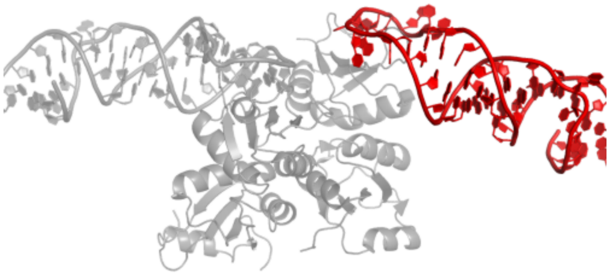

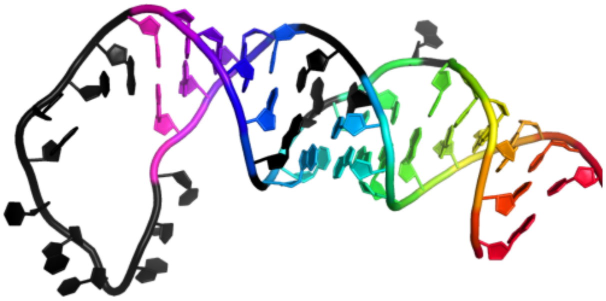

```
GGACCUACUACGAGCGCCAUUGCACUCCGGCGCCACGGGGGGUCC
((((((.((.((.(((((..........)))))..))))))))))
```

4.5.2 7v9e_A

Crystal structure of a methyl transferase ribozyme

Release date: 2022-03-23
Method: x-ray diffraction
Resolution: 2.30 Å
Chain length: 68
Extracted length: 68

Total clashes: 4
Clashes per residue: 0.059
Clashscore: 1.360

Description: RNA (68-MER)
Organism: Homo sapiens

Deng, J., Wilson, T.J., Wang, J., Peng, X., Li, M., Lin, X., Liao, W., Lilley, D.M.J., Huang, L. (2022) Structure and mechanism of a methyltransferase ribozyme. Nat.Chem.Biol.
DOI: 10.1038/s41589-022-00982-z

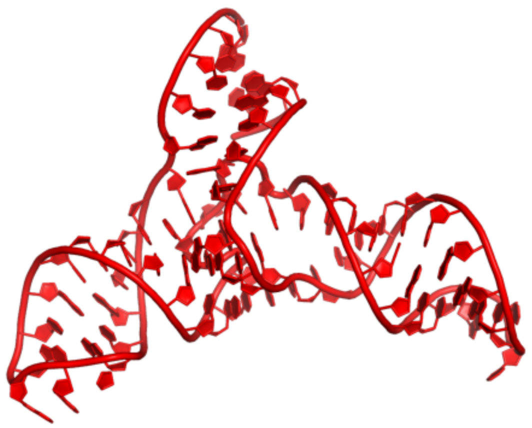

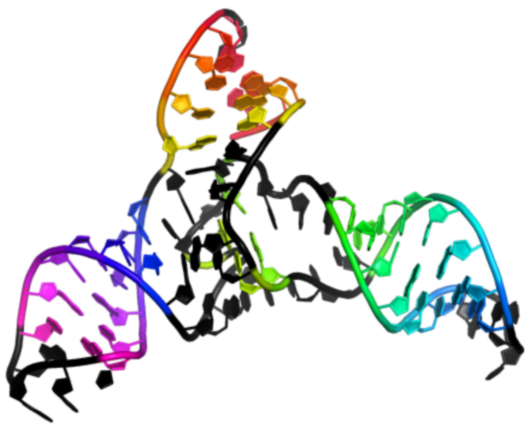

CGGGCUGACCGACCCCCCGAGUUCGCUCGGGGACAACUAGACAUACAGUAUGAAAAUACUGAGCCCGC
(((((....((...(((((((....)))))))....)..).....((((((....)))))).))))).

5 Repeats

5.1 r(CUG)

5.1.1 4pcj_A

Modifications to toxic CUG RNAs induce structural stability and rescue mis-splicing in Myotonic Dystrophy

Release date: 2014-10-29
Method: x-ray diffraction
Resolution: 1.90 Å
Chain length: 35
Extracted length: 35

Total clashes: 0
Clashes per residue: 0.000
Clashscore: 0.000

Description: trCUG-3('5)
Organism: synthetic construct

deLorimier, E., Coonrod, L.A., Copperman, J., Taber, A., Reister, E.E., Sharma, K., Todd, P.K., Guenza, M.G., Berglund, J.A. (2014) Modifications to toxic CUG RNAs induce structural stability, rescue mis-splicing in a myotonic dystrophy cell model and reduce toxicity in a myotonic dystrophy zebrafish model. Nucleic Acids Res.
DOI: 10.1093/nar/gku941

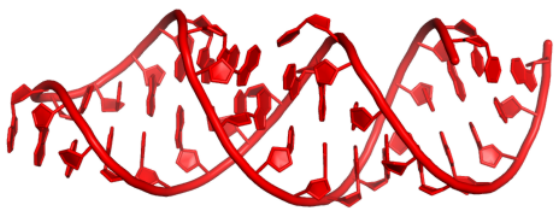

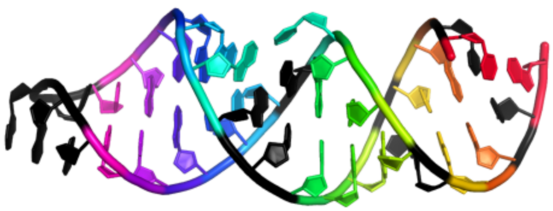

CUGCUGGCUAAGGCAUGAAAGUGCUAUGCCUGCUG
(.((.(((..((((((....))))).)))).)).)

5.2 r(CCUG)

5.2.1 4k27_U

Myotonic Dystrophy Type 2 RNA: Structural Studies and Designed Small Molecules that Modulate RNA Function

Release date: 2013-11-27
Method: x-ray diffraction
Resolution: 2.35 Å
Chain length: 55
Extracted length: 55

Total clashes: 0
Clashes per residue: 0.000
Clashscore: 1.170

Description: Myotonic Dystrophy Type 2 RNA
Organism: nan

Childs-Disney, J., Yildirim, I., Park, H., Lohman, J., Guan, L., Tran, T., Sarkar, P., Schatz, G.C., Disney, M.D. (2013) Myotonic Dystrophy Type 2 RNA: Structural Studies and Designed Small Molecules that Modulate RNA Function. ACS CHEM.BIOL.
DOI: nan

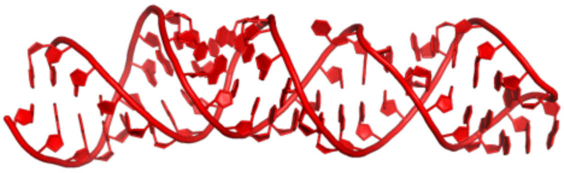

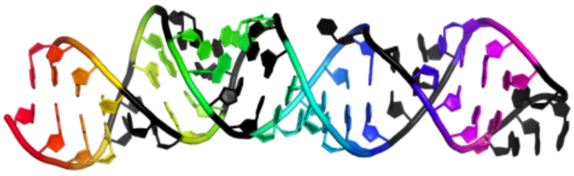

GCCCCUGCCUGCCUGCAGCUAAGGAUGAAAGUCUAUGCUGCCUGCCUGCCUGGGC
((((..((..((..(((((...((((....))))..)))))..))..))..))))

5.3 r(AUUCU)

5.3.1 5btm_A

Crystal structure of AUUCU repeating RNA that causes spinocerebellar ataxia type 10 (SCA10)

Release date: 2015-07-15
Method: x-ray diffraction
Resolution: 2.78 Å
Chain length: 55
Extracted length: 43

Total clashes: 6
Clashes per residue: 0.109
Clashscore: 2.200

Description: RNA (55-mer)
Organism: Homo sapiens

Park, H., Gonzalez, A.L., Yildirim, I., Tran, T., Lohman, J.R., Fang, P., Guo, M., Disney, M.D. (2015) Crystallographic and Computational Analyses of AUUCU Repeating RNA That Causes Spinocerebellar Ataxia Type 10 (SCA10). Biochemistry.
DOI: 10.1021/acs.biochem.5b00551

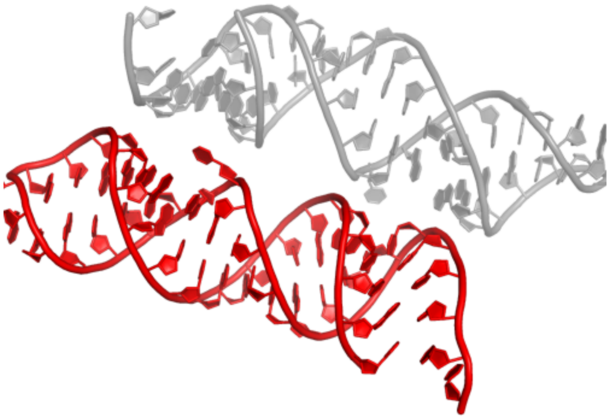

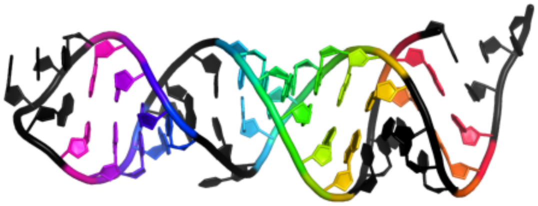

GUCAUUCUAUUCUAUCGGCUAAGGAUGAAAGUCUAUGCCGAUUCUAUUCUAUGGC
........((...((((((...((((....))))..))))))...))........

6 Miscellaneous (synthetic)

6.1 Nanoarchitecture 1

6.1.1 7jrr_A

Crystal structures of artificially designed homomeric RNA nanoarchitectures

Release date: 2021-09-08
Method: x-ray diffraction
Resolution: 2.16 Å
Chain length: 51
Extracted length: 51

Total clashes: 14
Clashes per residue: 0.275
Clashscore: 4.790

Description: RNA (50-MER)
Organism: synthetic construct

Liu, D., Shao, Y., Piccirilli, J.A., Weizmann, Y. (2021) Structures of artificially designed discrete RNA nanoarchitectures at near-atomic resolution. Sci Adv.
DOI: 10.1126/sciadv.abf4459

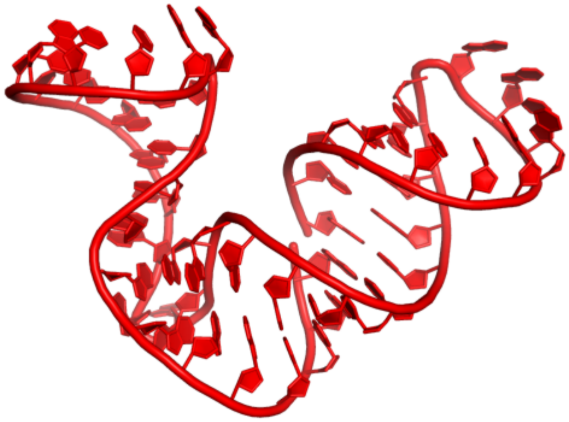

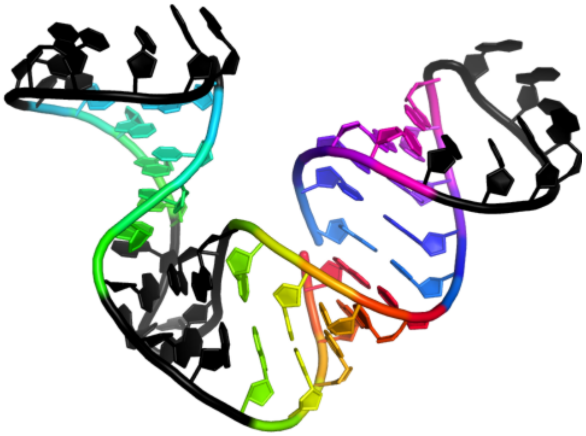

GGACGGGAGCUGAACCAUCCAGCGAAGAACGUCCCGACGGAUGGUUCGUCG
(((((...((((.......))))......)))))(((((.......)))))

6.2 Nanoarchitecture 2

6.2.1 7jrs_A

Crystal structures of artificially designed homomeric RNA nanoarchitectures

Release date: 2021-09-08
Method: x-ray diffraction
Resolution: 3.21 Å
Chain length: 129
Extracted length: 129

Total clashes: 19
Clashes per residue: 0.147
Clashscore: 3.950

Description: RNA 3D nanocage
Organism: synthetic construct

Liu, D., Shao, Y., Piccirilli, J.A., Weizmann, Y. (2021) Structures of artificially designed discrete RNA nanoarchitectures at near-atomic resolution. Sci Adv.
DOI: 10.1126/sciadv.abf4459

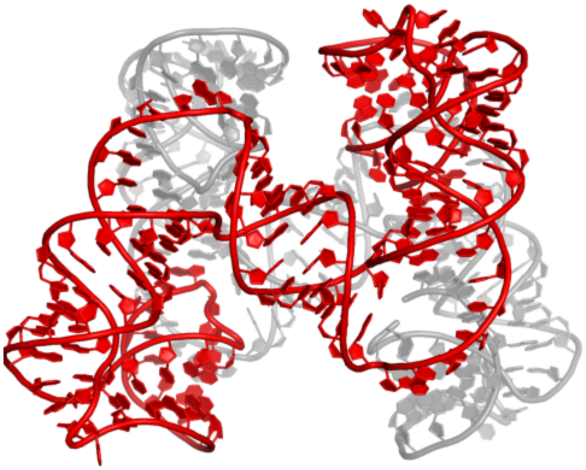

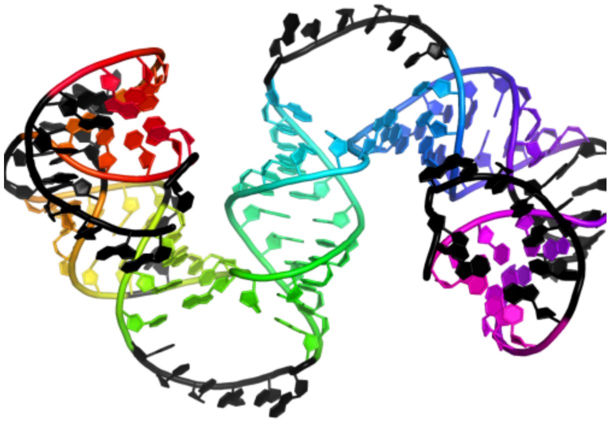

```
GGACGCGGUAGCGAAGAACCAUCGACUGCGACUUAGCGAGCCUGCCCUGGCAGUGGGAGCCUGAAGCAGGCACGGGCGAAGAACACUGUCAGGUCGCUGAGUCGGCGUCCCAGUUGAUGGGGAGCUACC
.......(((((......(((((.(((((((((((((((......((((((((((...(((((.........)))))......)))))))))))))))))))))......)))).)))))...))))).
```

6.3 Synthetic hairpin

6.3.1 6az4_A

RNA hairpin complex with guanosine dinucleotide ligand G(5')ppp(5')G

Release date: 2018-02-21
Method: x-ray diffraction
Resolution: 2.98 Å
Chain length: 32
Extracted length: 21

Total clashes: 0
Clashes per residue: 0.000
Clashscore: 0.720

Description: RNA (32-MER)
Organism: synthetic construct

Zhang, W., Tam, C.P., Zhou, L., Oh, S.S., Wang, J., Szostak, J.W. (2018) Structural Rationale for the Enhanced Catalysis of Nonenzymatic RNA Primer Extension by a Downstream Oligonucleotide. J. Am. Chem. Soc.
DOI: 10.1021/jacs.7b11750

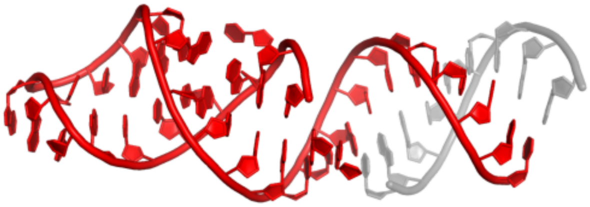

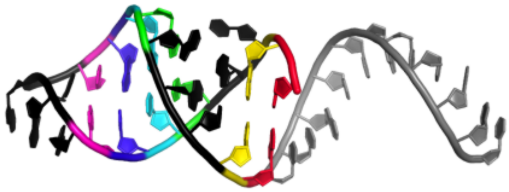

CUGCUGCUGCCGCUAAGGAUGAAAGUCUAUGC
...........((...((((....))))..))

6.4 Synthetic tetraloop-tetraloop receptor

6.4.1 6dvk_H

Computationally designed mini tetraloop-tetraloop receptor by the RNAMake program - construct 6 (miniTTR 6)

Release date: 2019-06-26
Method: x-ray diffraction
Resolution: 2.55 Å
Chain length: 95
Extracted length: 95

Total clashes: 0
Clashes per residue: 0.000
Clashscore: 1.980

Description: RNA (95-MER)
Organism: synthetic construct

Yesselman, J.D., Eiler, D., Carlson, E.D., Gotrik, M.R., d'Aquino, A.E., Ooms, A.N., Kladwang, W., Carlson, P.D., Shi, X., Costantino, D.A., Herschlag, D., Lucks, J.B., Jewett, M.C., Kieft, J.S., Das, R. (2019) Computational design of three-dimensional RNA structure and function. Nat Nanotechnol.
DOI: 10.1038/s41565-019-0517-8

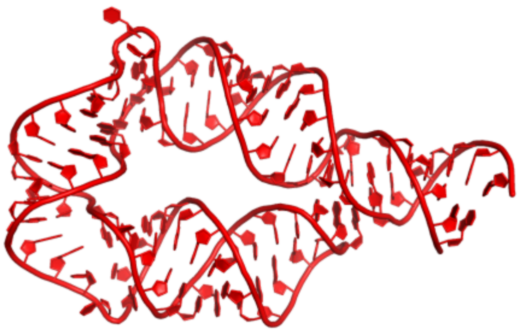

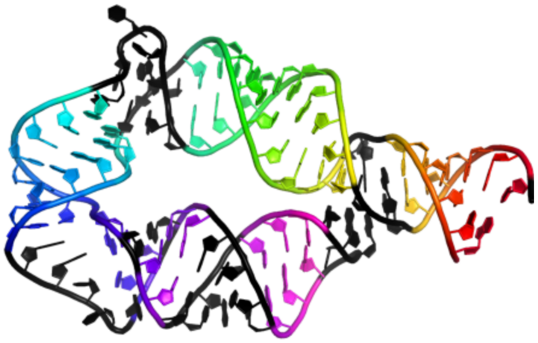

GUUGAUAUGGAUUUACUCCGAGGAGACGAACUACCACGAACAGGGGAAACUCUACCCGUGGCGUCUCCGUUUGACGAGUAAGUCCUAAGUCAACA
((((((..((((((((((...(((((((.....((.((....(((....)))....)).))))))))).......))))))))))...)))))).

6.5 Synthetic G-quadruplex

6.5.1 5dea_C

Crystal structure of the complex between human FMRP RGG motif and G-quadruplex RNA, cesium bound form.

Release date: 2015-09-23
Method: x-ray diffraction
Resolution: 2.80 Å
Chain length: 35
Extracted length: 35

Total clashes: 4
Clashes per residue: 0.114
Clashscore: 3.840

Description: sc1
Organism: synthetic construct

Vasilyev, N., Polonskaia, A., Darnell, J.C., Darnell, R.B., Patel, D.J., Serganov, A. (2015) Crystal structure reveals specific recognition of a G-quadruplex RNA by a beta-turn in the RGG motif of FMRP. Proc.Natl.Acad.Sci.USA.
DOI: 10.1073/pnas.1515737112

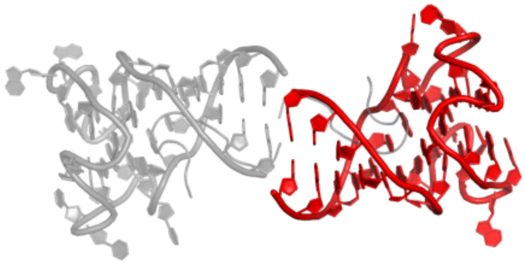

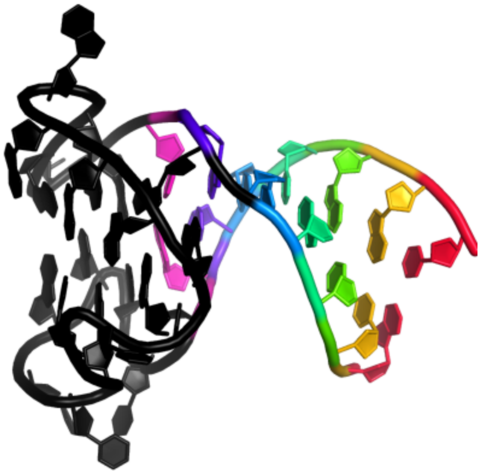

GCUGCGGUGUGGAAGGAGUGGCUGGGUUGCGCAGC
(((((.((...................)))))))

7 Miscellaneous

7.1 ToXI

7.1.1 7d8o_L

Crystal structure of E. coli ToxIN type III toxin-antitoxin complex

Release date: 2022-01-05
Method: x-ray diffraction
Resolution: 2.10 Å
Chain length: 37
Extracted length: 37

Total clashes: 4
Clashes per residue: 0.108
Clashscore: 2.640

Description: Antitoxin RNA
Organism: Escherichia coli

Manikandan, P., Sandhya, S., Nadig, K., Paul, S., Srinivasan, N., Rothweiler, U., Singh, M. (2022) Identification, functional characterization, assembly and structure of ToxIN type III toxin-antitoxin complex from E. coli. Nucleic Acids Res.
DOI: 10.1093/nar/gkab1264

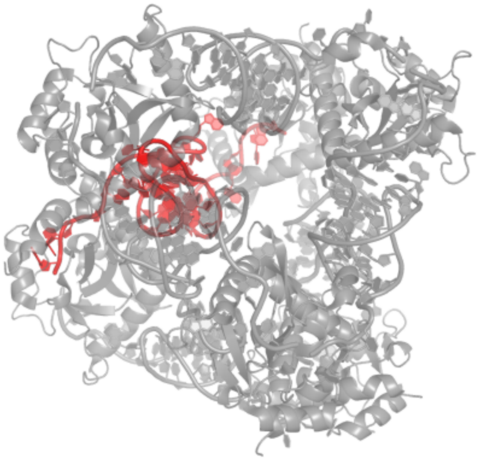

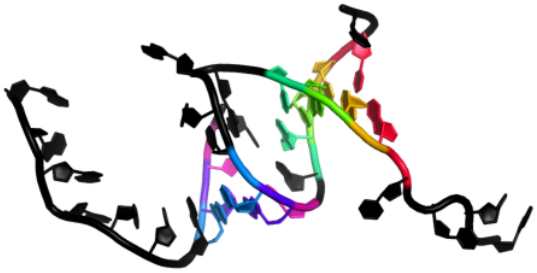

AUUUAGGUGAUUUGCUACCUUUAAGUGCAGCUAGAAA
....((((....<<<.))))......>>>........

7.1.2 2xdb_G

A processed non-coding RNA regulates a bacterial antiviral system

Release date: 2011-01-12
Method: x-ray diffraction
Resolution: 2.55 Å
Chain length: 40
Extracted length: 36

Total clashes: 0
Clashes per residue: 0.000
Clashscore: 12.800

Description: TOXI
Organism: Pectobacterium atrosepticum

Blower, T.R., Pei, X.Y., Short, F.L., Fineran, P.C., Humphreys, D.P., Luisi, B.F., Salmond, G.P.C. (2011) A Processed Noncoding RNA Regulates an Altruistic Bacterial Antiviral System. Nat.Struct.Mol.Biol.
DOI: 10.1038/NSMB.1981

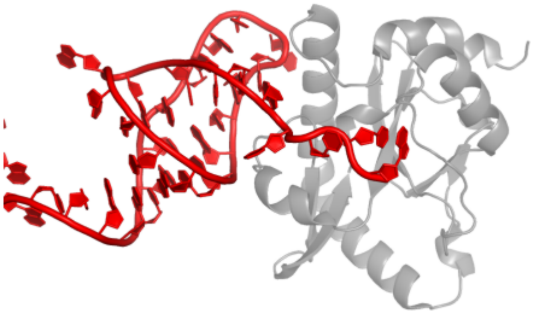

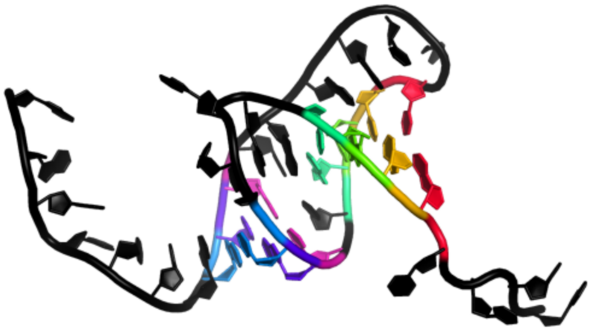

```
AUUCAGGUGAUUUGCUACCUUUAAGUGCAGCUAGAAAUUC
....((((....<<<.))))......>>>...........
```

7.1.3 4rmo_H

Crystal Structure of the CptIN Type III Toxin-Antitoxin System from Eubacterium rectale

Release date: 2015-09-30
Method: x-ray diffraction
Resolution: 2.20 Å
Chain length: 45
Extracted length: 45

Total clashes: 19
Clashes per residue: 0.422
Clashscore: 6.710

Description: RNA (45-MER)
Organism: synthetic construct

Rao, F., Short, F.L., Voss, J.E., Blower, T.R., Orme, A.L., Whittaker, T.E., Luisi, B.F., Salmond, G.P. (2015) Co-evolution of quaternary organization and novel RNA tertiary interactions revealed in the crystal structure of a bacterial protein-RNA toxin-antitoxin system. Nucleic Acids Res.
DOI: 10.1093/nar/gkv868

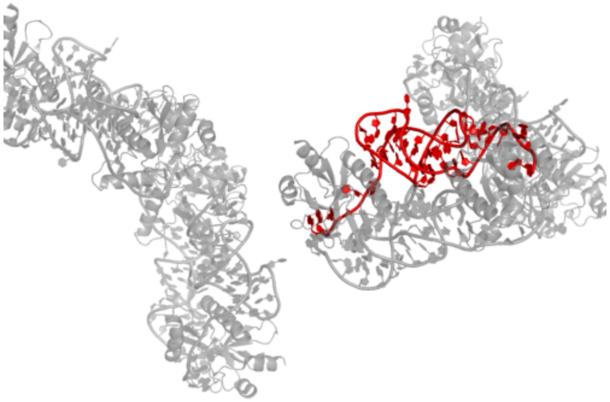

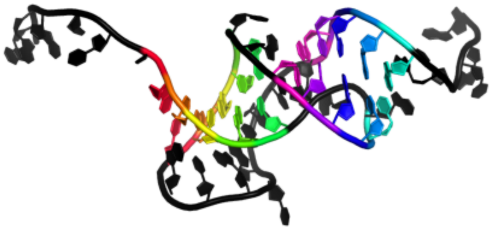

```
AAGUUUACCACUGACCGAUAUGUGGUAUAUAAAUGGUCGGGUUGA
......(((((...<<<<<..)))))).........>>>>>.....
```

7.2 Structured part of acrIF8-aca2 5' UTR

7.2.1 8w35_C

Aca2 from Pectobacterium phage ZF40 bound to RNA

Release date: 2024-07-24
Method: electron microscopy
Resolution: 2.61 Å
Chain length: 42
Extracted length: 37

Total clashes: 0
Clashes per residue: 0.000
Clashscore: 0.620

Description: IR2 and IR-RBS RNA
Organism: Pectobacterium phage ZF40

Birkholz, N., Kamata, K., Feussner, M., Wilkinson, M.E., Cuba Samaniego, C., Migur, A., Kimanius, D., Ceelen, M., Went, S.C., Usher, B., Blower, T.R., Brown, C.M., Beisel, C.L., Weinberg, Z., Fagerlund, R.D., Jackson, S.A., Fineran, P.C. (2024) Phage anti-CRISPR control by an RNA- and DNA-binding helix-turn-helix protein. Nature.
DOI: 10.1038/s41586-024-07644-1

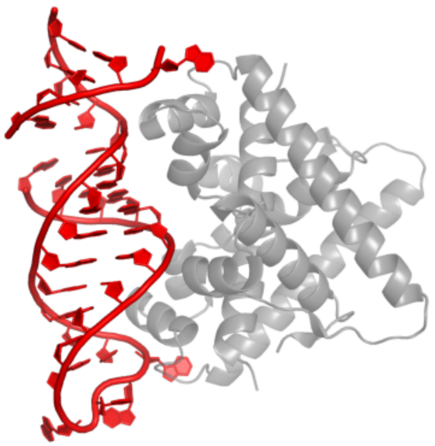

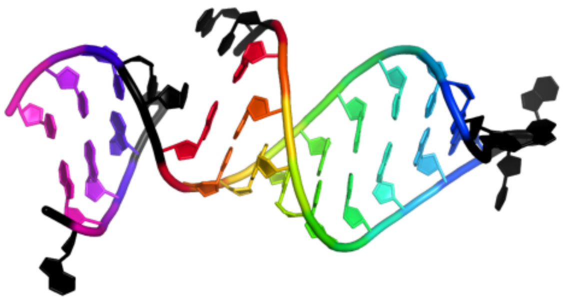

```
AUCGGUUCGAGAUGGCUCGAAUCGCUCCUAACGAGGAUUCCA
..(((((((((....)))))))))..(((....)))......
```

7.3 Saguaro cactus viral mRNA structure

7.3.1 8t2a_R

Crystal structure of SCV PTE G18A mutant RNA in complex with Fab BL3-6

Release date: 2024-01-10
Method: x-ray diffraction
Resolution: 3.17 Å
Chain length: 90
Extracted length: 90

Total clashes: 55
Clashes per residue: 0.611
Clashscore: 9.760

Description: RNA (90-MER)
Organism: Saguaro cactus virus

Ojha, M., Vogt, J., Das, N.K., Redmond, E., Singh, K., Banna, H.A., Sadat, T., Koirala, D. (2024) Structure of saguaro cactus virus 3' translational enhancer mimics 5' cap for eIF4E binding. Proc.Natl.Acad.Sci.USA.
DOI: 10.1073/pnas.2313677121

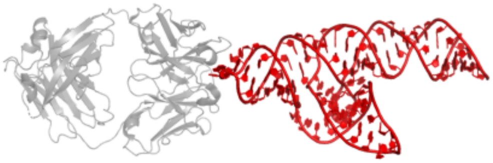

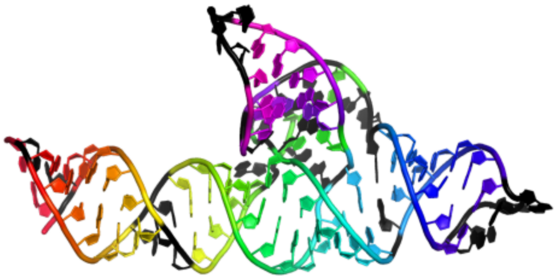

GGUUGCUCGACUGUGAGAGGACCUACCCACUGUGGAAACACCACAGGAACUUCCAACCUUCGGGUGGCGAGGUAGGGCAGAAGAGUGACC
(.((((((..((((..<...(((((((.((((((.....))))))...>..((.(((....)))))).))))))))))..)))))).)

7.3.2 8t29_R

Crystal structure of SCV PTE RNA in complex with Fab BL3-6

Release date: 2024-01-10
Method: x-ray diffraction
Resolution: 3.13 Å
Chain length: 90
Extracted length: 90

Total clashes: 61
Clashes per residue: 0.678
Clashscore: 10.320

Description: RNA (90-MER)
Organism: Saguaro cactus virus

Ojha, M., Vogt, J., Das, N.K., Redmond, E., Singh, K., Banna, H.A., Sadat, T., Koirala, D. (2024) Structure of saguaro cactus virus 3' translational enhancer mimics 5' cap for eIF4E binding. Proc.Natl.Acad.Sci.USA.
DOI: 10.1073/pnas.2313677121

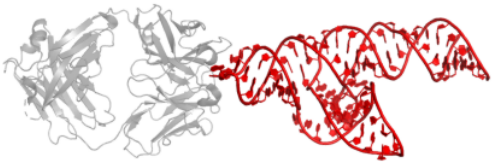

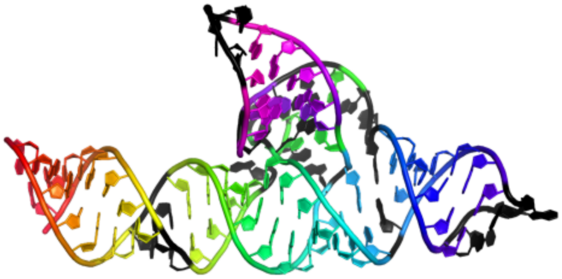

GGUUGCUCGACUGUGAGGGGACCUACCCACUGUGGAAACACCACAGGAACUUCCAACCUUCGGGUGGCGAGGUAGGGCAGAAGAGUGACC
((((((((..((((..<...(((((((.((((((.....))))))...>..((.(((....)))))).))))))))))..))))))))

7.3.3 8t2b_R

Crystal structure of SCV PTE G18C mutant RNA in complex with Fab BL3-6

Release date: 2024-01-10
Method: x-ray diffraction
Resolution: 3.18 Å
Chain length: 90
Extracted length: 90

Total clashes: 49
Clashes per residue: 0.544
Clashscore: 9.440

Description: RNA (90-MER)
Organism: Saguaro cactus virus

Ojha, M., Vogt, J., Das, N.K., Redmond, E., Singh, K., Banna, H.A., Sadat, T., Koirala, D. (2024) Structure of saguaro cactus virus 3' translational enhancer mimics 5' cap for eIF4E binding. Proc.Natl.Acad.Sci.USA.
DOI: 10.1073/pnas.2313677121

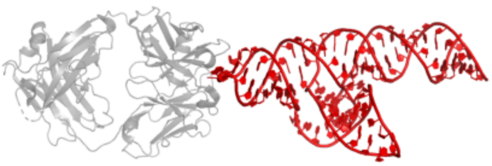

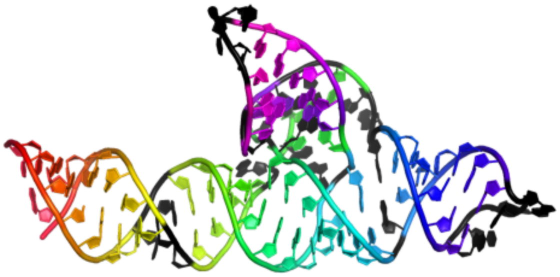

```
GGUUGCUCGACUGUGAGCGGACCUACCCACUGUGGAAACACCACAGGAACUUCCAACCUUCGGGUGGCGAGGUAGGGCAGAAGAGUGACC
 ((((((((..((((..<...(((((((.((((((.....))))))...>..((.(((....)))))).))))))))))..))))))))
```

7.4 ITS-2

7.4.1 7r6q_6

State E2 nucleolar 60S ribosome biogenesis intermediate - Foot region model

Release date: 2022-11-09
Method: electron microscopy
Resolution: 2.98 Å
Chain length: 87
Extracted length: 87

Total clashes: 0
Clashes per residue: 0.000
Clashscore: 6.420

Description: ITS-2
Organism: Saccharomyces cerevisiae BY4741

Cruz, V.E., Sekulski, K., Peddada, N., Sailer, C., Balasubramanian, S., Weirich, C.S., Stengel, F., Erzberger, J.P. (2022) Sequence-specific remodeling of a topologically complex RNP substrate by Spb4. Nat.Struct.Mol.Biol.
DOI: 10.1038/s41594-022-00874-9

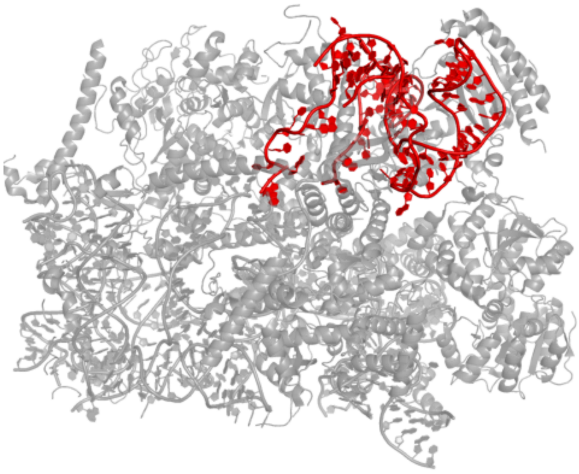

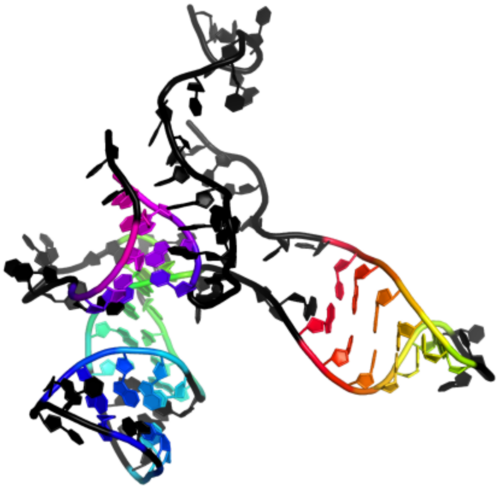

```
CCUUCUCAAACAUUCUGUUUGGUAGUGAGUGAUACUCUUUGGAGUUAACUUGAAAUUGCUGGCCUUUAGGCGAACAAUGUUCUUAAA
......((((((...))))))....(((((...(((.(...))))..))))..).......((((..))))................
```

7.4.2 7u0h_6

State NE1 nucleolar 60S ribosome biogenesis intermediate - Overall model

Release date: 2022-12-14
Method: electron microscopy
Resolution: 2.76 Å
Chain length: 87
Extracted length: 87

Total clashes: 60
Clashes per residue: 0.690
Clashscore: 7.920

Description: ITS2 rRNA
Organism: Saccharomyces cerevisiae BY4741

Cruz, V.E., Sekulski, K., Peddada, N., Sailer, C., Balasubramanian, S., Weirich, C.S., Stengel, F., Erzberger, J.P. (2022) Sequence-specific remodeling of a topologically complex RNP substrate by Spb4. Nat.Struct.Mol.Biol.
DOI: 10.1038/s41594-022-00874-9

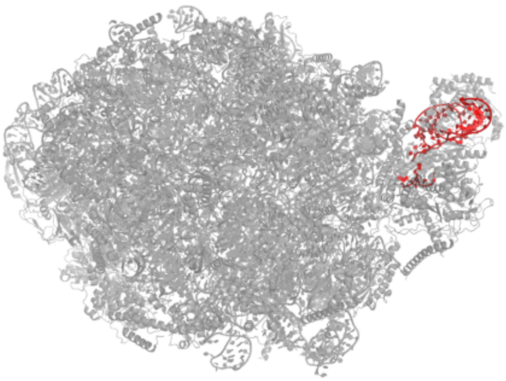

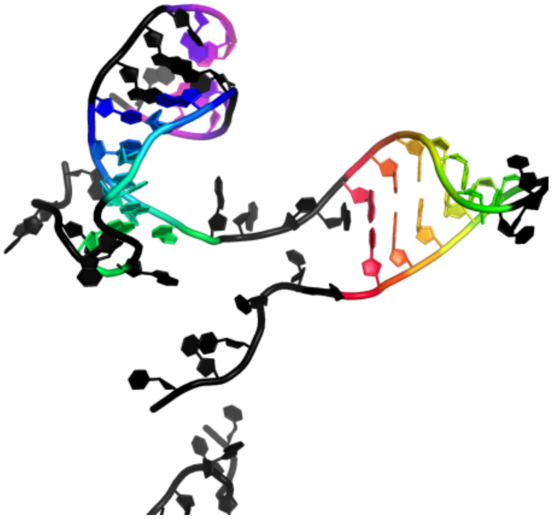

CCUUCUCAAACAUUCUGUUUGGUAGUGAGUGAUACUCUUUGGAGUUAACUUGAAAUUGCUGGCCUUUAGGCGAACAAUGUUCUUAAA
......((((((...))))))....(((((...(((......)))..))))..).................................

7.5 ENE

7.5.1 7ljy_B

Cryo-EM structure of the B dENE construct complexed with a 28-mer poly(A)

Release date: 2021-04-14
Method: electron microscopy
Resolution: 5.60 Å
Chain length: 76
Extracted length: 76

Total clashes: 5
Clashes per residue: 0.066
Clashscore: 1.860

Description: B dENE construct
Organism: Oryza sativa

Torabi, S.F., Chen, Y.L., Zhang, K., Wang, J., DeGregorio, S.J., Vaidya, A.T., Su, Z., Pabit, S.A., Chiu, W., Pollack, L., Steitz, J.A. (2021) Structural analyses of an RNA stability element interacting with poly(A). Proc.Natl.Acad.Sci.USA.
DOI: 10.1073/pnas.2026656118

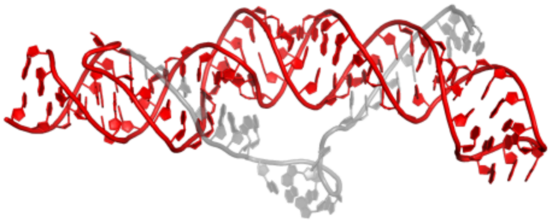

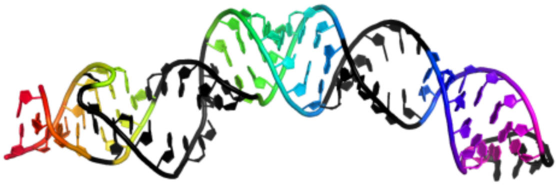

GGGUACUCUUUUCUUUGUCAUGGUUUUCUCAGGCGAAAGUCUGAGUUUUUACAUGACAAAGUUUUUAACGAGGCCC
((((.(((......((((((((.....(((((((....)))))))......))))))))..........)))))))

7.5.2 3p22_A

Crystal structure of the ENE, a viral RNA stability element, in complex with A9 RNA

Release date: 2010-12-08
Method: x-ray diffraction
Resolution: 2.50 Å
Chain length: 40
Extracted length: 40

Total clashes: 3
Clashes per residue: 0.075
Clashscore: 3.530

Description: Core ENE hairpin from KSHV PAN RNA
Organism: nan

Mitton-Fry, R.M., DeGregorio, S.J., Wang, J., Steitz, T.A., Steitz, J.A. (2010) Poly(A) tail recognition by a viral RNA element through assembly of a triple helix. Science.
DOI: 10.1126/science.1195858

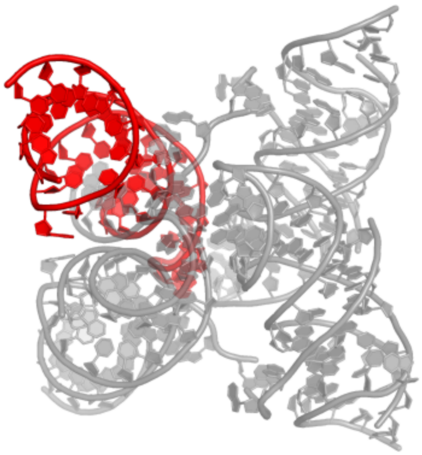

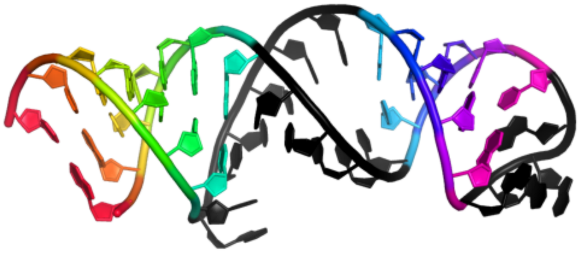

GGCUGGGUUUUUCCUUCGAAAGAAGGUUUUUAUCCCAGUC
(((((((.....(((((....))))).......)))))))

7.5.3 7jnh_B

Crystal structure of a double-ENE RNA stability element in complex with a 28-mer poly(A) RNA

Release date: 2021-01-20
Method: x-ray diffraction
Resolution: 2.89 Å
Chain length: 86
Extracted length: 86

Total clashes: 62
Clashes per residue: 0.721
Clashscore: 13.540

Description: Core double ENE RNA (Xtal construct) from Oryza sativa transposon,Core double ENE RNA (Xtal construct) from Oryza sativa transposon
Organism: Oryza sativa

Torabi, S.F., Vaidya, A.T., Tycowski, K.T., DeGregorio, S.J., Wang, J., Shu, M.D., Steitz, T.A., Steitz, J.A. (2021) RNA stabilization by a poly(A) tail 3'-end binding pocket and other modes of poly(A)-RNA interaction. Science.
DOI: 10.1126/science.abe6523

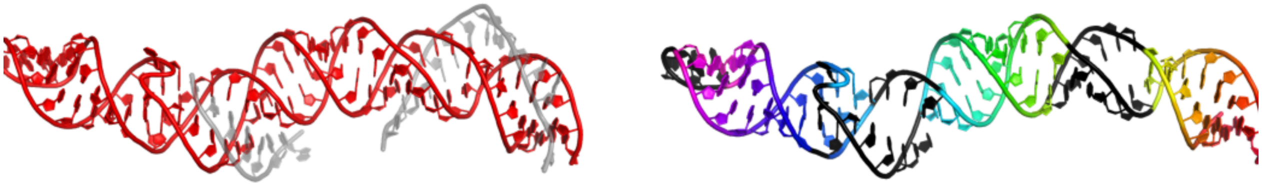

GGGCUGAGUUUUUACAUGACAAAGUUUUUAACGAGGCAGCGGCGAAAGUCGCUGUACUCUUUUCUUUGUCAUGGUUUUCUCAGCCC
((((((((......((((((((((........(((((((((((....)))))))).)))....))))))))))).....))))))))

7.5.4 3p22_E

Crystal structure of the ENE, a viral RNA stability element, in complex with A9 RNA

Release date: 2010-12-08
Method: x-ray diffraction
Resolution: 2.50 Å
Chain length: 40
Extracted length: 40

Total clashes: 8
Clashes per residue: 0.200
Clashscore: 3.530

Description: Core ENE hairpin from KSHV PAN RNA
Organism: nan

Mitton-Fry, R.M., DeGregorio, S.J., Wang, J., Steitz, T.A., Steitz, J.A. (2010) Poly(A) tail recognition by a viral RNA element through assembly of a triple helix. Science.
DOI: 10.1126/science.1195858

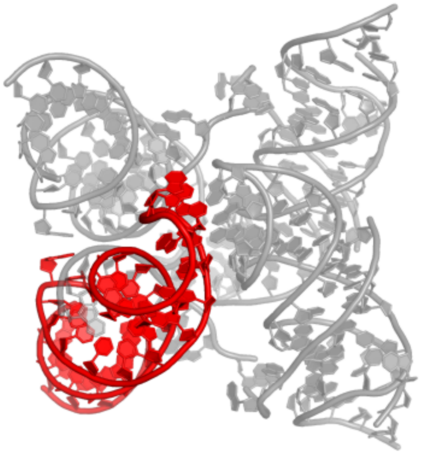

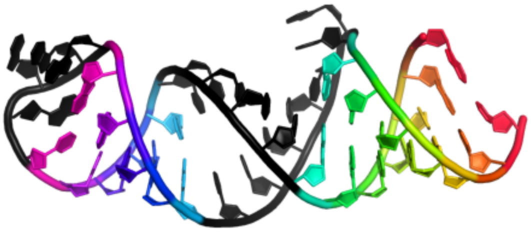

GGCUGGGUUUUUCCUUCGAAAGAAGGUUUUUAUCCCAGUC
((((((.....(((((....))))).......))))))

7.6 ASH1 mRNA E3-localization element

7.6.1 5m0h_A

Crystal structure of the central flexible region of ASH1 mRNA E3-localization element

Release date: 2017-01-18
Method: x-ray diffraction
Resolution: 2.65 Å
Chain length: 42
Extracted length: 42

Total clashes: 10
Clashes per residue: 0.238
Clashscore: 3.700

Description: ASH1 E3 (42 nt-TL/TLR)
Organism: Saccharomyces cerevisiae

Edelmann, F.T., Schlundt, A., Heym, R.G., Jenner, A., Niedner-Boblenz, A., Syed, M.I., Paillart, J.C., Stehle, R., Janowski, R., Sattler, M., Jansen, R.P., Niessing, D. (2017) Molecular architecture and dynamics of ASH1 mRNA recognition by its mRNA-transport complex. Nat. Struct. Mol. Biol.
DOI: 10.1038/nsmb.3351

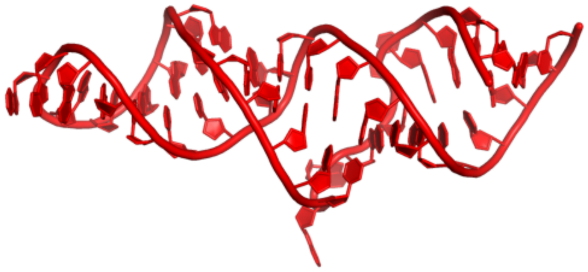

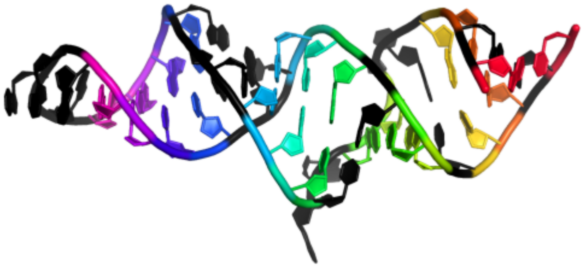

GAUAACUGAAUCGCUAAGGAUGAAAGUCUAUGCGACAUUAUC
(.((..((...(((...((((....))))..))).)).)).)

7.7 SCNMV xrRNA

7.7.1 6d3p_A

Crystal structure of an exoribonuclease-resistant RNA from Sweet clover necrotic mosaic virus (SCNMV)

Release date: 2018-06-20
Method: x-ray diffraction
Resolution: 2.90 Å
Chain length: 45
Extracted length: 45

Total clashes: 18
Clashes per residue: 0.400
Clashscore: 6.560

Description: RNA (45-MER)
Organism: Sweet clover necrotic mosaic virus

Steckelberg, A.L., Akiyama, B.M., Costantino, D.A., Sit, T.L., Nix, J.C., Kieft, J.S. (2018) A folded viral noncoding RNA blocks host cell exoribonucleases through a conformationally dynamic RNA structure. Proc. Natl. Acad. Sci. U.S.A.
DOI: 10.1073/pnas.1802429115

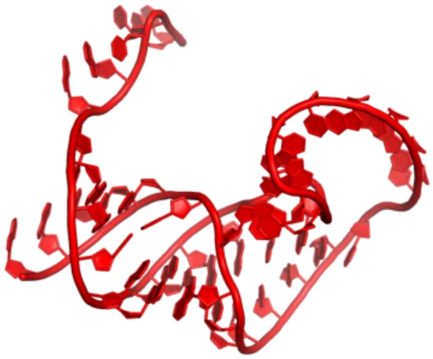

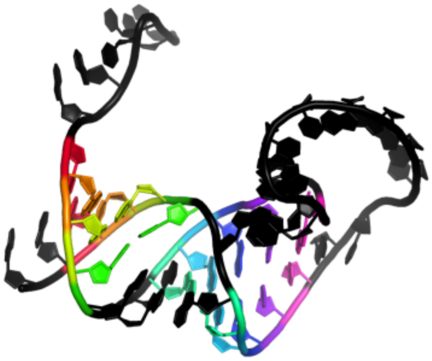

```
GGGCGUAACCUCCAUCCGAGUUGCAAGAGAGGGAAACGCAGUCUC
..((((..(((((.............).))))...))))......
```

7.8 Influenza B vRNA promoter

7.8.1 6t2c_V

Bat Influenza A polymerase recycling complex

Release date: 2020-04-15
Method: electron microscopy
Resolution: 3.52 Å
Chain length: 34
Extracted length: 34

Total clashes: 0
Clashes per residue: 0.000
Clashscore: 4.190

Description: vRNA
Organism: Influenza B virus

Wandzik, J.M., Kouba, T., Karuppasamy, M., Pflug, A., Drncova, P., Provaznik, J., Azevedo, N., Cusack, S. (2020) A Structure-Based Model for the Complete Transcription Cycle of Influenza Polymerase. Cell.
DOI: 10.1016/j.cell.2020.03.061

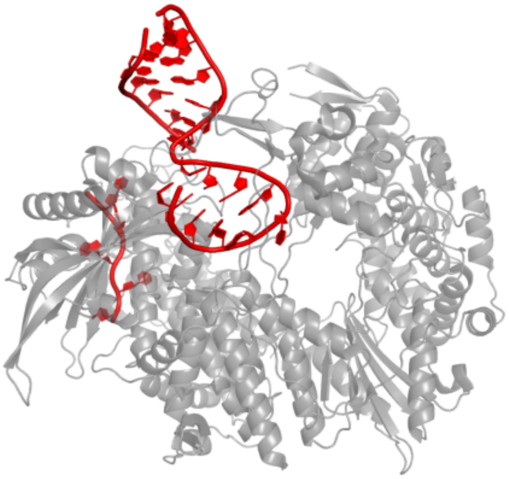

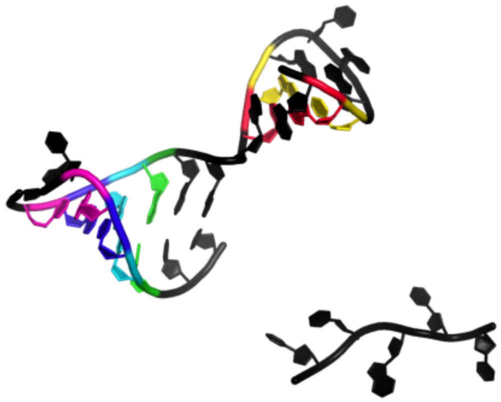

```
AGUAGUAACAAGAGGUAUUACCUCUGCUUCUGCU
.((....))...((((...))))...........
```

7.9 NAD-II riboswitch

7.9.1 8hb8_A

Crystal structure of NAD-II riboswitch (single strand) with NMN

Release date: 2023-03-22
Method: x-ray diffraction
Resolution: 2.30 Å
Chain length: 55
Extracted length: 55

Total clashes: 8
Clashes per residue: 0.145
Clashscore: 2.180

Description: RNA (55-MER)
Organism: Streptococcus parasanguinis

Peng, X., Liao, W., Lin, X., Lilley, D.M.J., Huang, L. (2023) Crystal structures of the NAD+-II riboswitch reveal two distinct ligand-binding pockets. Nucleic Acids Res.
DOI: 10.1093/nar/gkad102

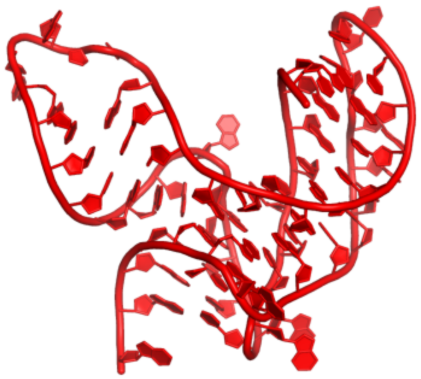

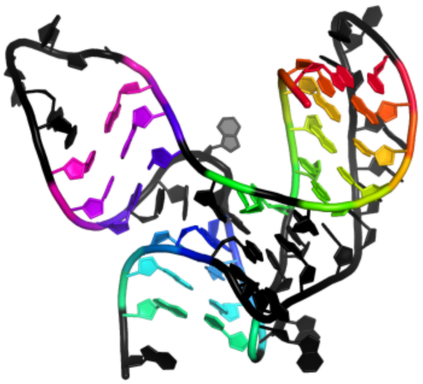

```
GCGGCGUUGCGUCCGAAAGUCUAAACAGACACGGCCGCUUAAAAACAAAAGGAGA
((((((...<.<<<....(((......))).).))))))............>>>>.
```

7.9.2 8hba_A

Crystal structure of NAD-II riboswitch (single strand) with NAD

Release date: 2023-03-22
Method: x-ray diffraction
Resolution: 2.64 Å
Chain length: 56
Extracted length: 56

Total clashes: 53
Clashes per residue: 0.946
Clashscore: 15.750

Description: RNA (55-MER)
Organism: Streptococcus parasanguinis

Peng, X., Liao, W., Lin, X., Lilley, D.M.J., Huang, L. (2023) Crystal structures of the NAD+-II riboswitch reveal two distinct ligand-binding pockets. Nucleic Acids Res.
DOI: 10.1093/nar/gkad102

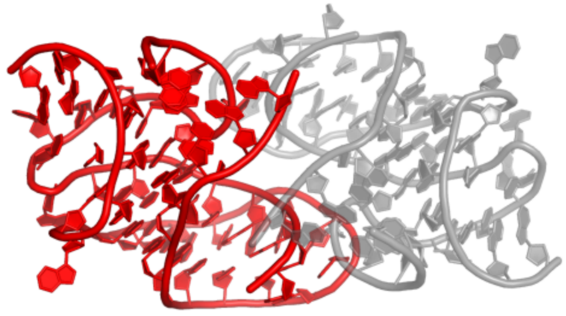

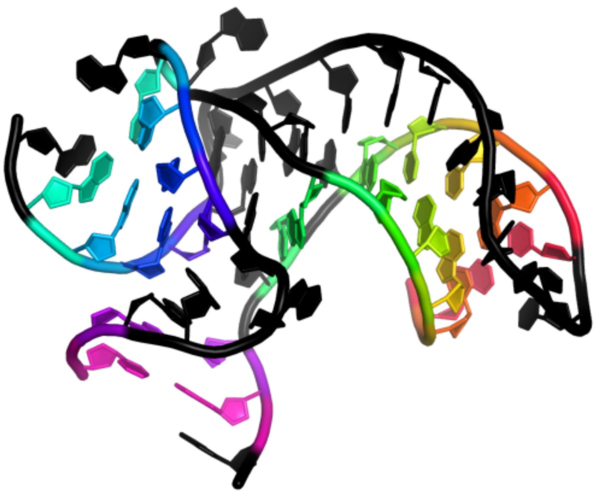

```
CGCGGCGUUGCGUCCGAAAGUCUAAACAGACACGGCCGCUUAAAAACAAAAGGAGA
.((((((...<.<<<....((........)).).)))))............>>>>.
```
